# Supplementary figures and images for: Targeting the transcription factor YY1 is synthetic lethal with loss of the histone demethylase KDM5C
Source: EMBO Rep. 2024 Oct 21;25(12):5408–28. doi: 10.1038/s44319-024-00290-8 (PMC11624269; doi:10.1038/s44319-024-00290-8)

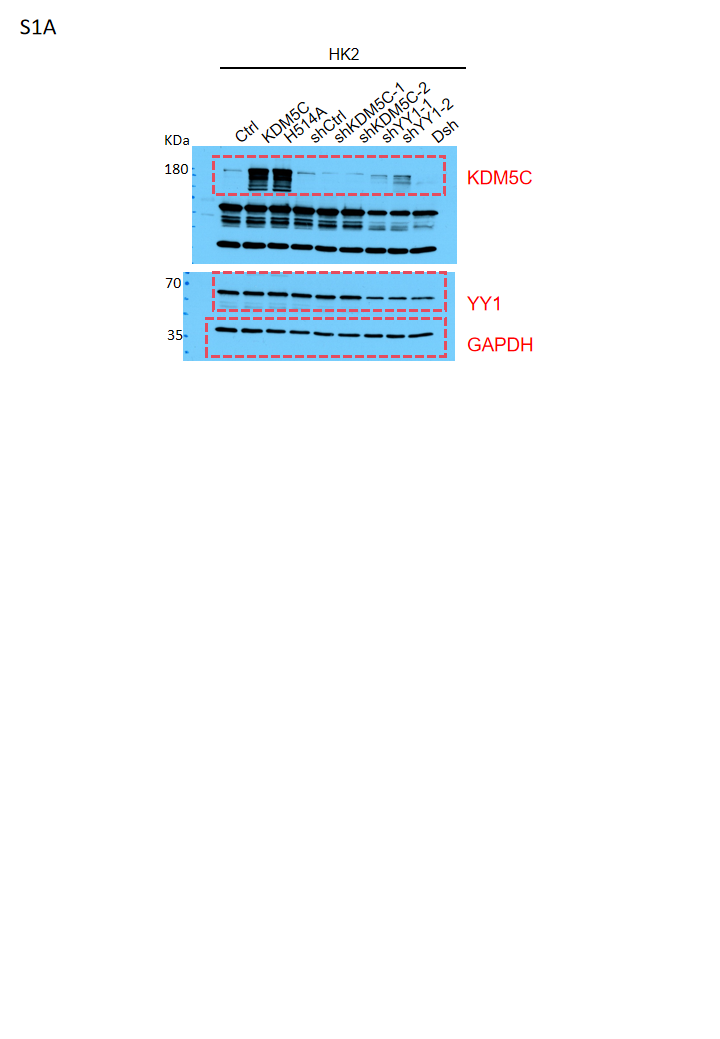

Supplement: Supplementary file 2 — EV Figures Source Data [file 44319_2024_290_MOESM2_ESM.zip › Figure EV1/EV1A.tif]

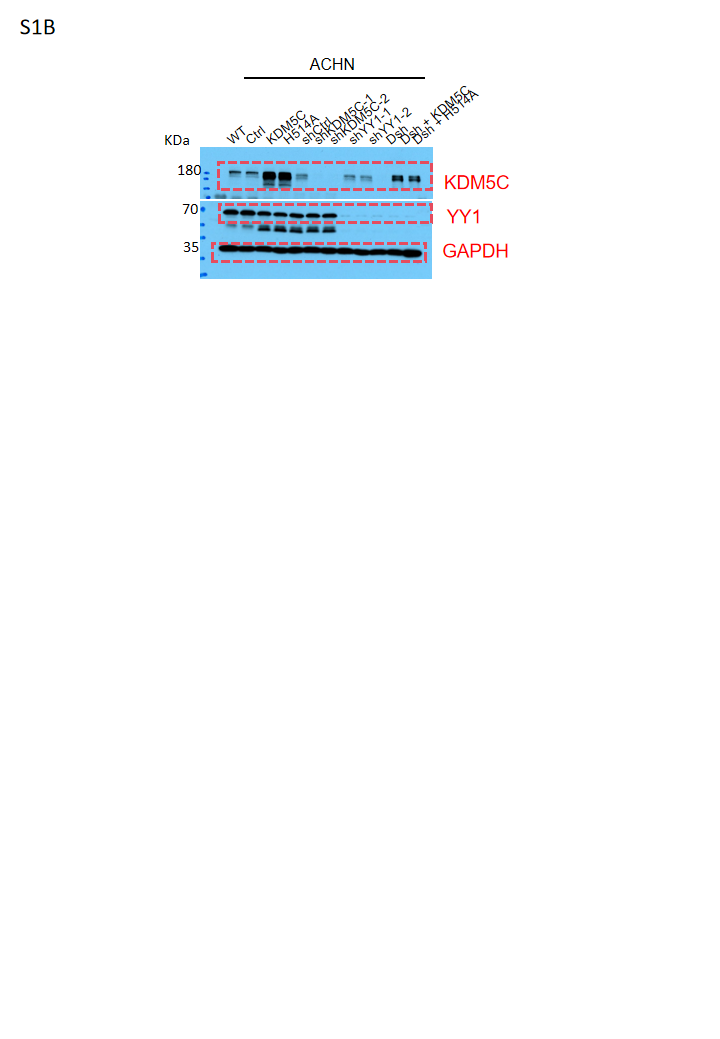

Supplement: Supplementary file 2 — EV Figures Source Data [file 44319_2024_290_MOESM2_ESM.zip › Figure EV1/EV1B.tif]

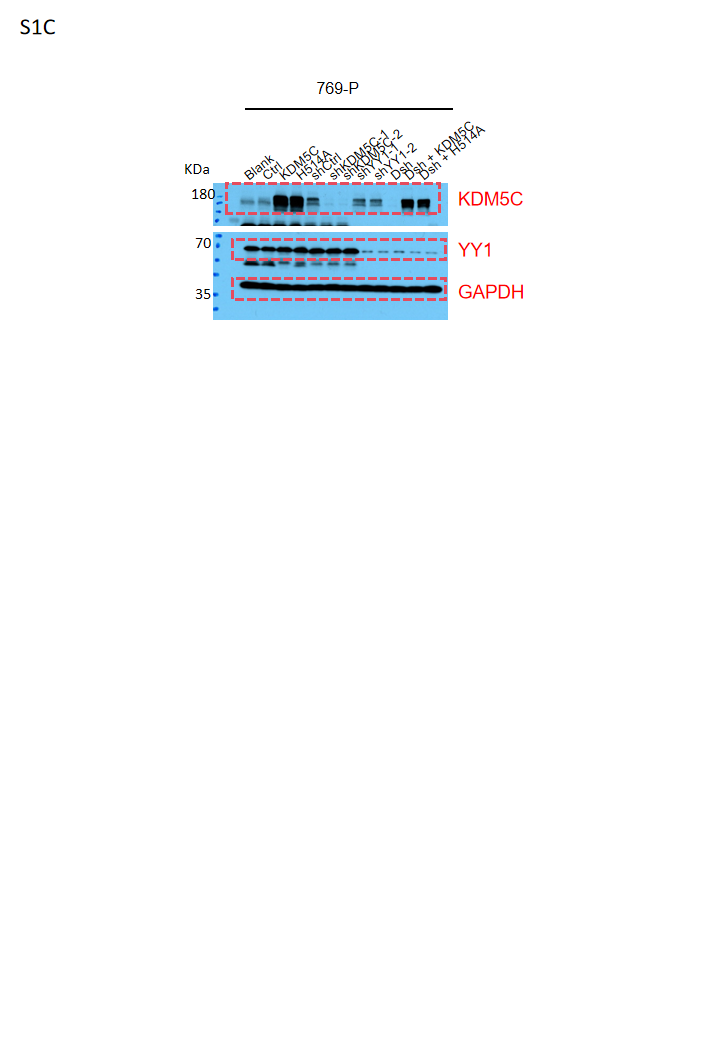

Supplement: Supplementary file 2 — EV Figures Source Data [file 44319_2024_290_MOESM2_ESM.zip › Figure EV1/EV1C.tif]

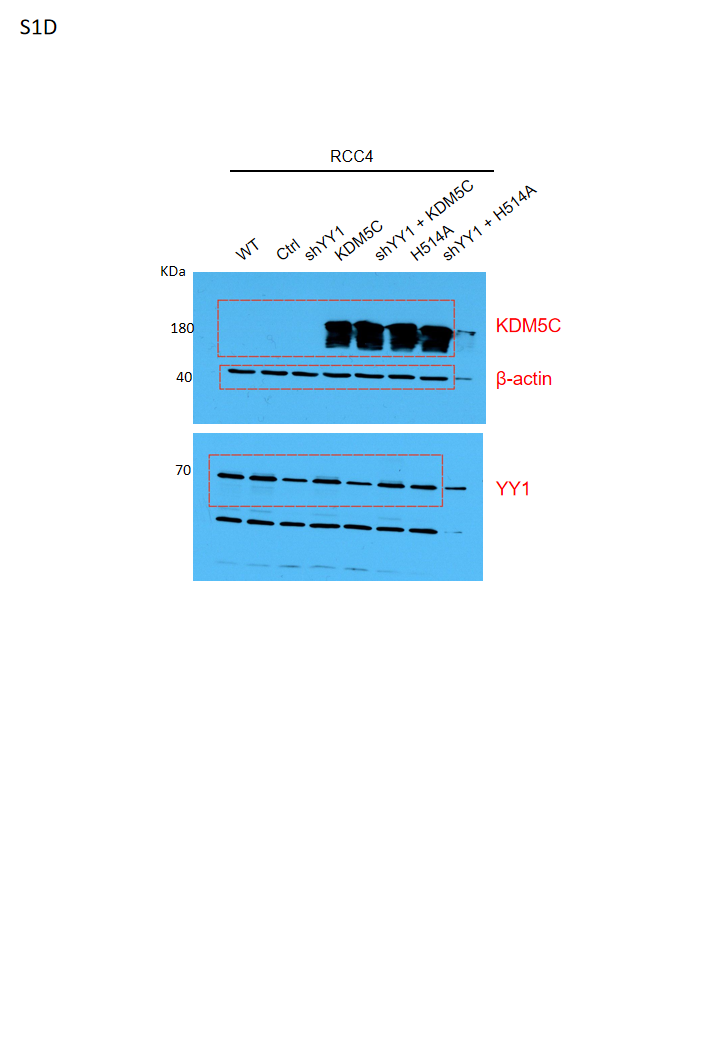

Supplement: Supplementary file 2 — EV Figures Source Data [file 44319_2024_290_MOESM2_ESM.zip › Figure EV1/EV1D.tif]

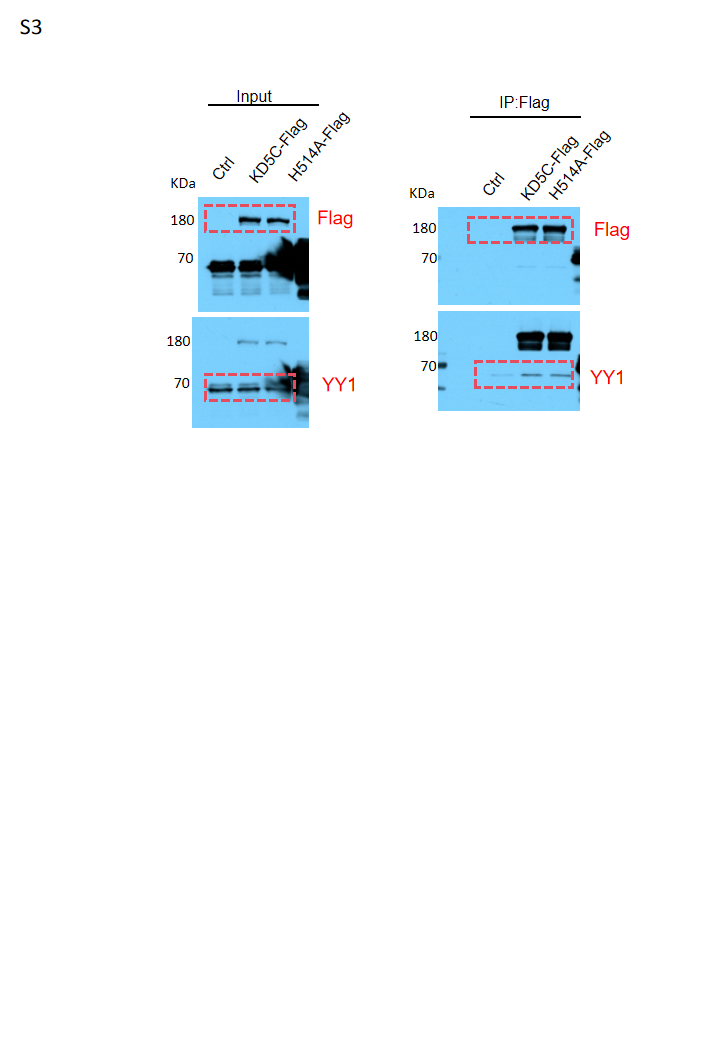

Supplement: Supplementary file 2 — EV Figures Source Data [file 44319_2024_290_MOESM2_ESM.zip › Figure EV3/EV3.tif]

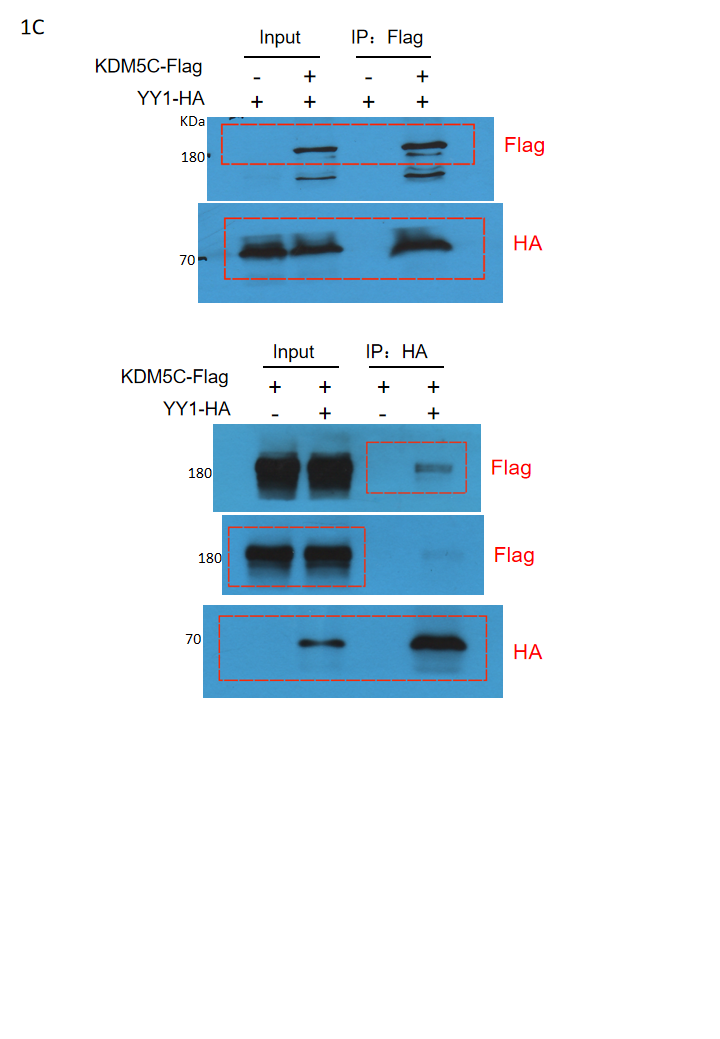

Supplement: Supplementary file 3 — Source data Fig. 1 [file 44319_2024_290_MOESM3_ESM.zip › 1C/1C.tif]

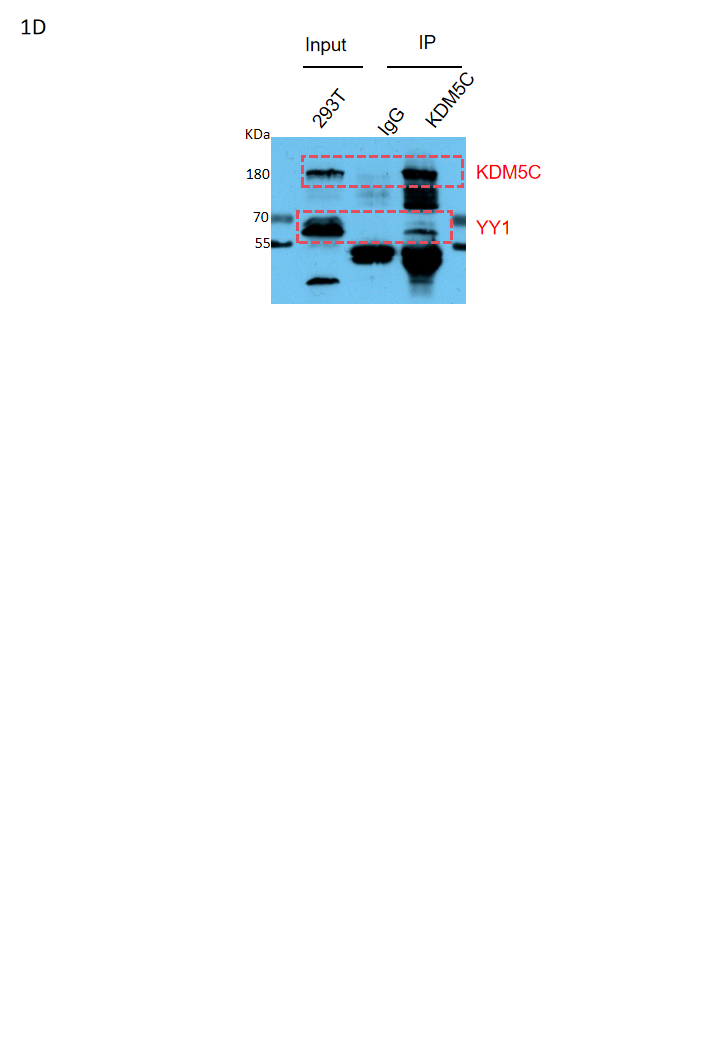

Supplement: Supplementary file 3 — Source data Fig. 1 [file 44319_2024_290_MOESM3_ESM.zip › 1D/1D.tif]

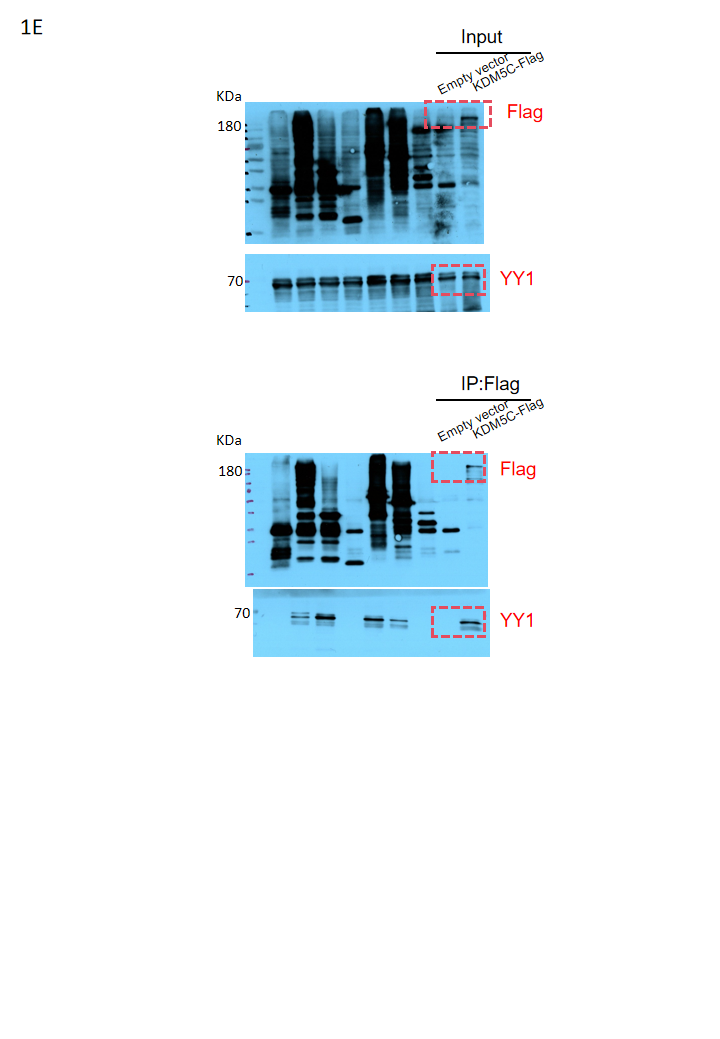

Supplement: Supplementary file 3 — Source data Fig. 1 [file 44319_2024_290_MOESM3_ESM.zip › 1E/1E.tif]

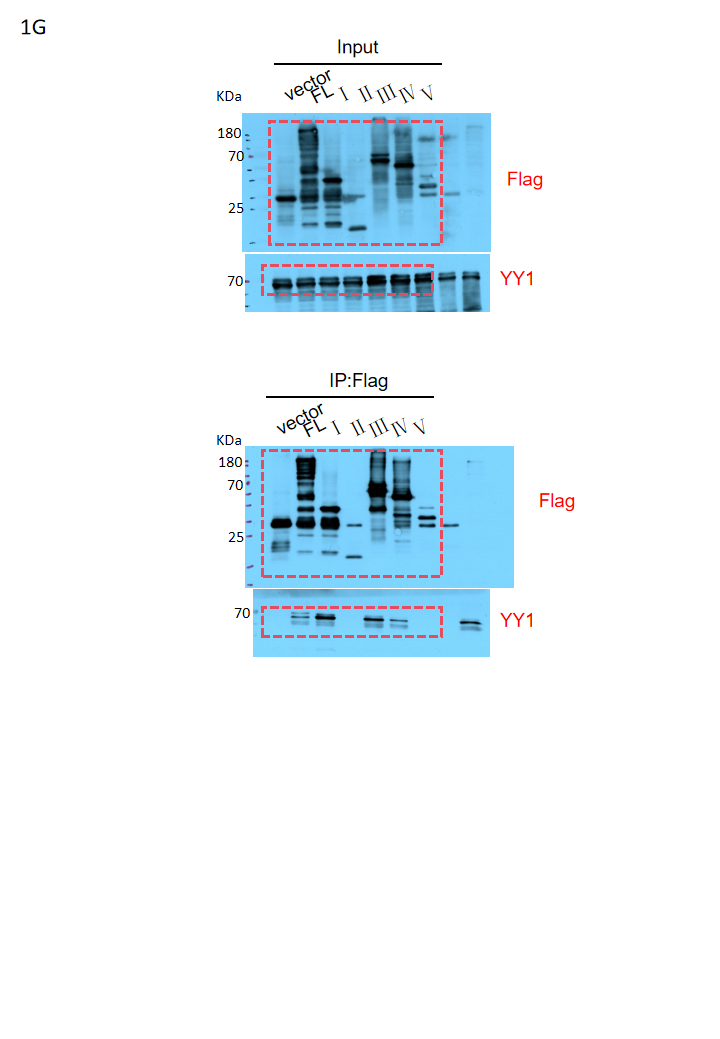

Supplement: Supplementary file 3 — Source data Fig. 1 [file 44319_2024_290_MOESM3_ESM.zip › 1G/1G.tif]

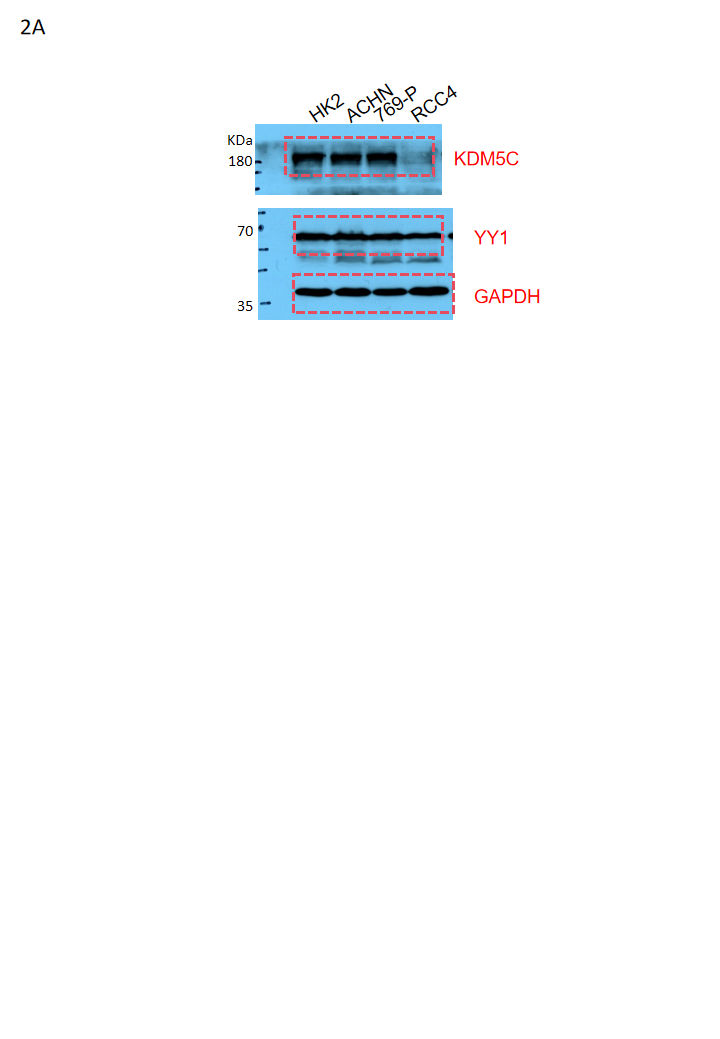

Supplement: Supplementary file 4 — Source data Fig. 2 [file 44319_2024_290_MOESM4_ESM.zip › 2A/2A.tif]

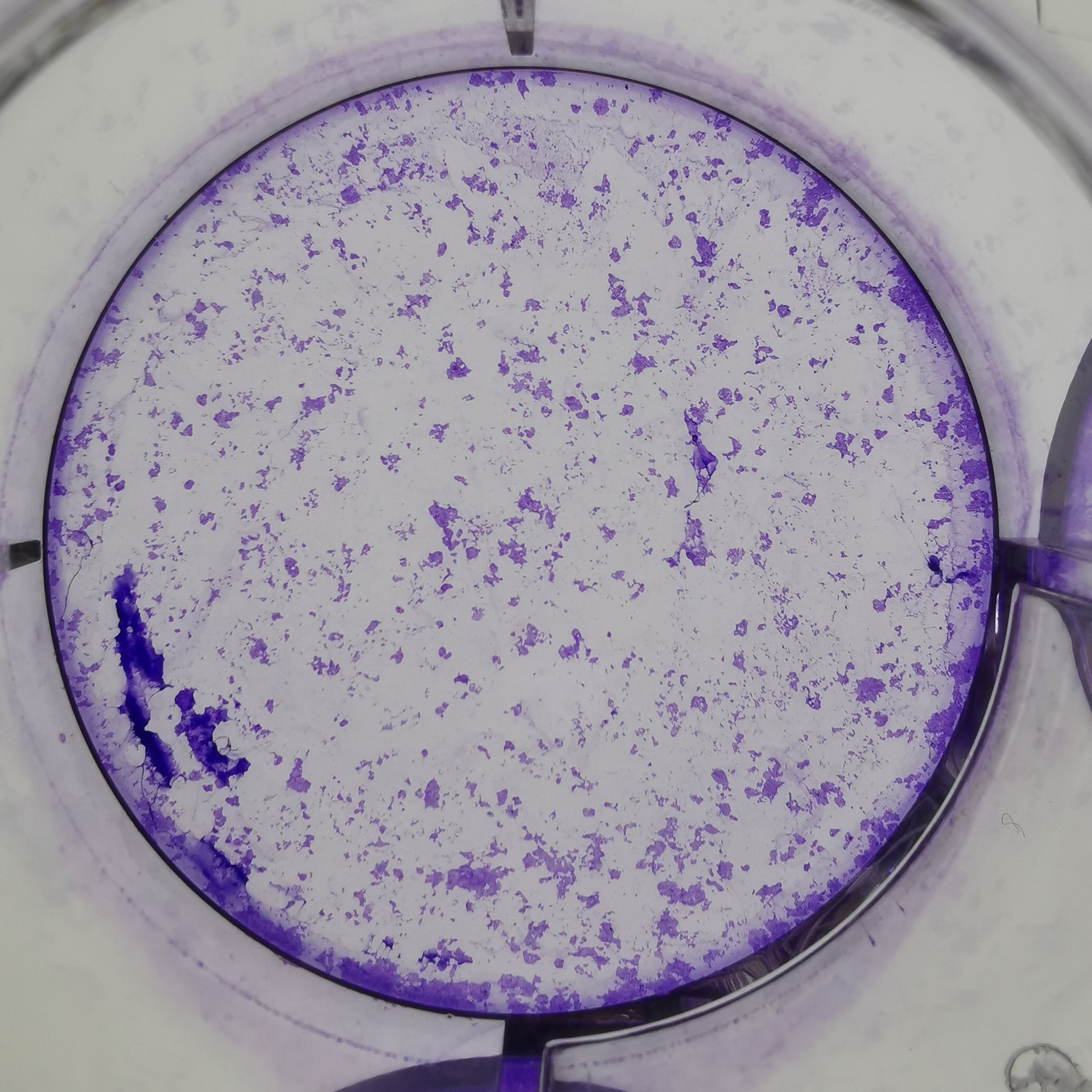

Supplement: Supplementary file 4 — Source data Fig. 2 [file 44319_2024_290_MOESM4_ESM.zip › 2B/769-P/shCtrl.tif]

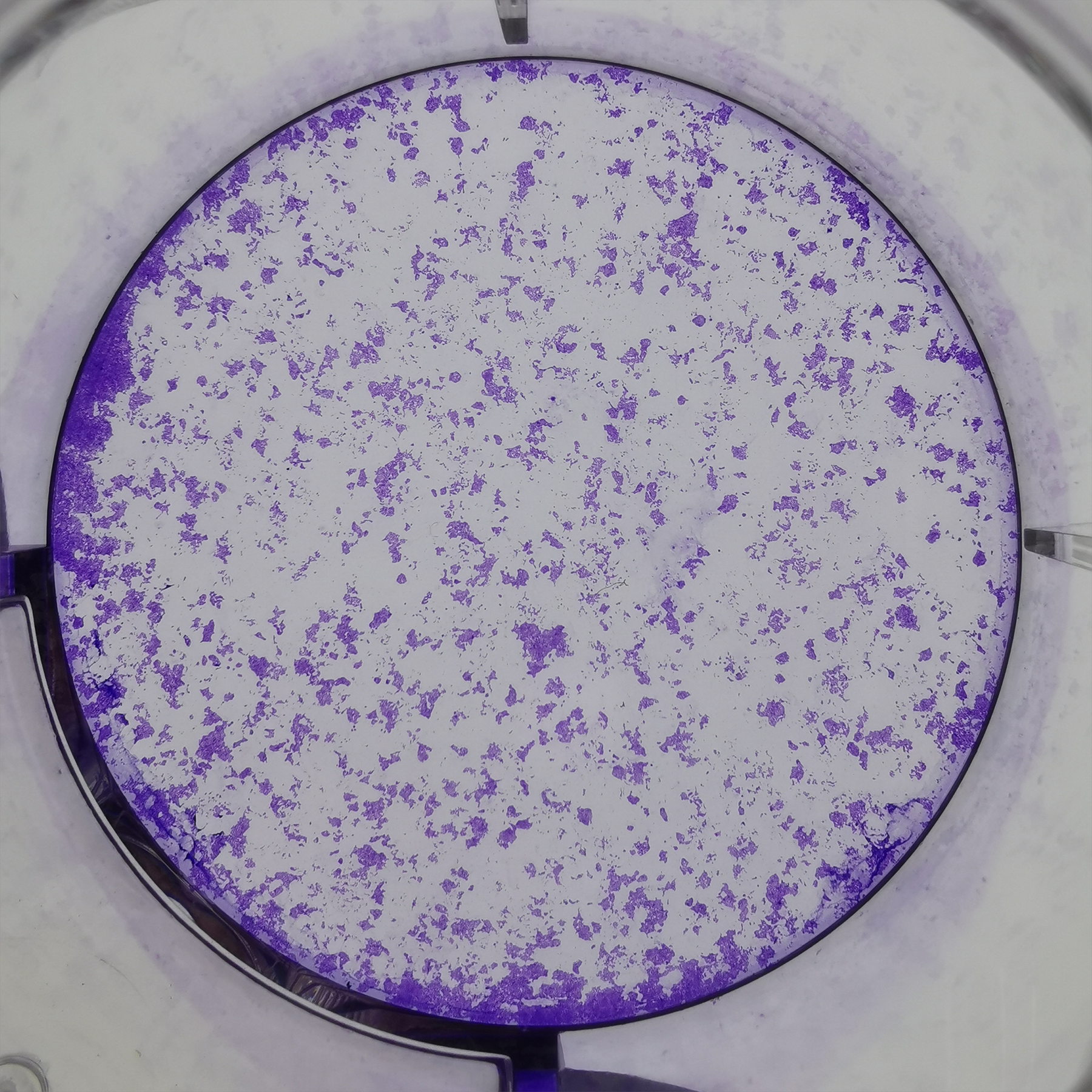

Supplement: Supplementary file 4 — Source data Fig. 2 [file 44319_2024_290_MOESM4_ESM.zip › 2B/769-P/shYY1-1.tif]

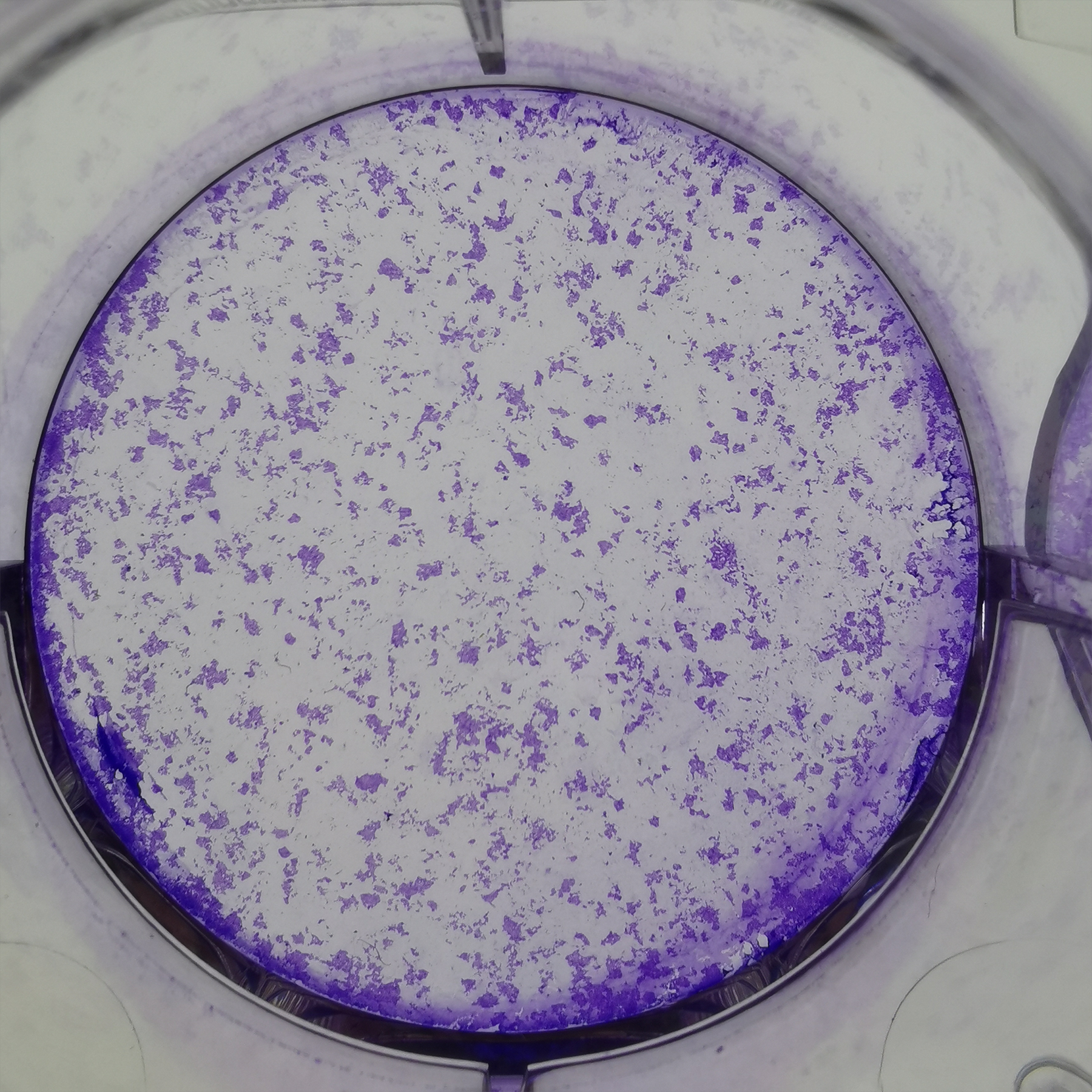

Supplement: Supplementary file 4 — Source data Fig. 2 [file 44319_2024_290_MOESM4_ESM.zip › 2B/769-P/shYY1-2.tif]

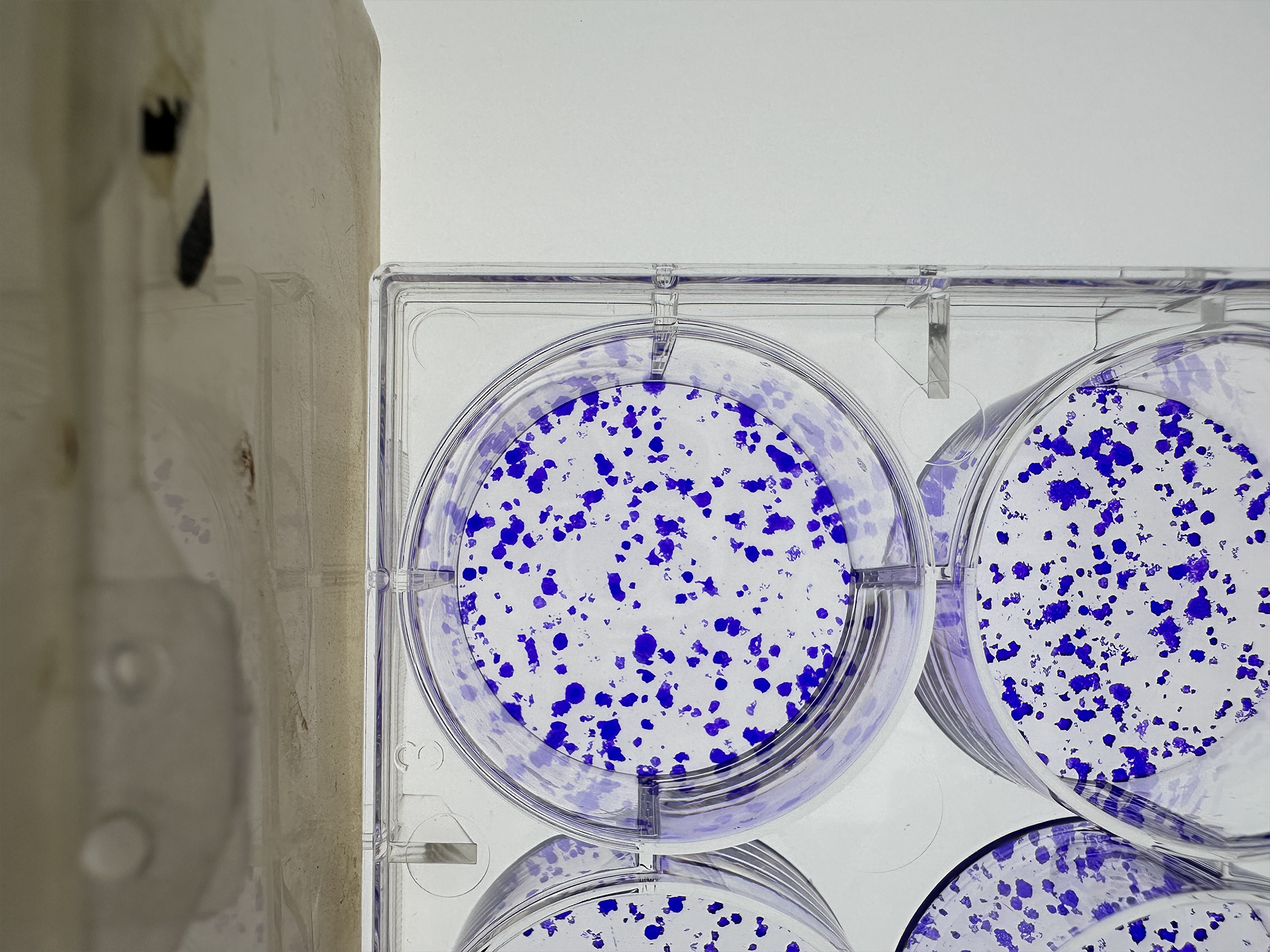

Supplement: Supplementary file 4 — Source data Fig. 2 [file 44319_2024_290_MOESM4_ESM.zip › 2B/ACHN/shCtrl.tif]

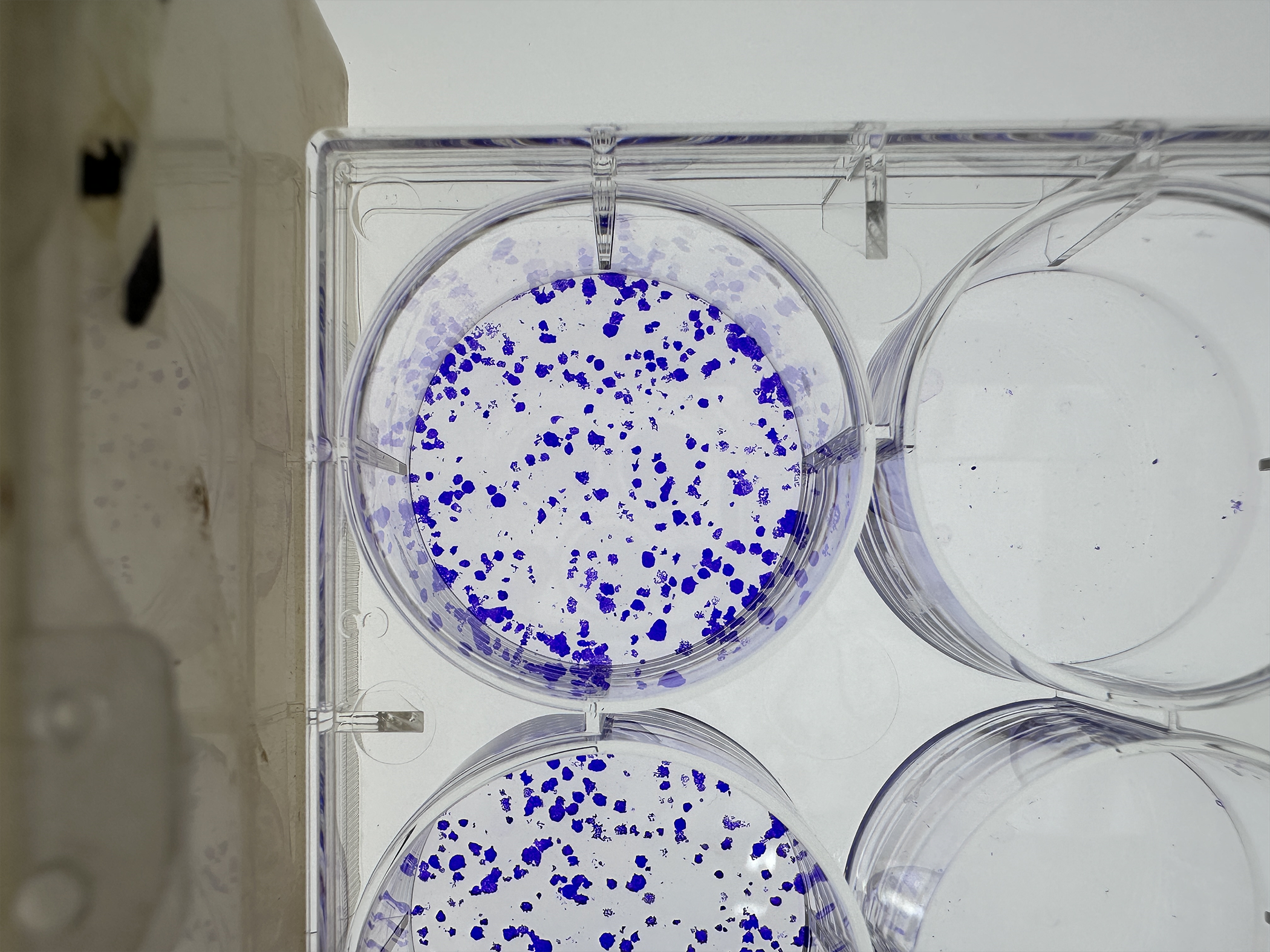

Supplement: Supplementary file 4 — Source data Fig. 2 [file 44319_2024_290_MOESM4_ESM.zip › 2B/ACHN/shYY1-1.tif]

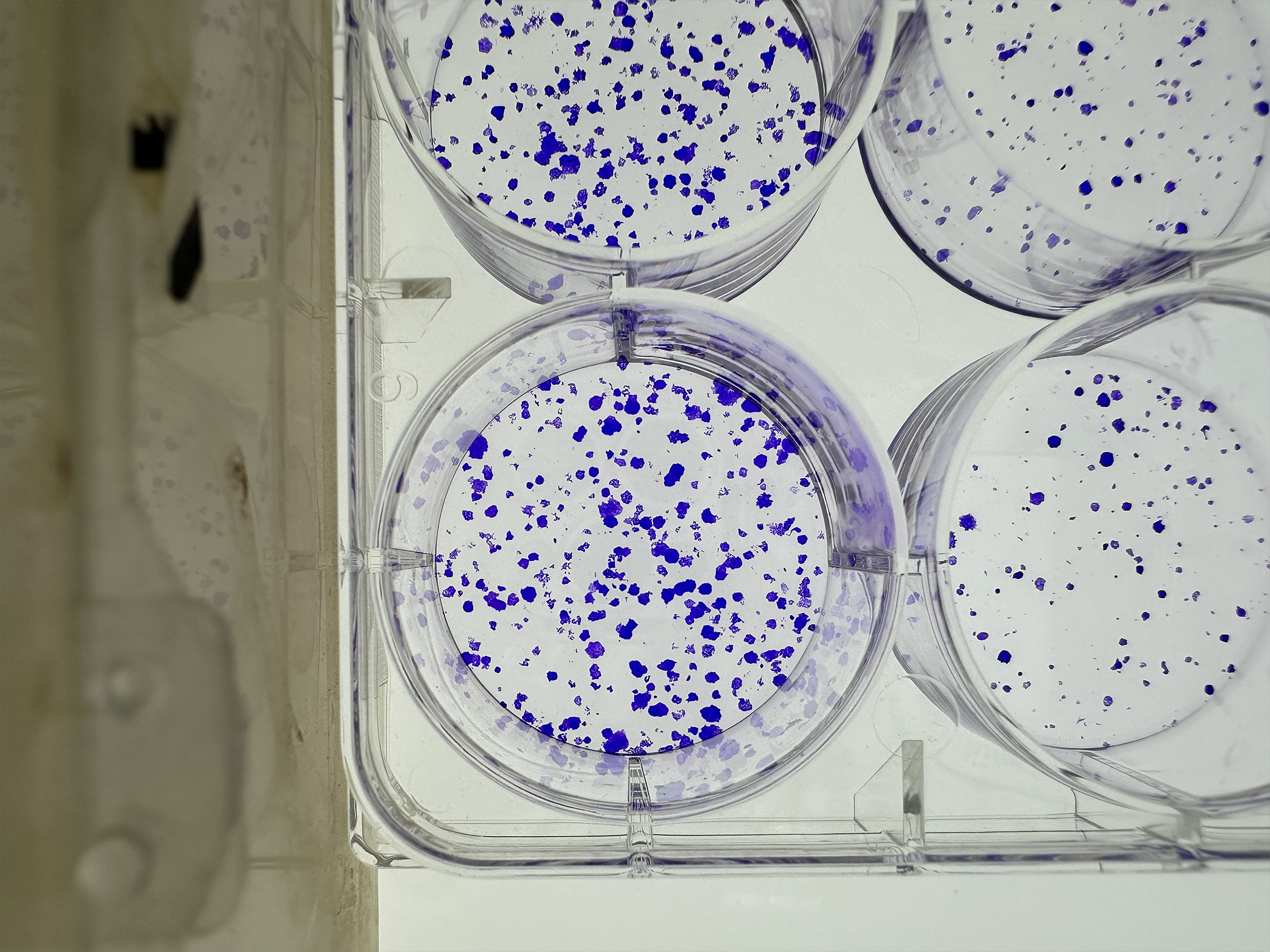

Supplement: Supplementary file 4 — Source data Fig. 2 [file 44319_2024_290_MOESM4_ESM.zip › 2B/ACHN/shYY1-2.tif]

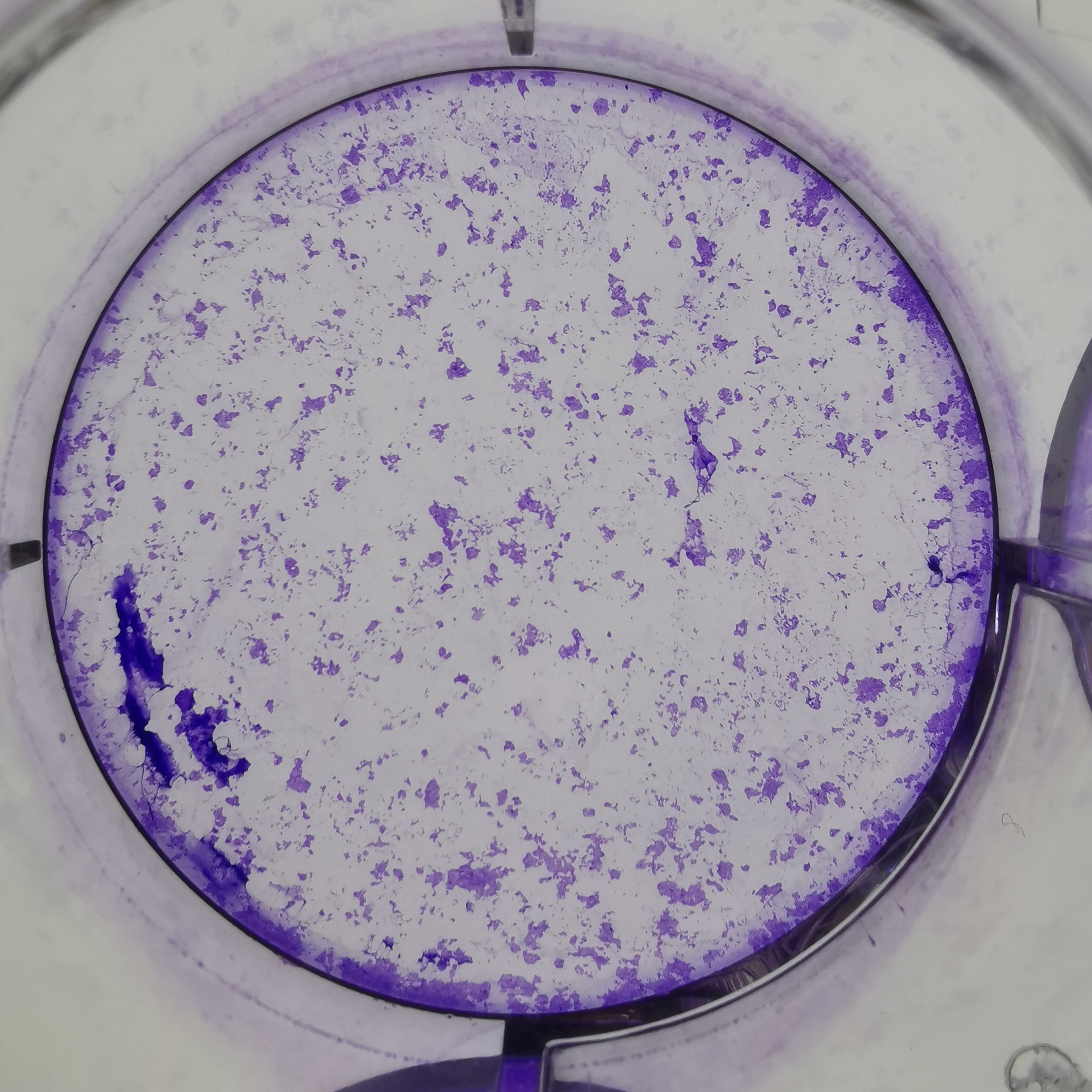

Supplement: Supplementary file 4 — Source data Fig. 2 [file 44319_2024_290_MOESM4_ESM.zip › 2B/Figure 2B-replicate/769-P/769P 2500 PLKO.1 (1).jpg]

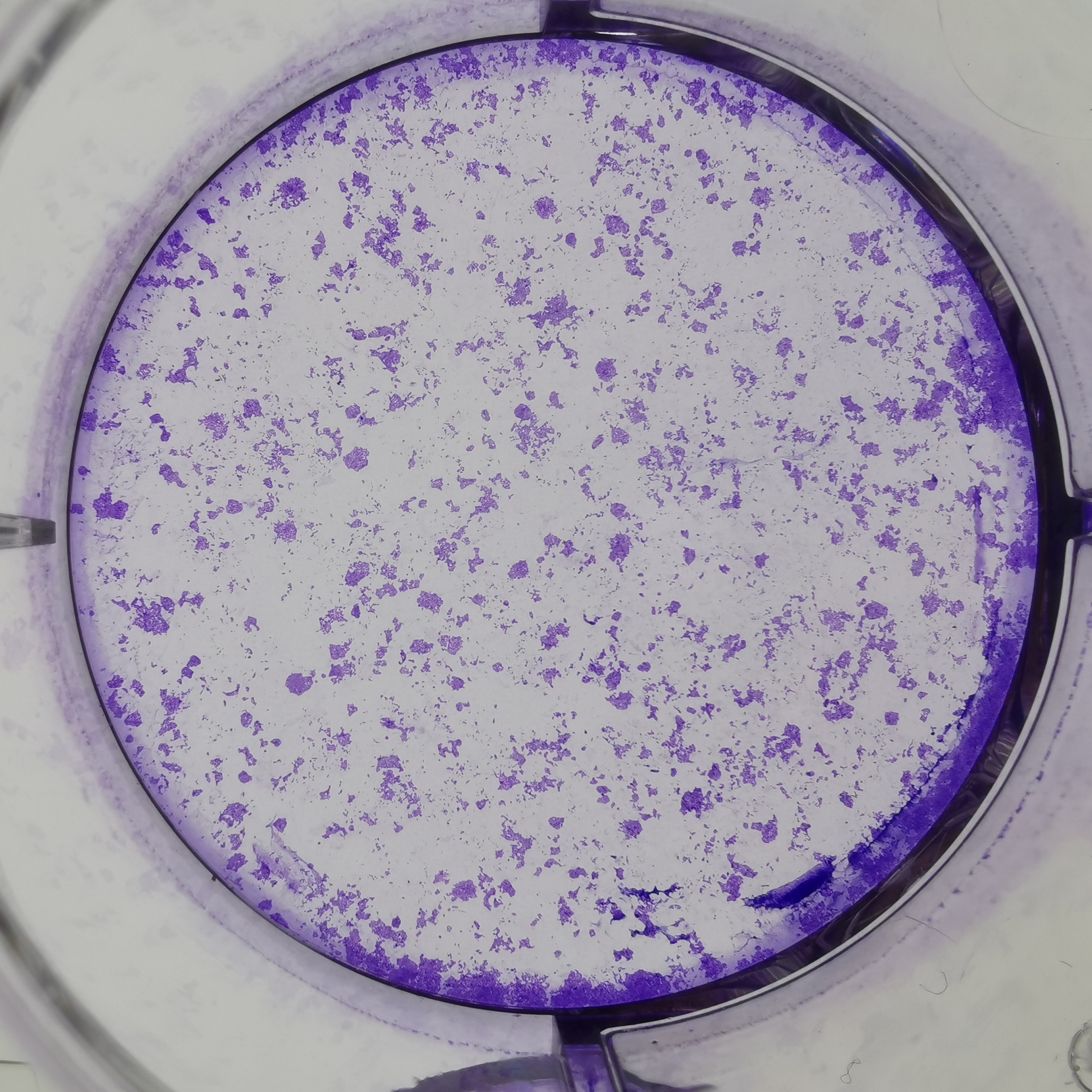

Supplement: Supplementary file 4 — Source data Fig. 2 [file 44319_2024_290_MOESM4_ESM.zip › 2B/Figure 2B-replicate/769-P/769P 2500 PLKO.1 (2).jpg]

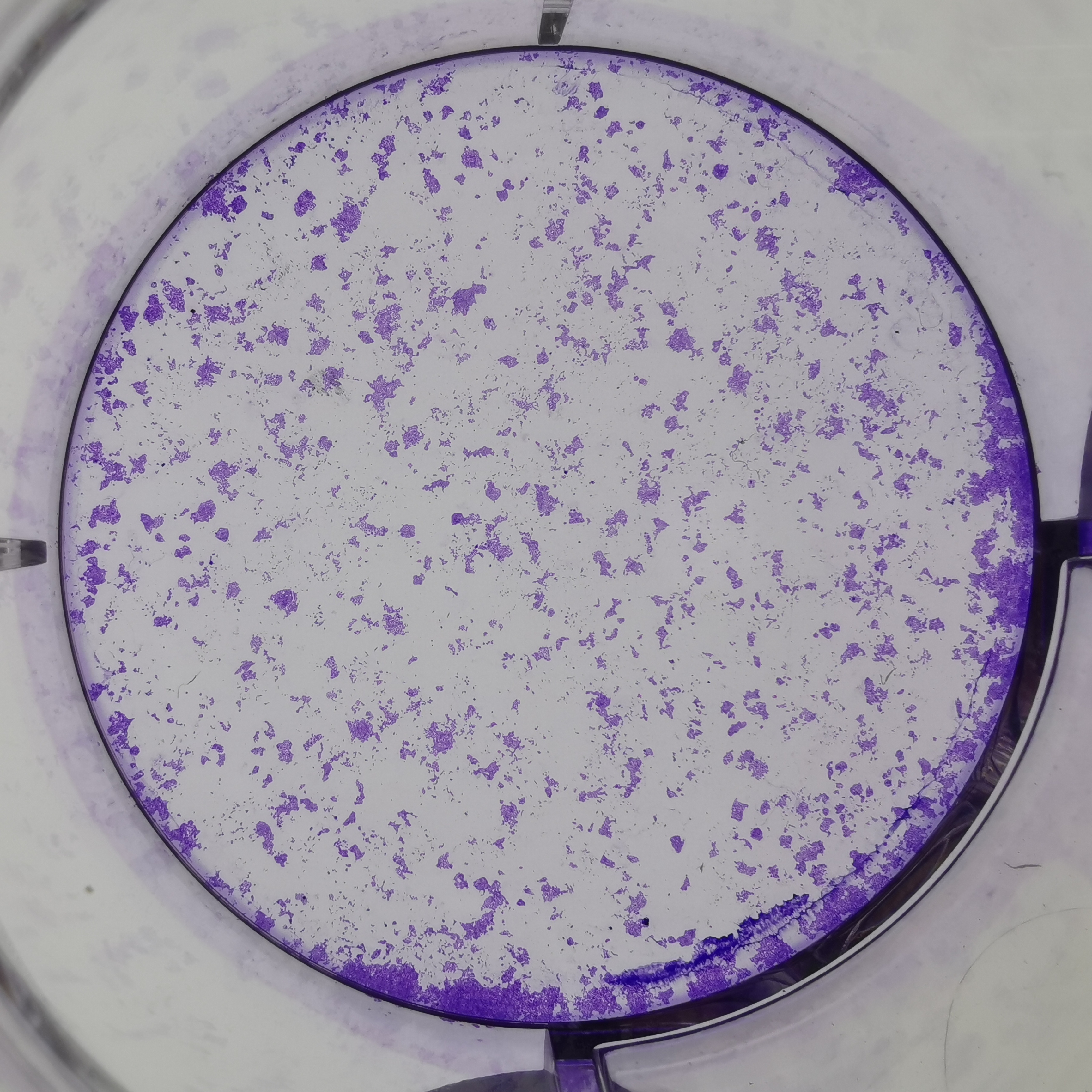

Supplement: Supplementary file 4 — Source data Fig. 2 [file 44319_2024_290_MOESM4_ESM.zip › 2B/Figure 2B-replicate/769-P/769P 2500 PLKO.1 (3).jpg]

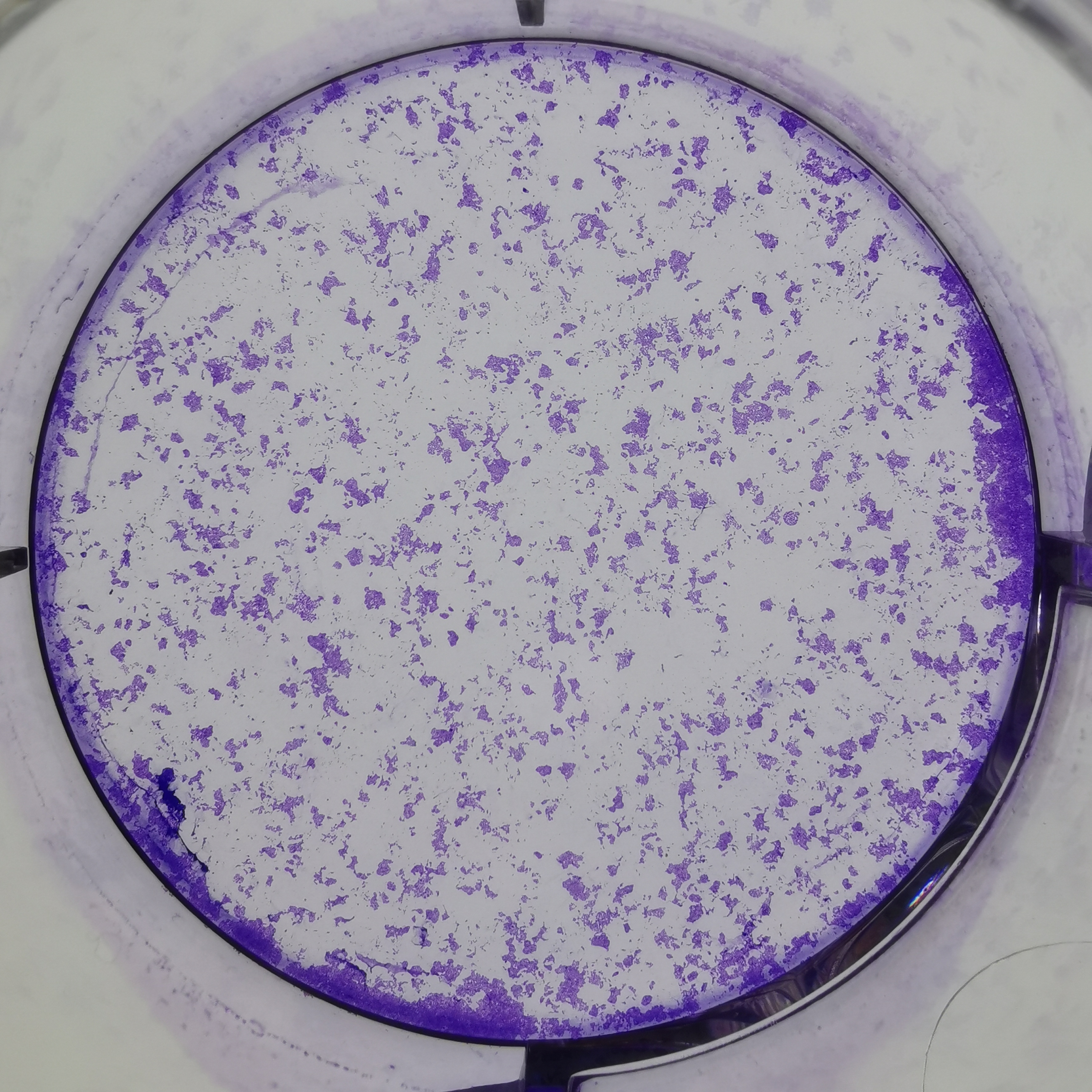

Supplement: Supplementary file 4 — Source data Fig. 2 [file 44319_2024_290_MOESM4_ESM.zip › 2B/Figure 2B-replicate/769-P/769P 2500 SHYY1-4 (1).jpg]

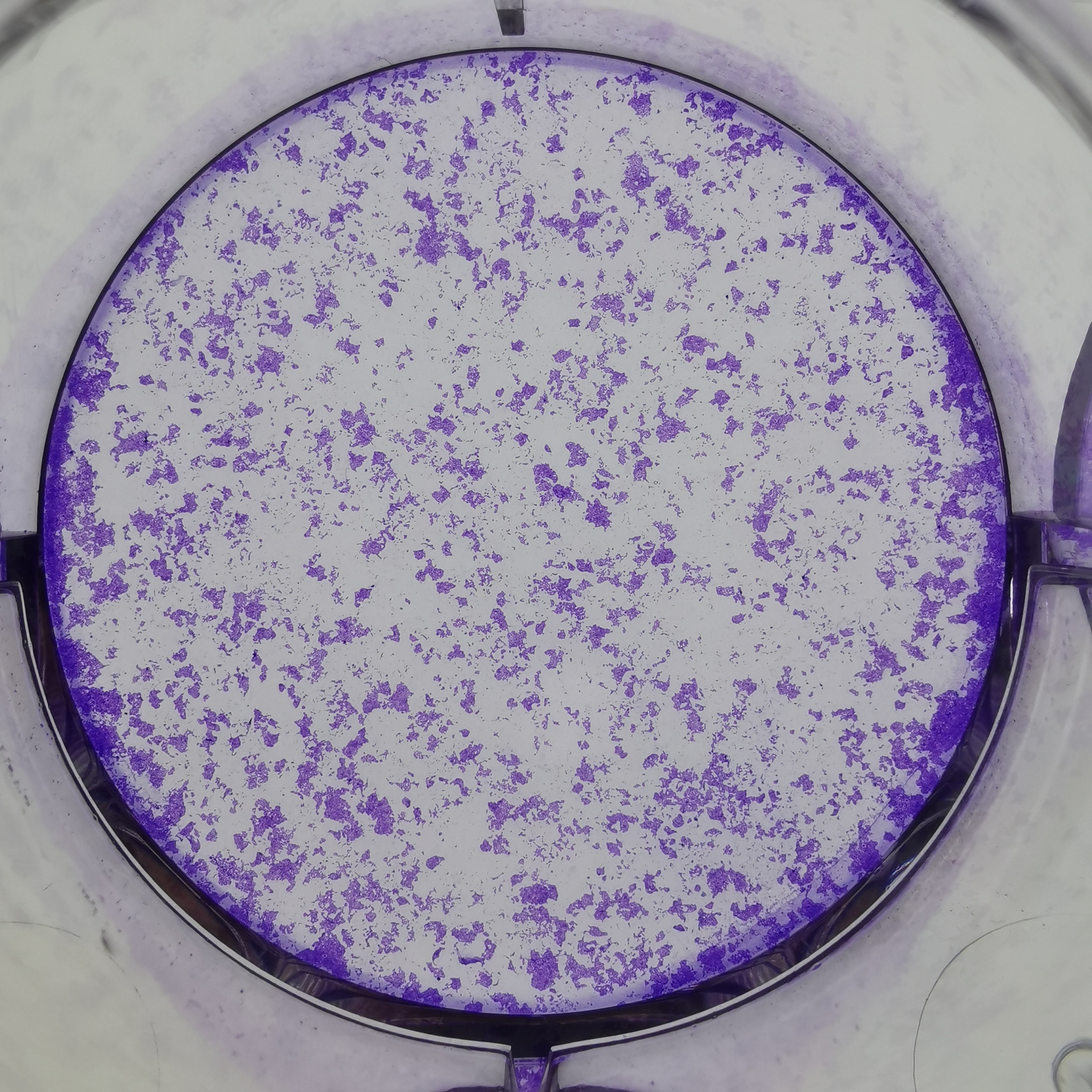

Supplement: Supplementary file 4 — Source data Fig. 2 [file 44319_2024_290_MOESM4_ESM.zip › 2B/Figure 2B-replicate/769-P/769P 2500 SHYY1-4 (2).jpg]

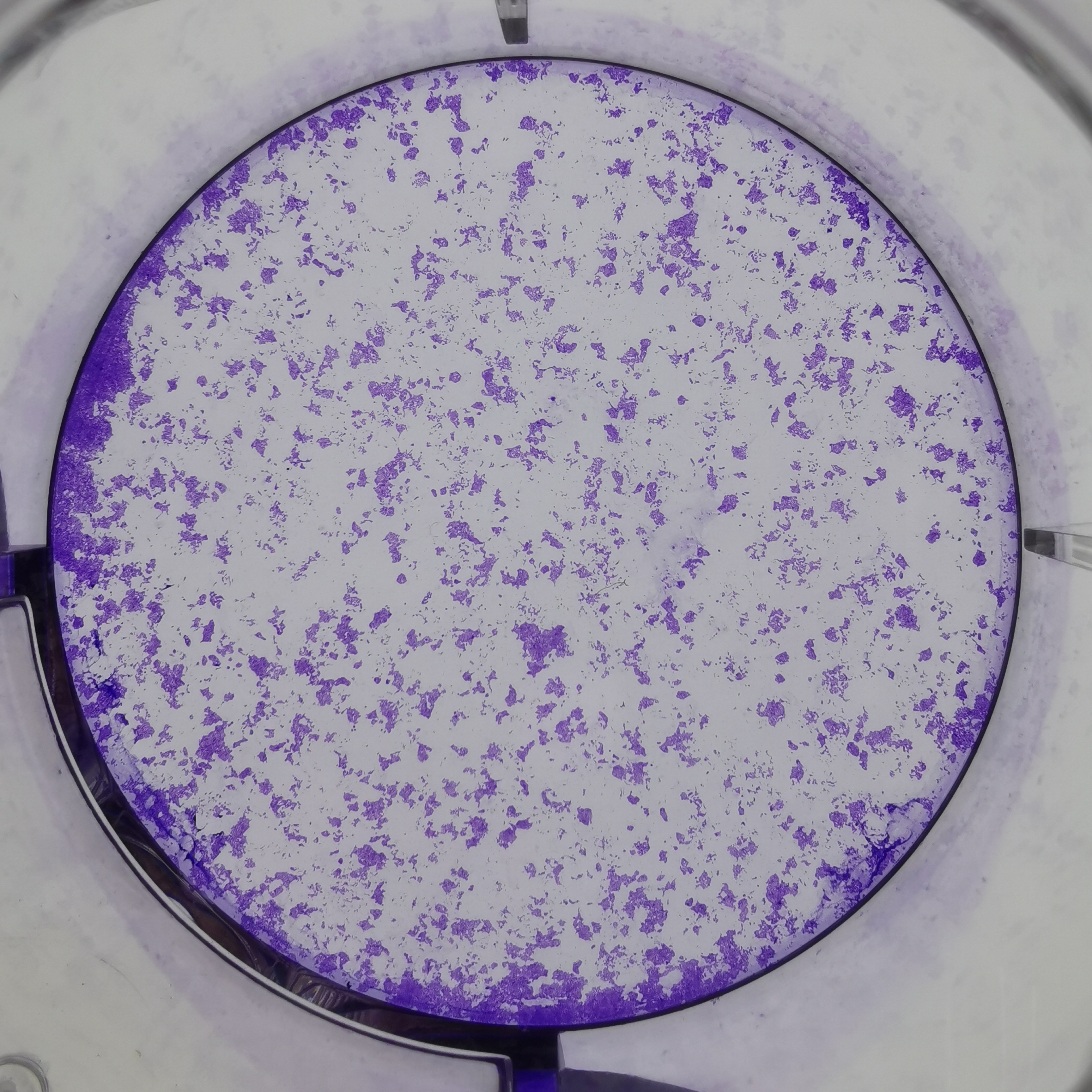

Supplement: Supplementary file 4 — Source data Fig. 2 [file 44319_2024_290_MOESM4_ESM.zip › 2B/Figure 2B-replicate/769-P/769P 2500 SHYY1-4 (3).jpg]

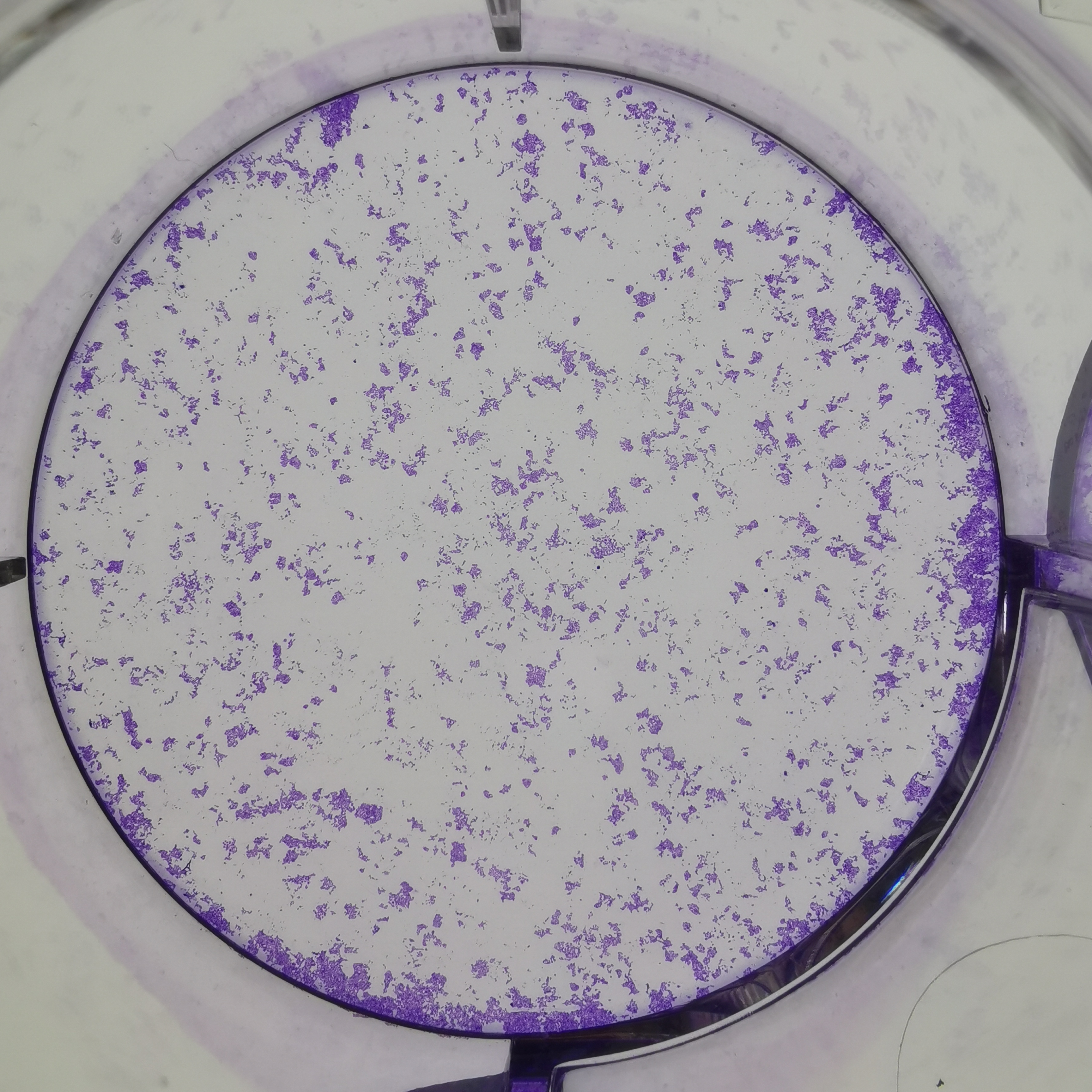

Supplement: Supplementary file 4 — Source data Fig. 2 [file 44319_2024_290_MOESM4_ESM.zip › 2B/Figure 2B-replicate/769-P/769P 2500 SHYY1-5 (1).jpg]

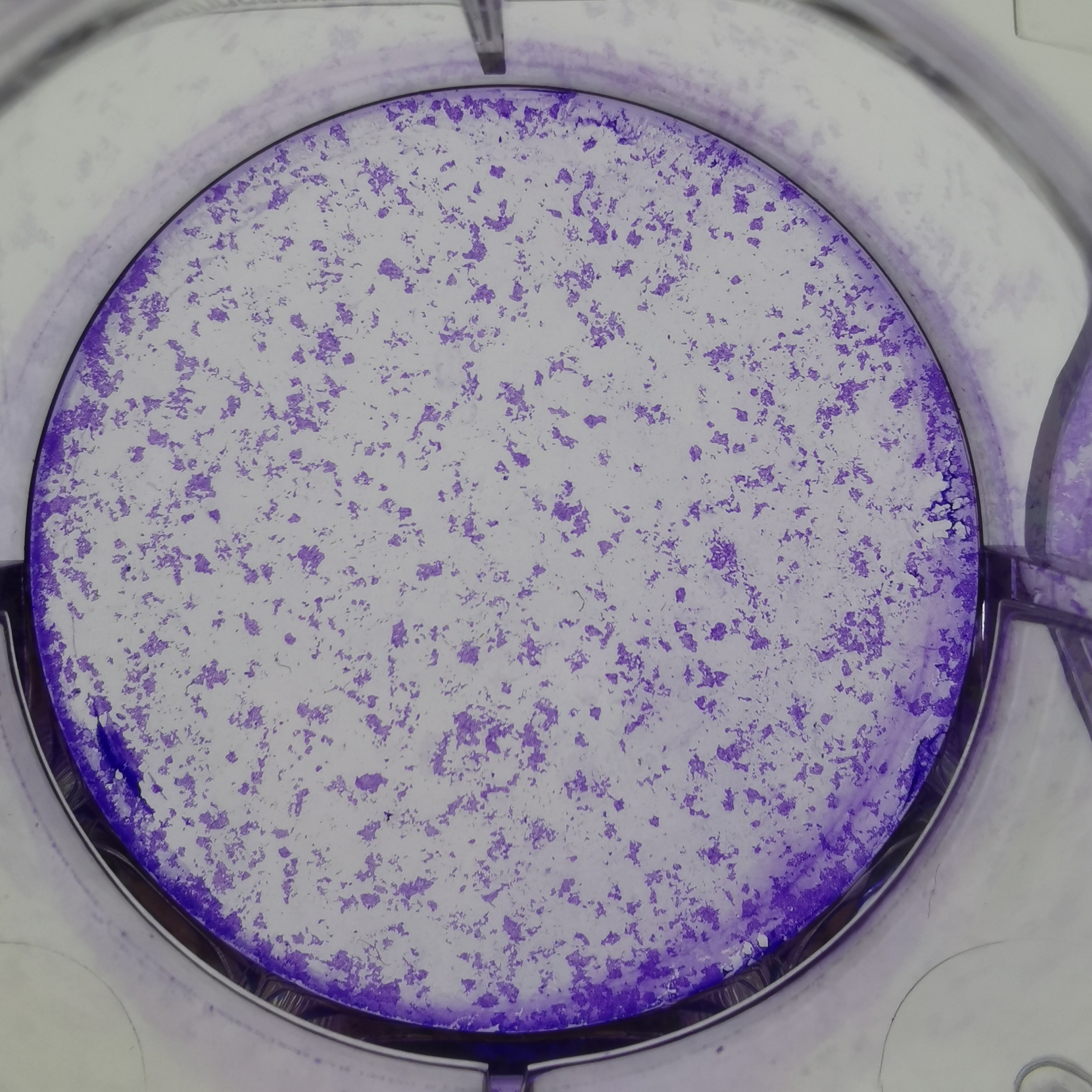

Supplement: Supplementary file 4 — Source data Fig. 2 [file 44319_2024_290_MOESM4_ESM.zip › 2B/Figure 2B-replicate/769-P/769P 2500 SHYY1-5 (2).jpg]

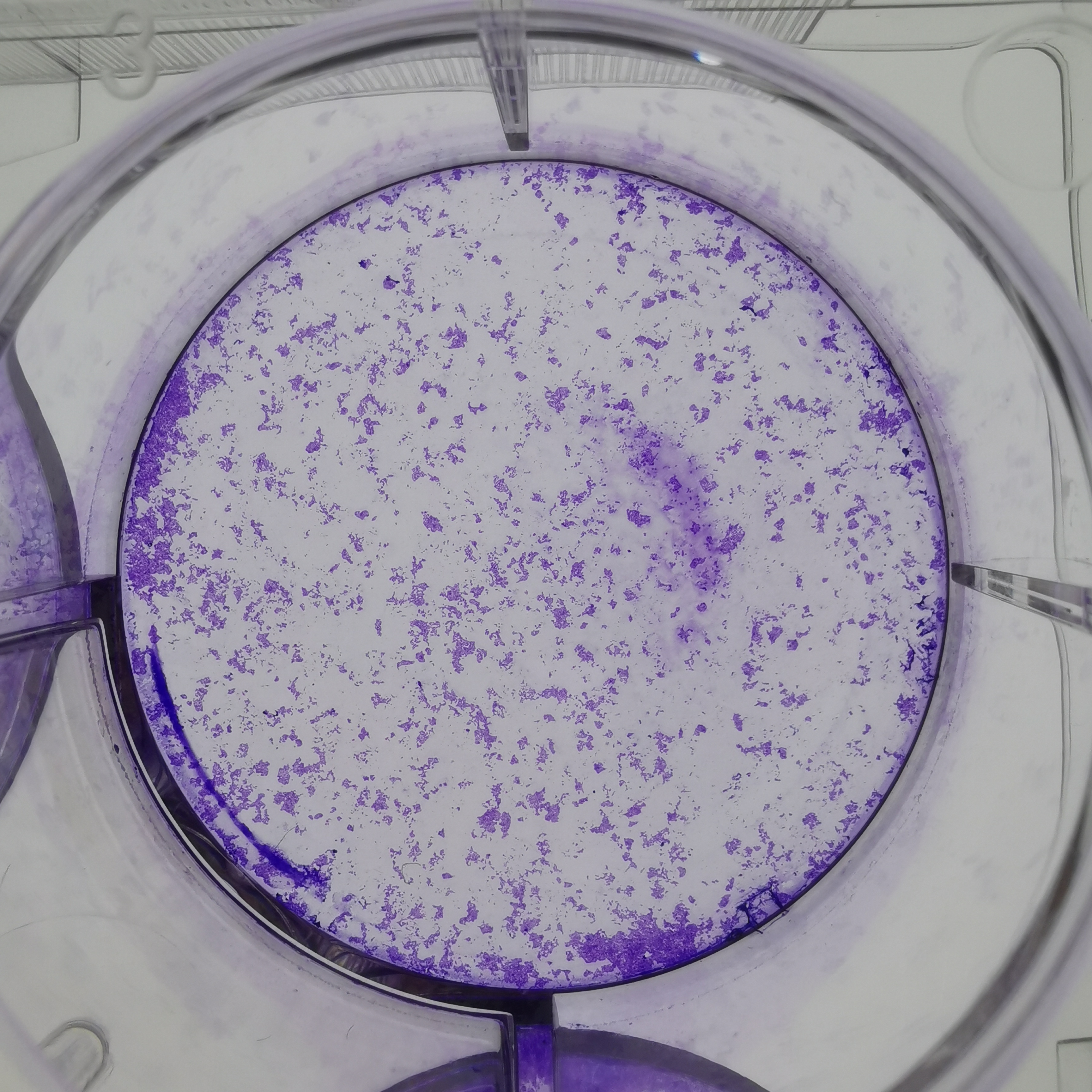

Supplement: Supplementary file 4 — Source data Fig. 2 [file 44319_2024_290_MOESM4_ESM.zip › 2B/Figure 2B-replicate/769-P/769P 2500 SHYY1-5 (3).jpg]

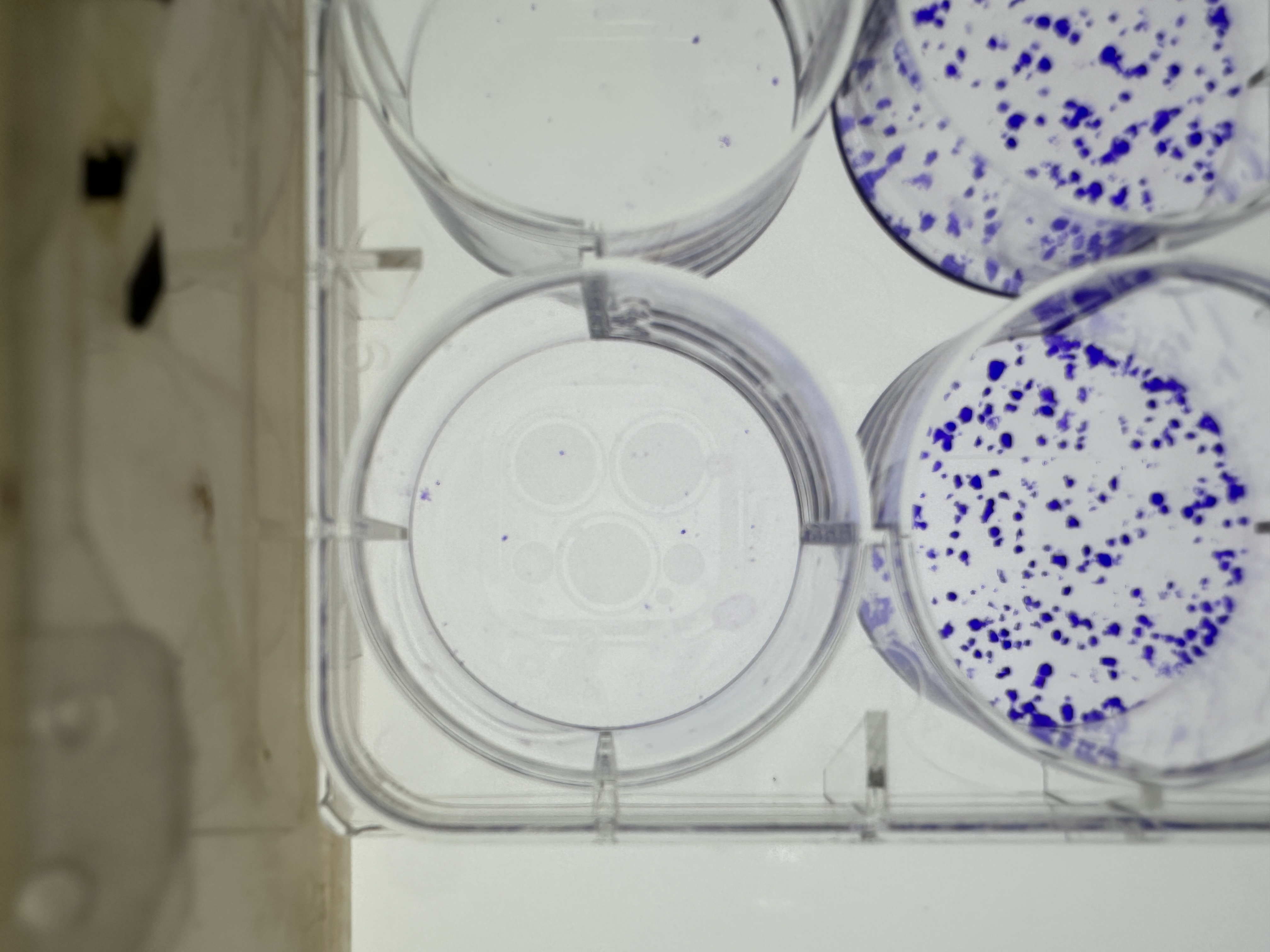

Supplement: Supplementary file 4 — Source data Fig. 2 [file 44319_2024_290_MOESM4_ESM.zip › 2B/Figure 2B-replicate/ACHN/dsh 1 .jpg]

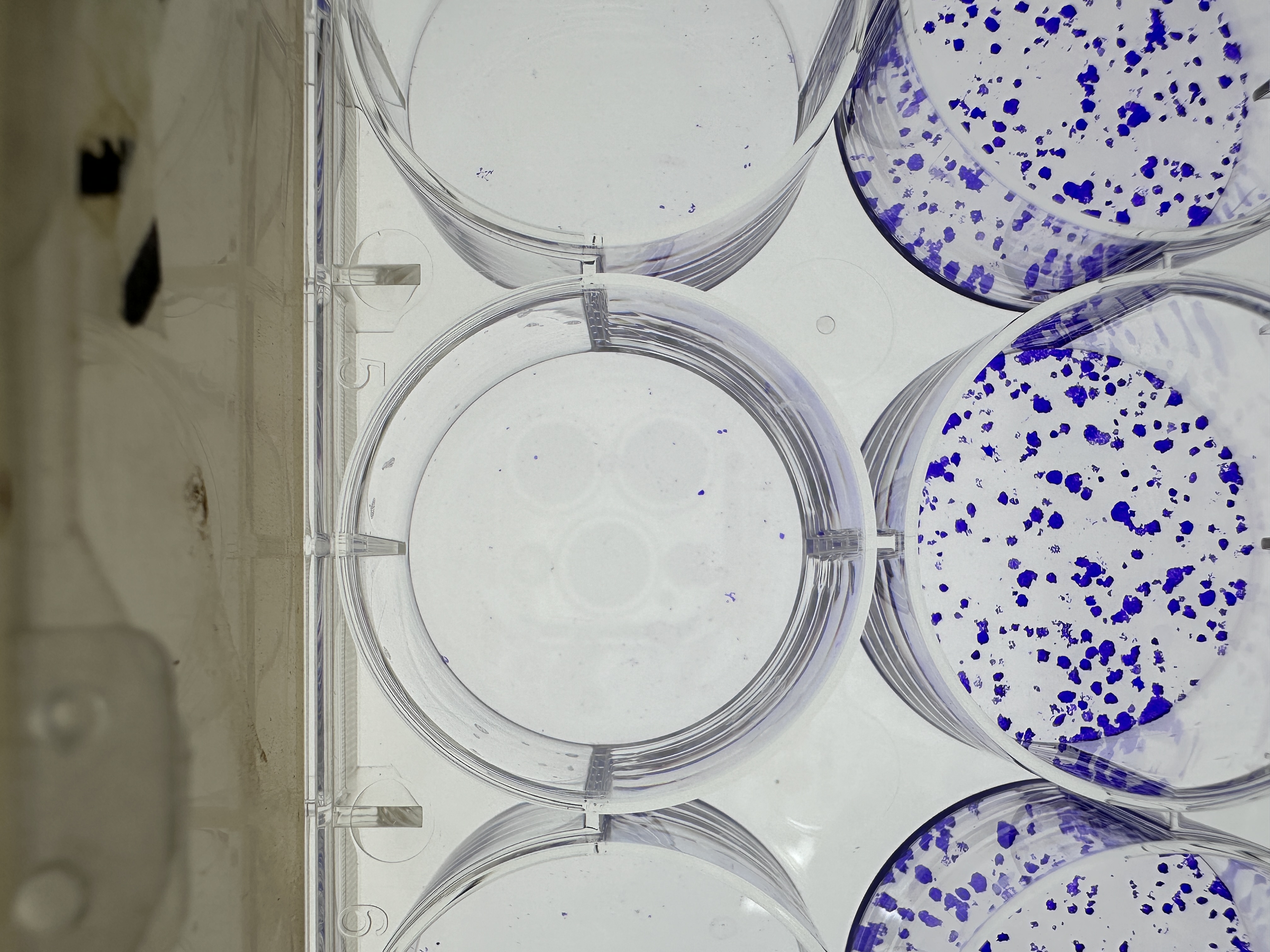

Supplement: Supplementary file 4 — Source data Fig. 2 [file 44319_2024_290_MOESM4_ESM.zip › 2B/Figure 2B-replicate/ACHN/dsh 2 .jpg]

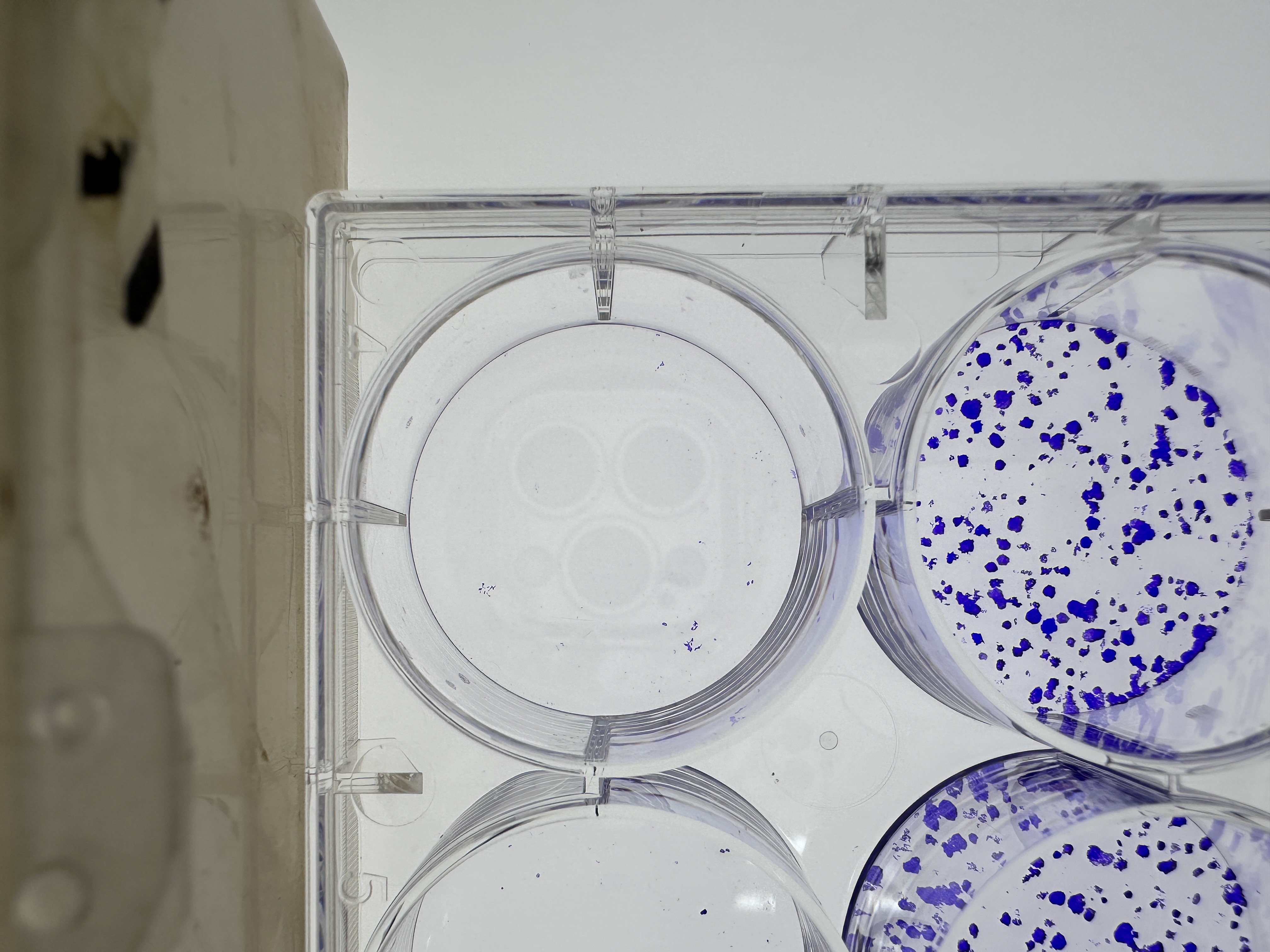

Supplement: Supplementary file 4 — Source data Fig. 2 [file 44319_2024_290_MOESM4_ESM.zip › 2B/Figure 2B-replicate/ACHN/dsh 3 .jpg]

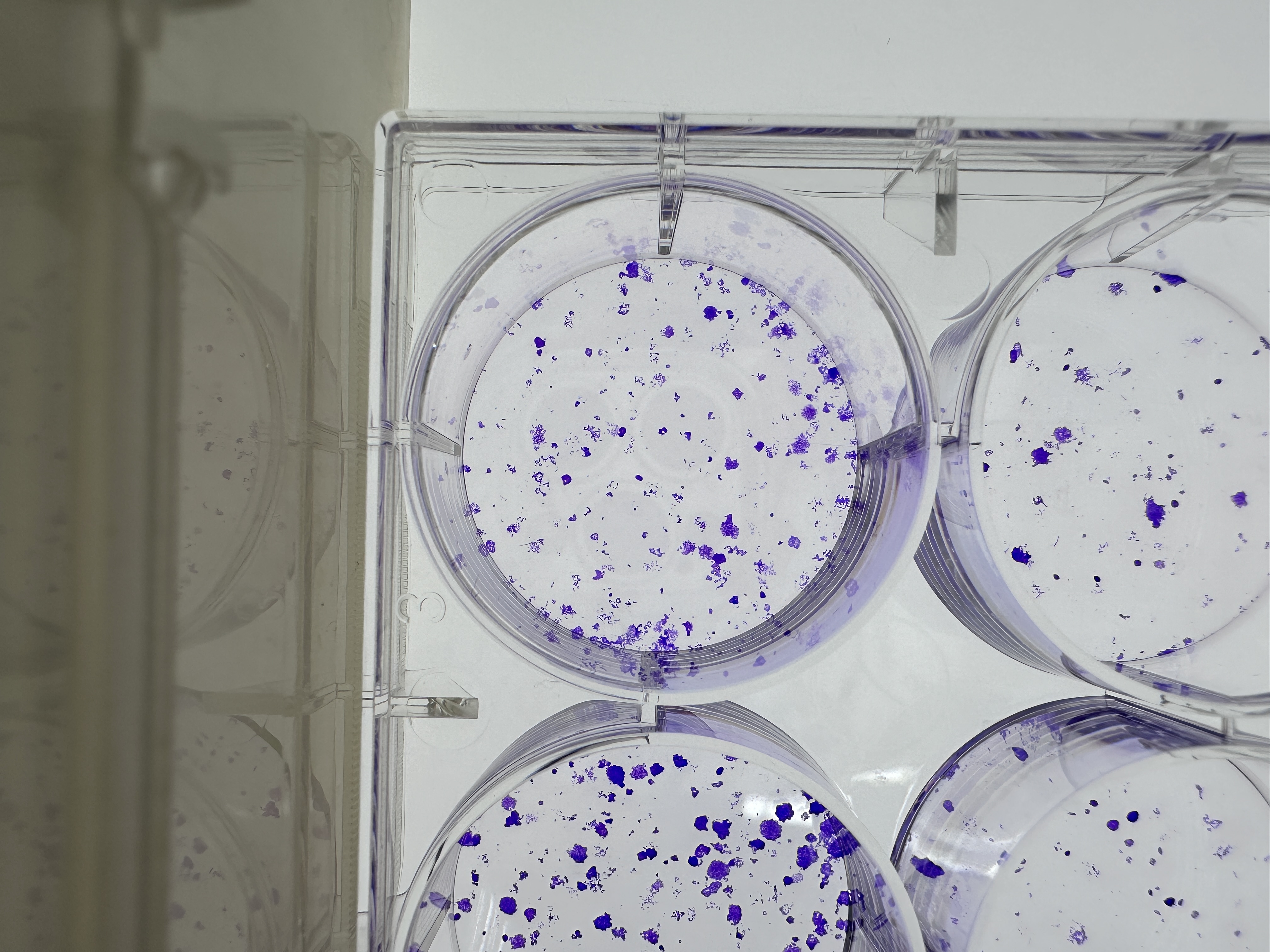

Supplement: Supplementary file 4 — Source data Fig. 2 [file 44319_2024_290_MOESM4_ESM.zip › 2B/Figure 2B-replicate/ACHN/dsh+5c-1.jpg]

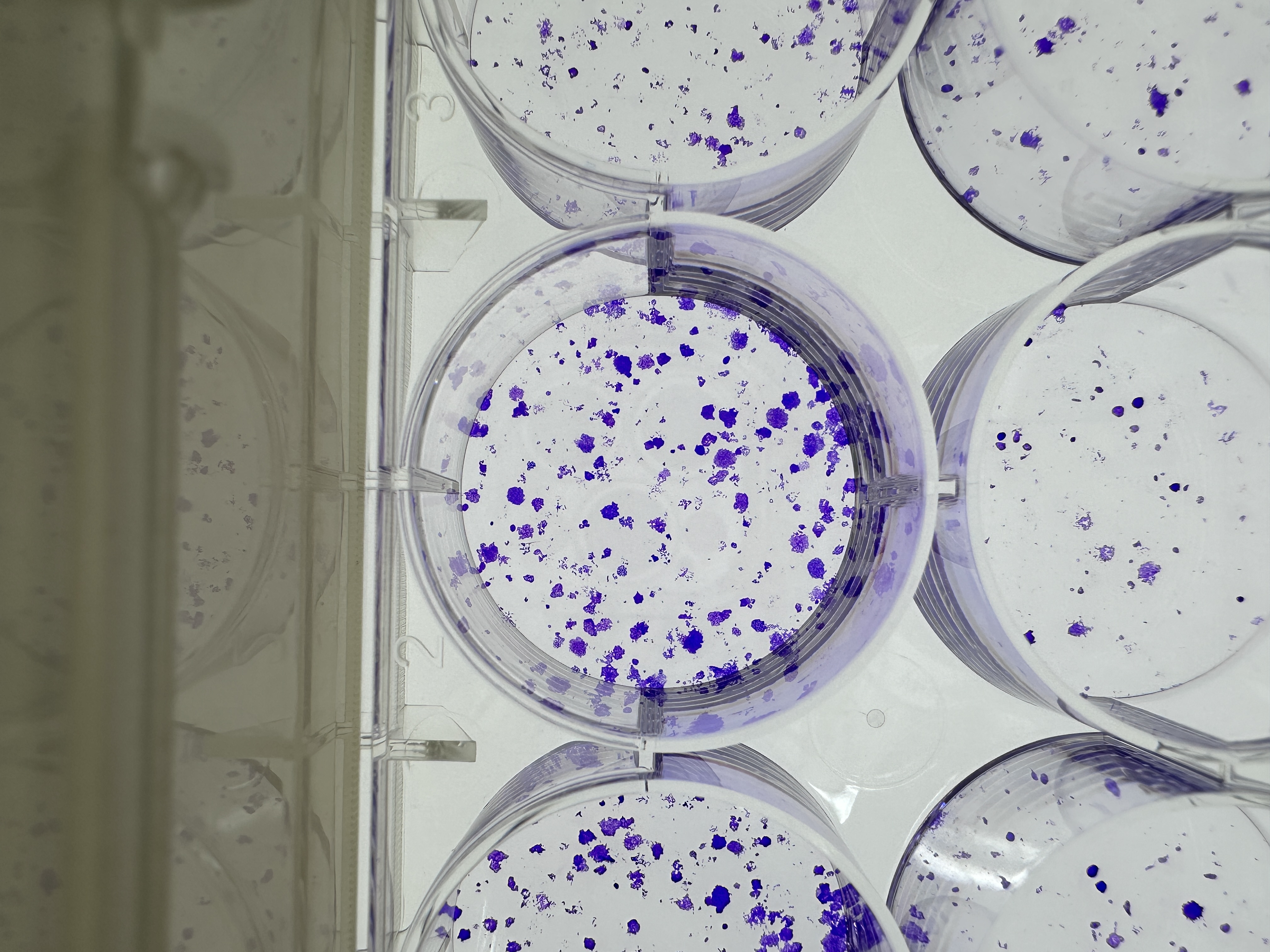

Supplement: Supplementary file 4 — Source data Fig. 2 [file 44319_2024_290_MOESM4_ESM.zip › 2B/Figure 2B-replicate/ACHN/dsh+5c-2.jpg]

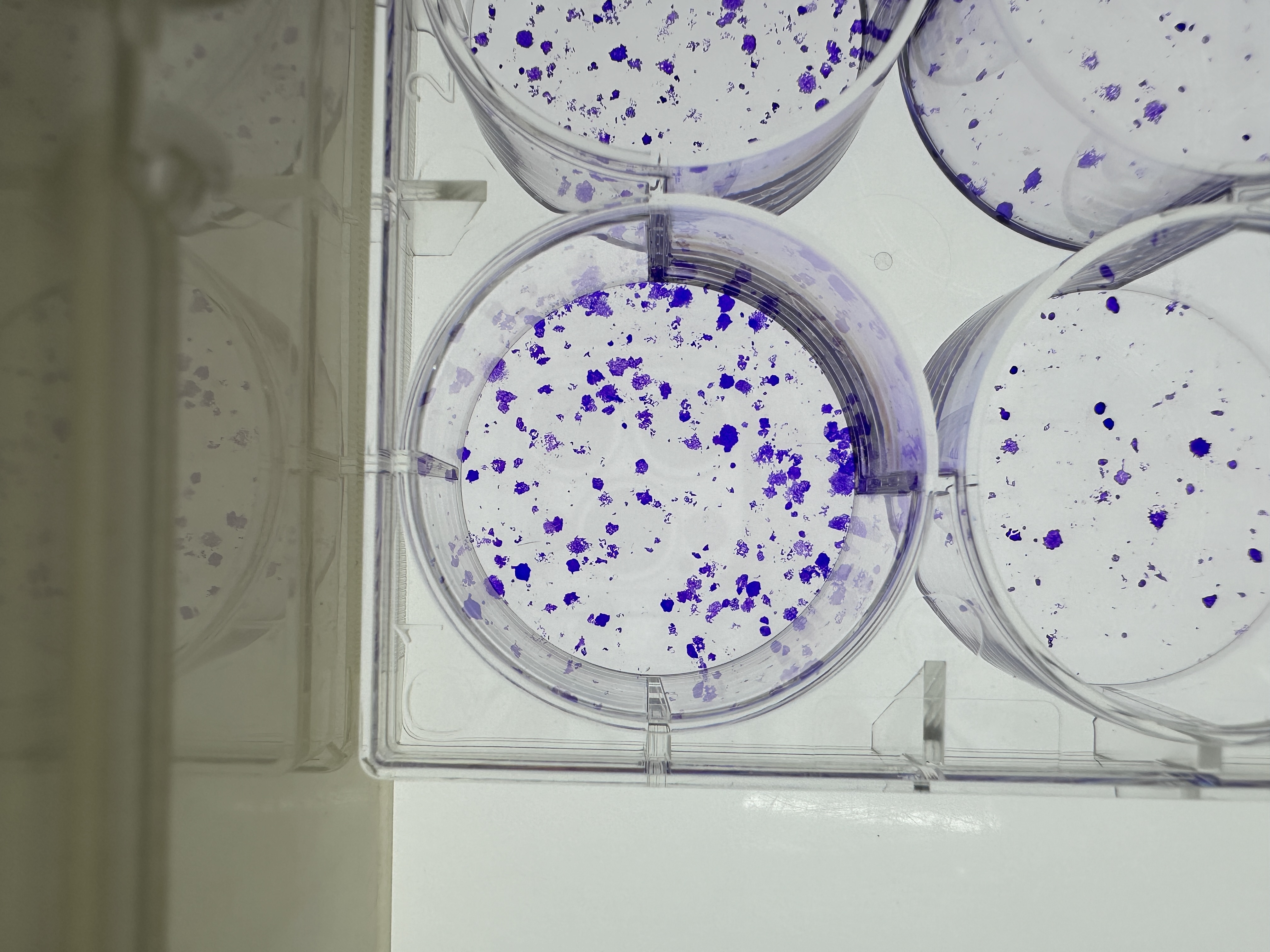

Supplement: Supplementary file 4 — Source data Fig. 2 [file 44319_2024_290_MOESM4_ESM.zip › 2B/Figure 2B-replicate/ACHN/dsh+5c-3.jpg]

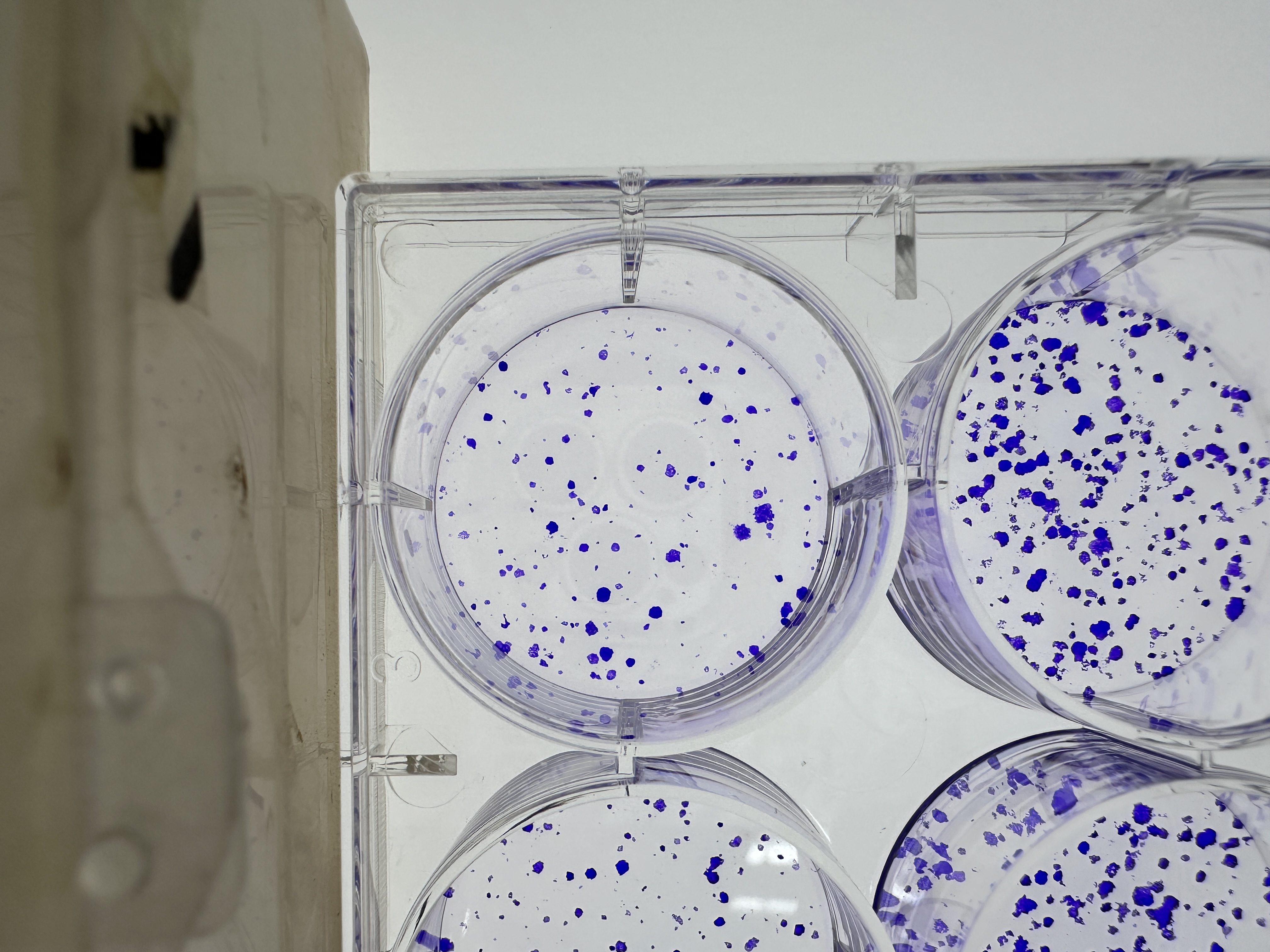

Supplement: Supplementary file 4 — Source data Fig. 2 [file 44319_2024_290_MOESM4_ESM.zip › 2B/Figure 2B-replicate/ACHN/dsh+H514A-1.jpg]

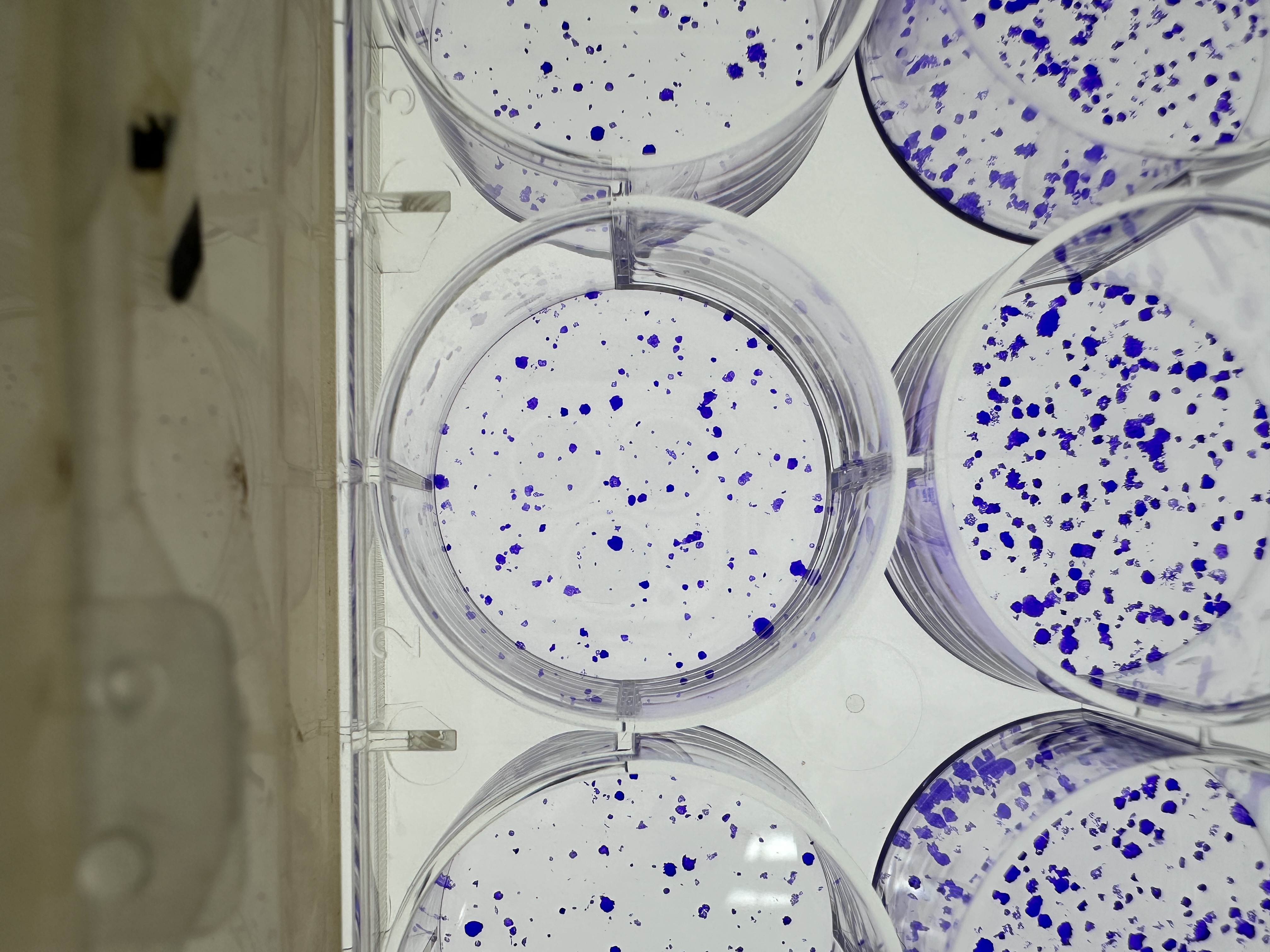

Supplement: Supplementary file 4 — Source data Fig. 2 [file 44319_2024_290_MOESM4_ESM.zip › 2B/Figure 2B-replicate/ACHN/dsh+H514A-2.jpg]

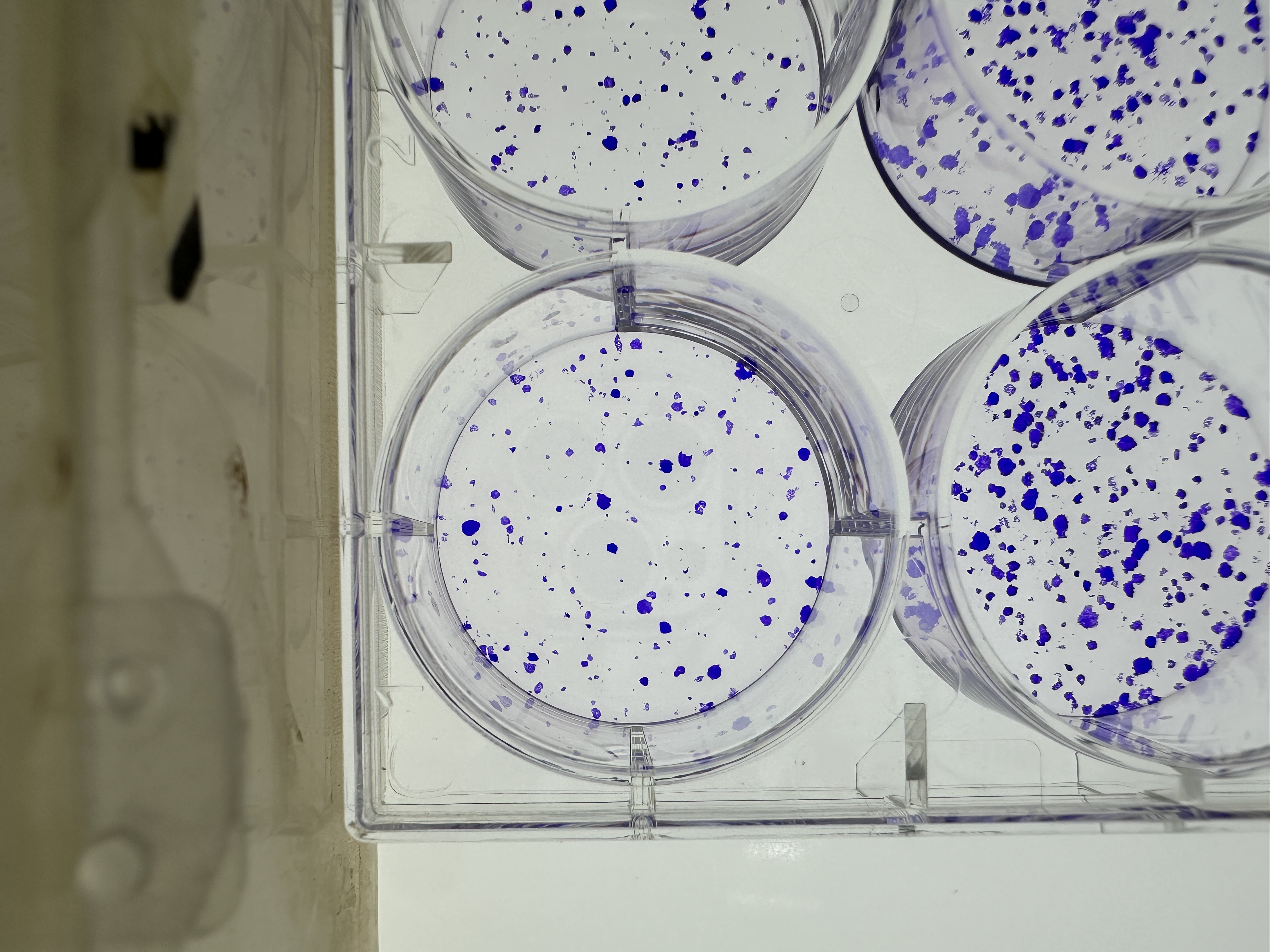

Supplement: Supplementary file 4 — Source data Fig. 2 [file 44319_2024_290_MOESM4_ESM.zip › 2B/Figure 2B-replicate/ACHN/dsh+H514A-3.jpg]

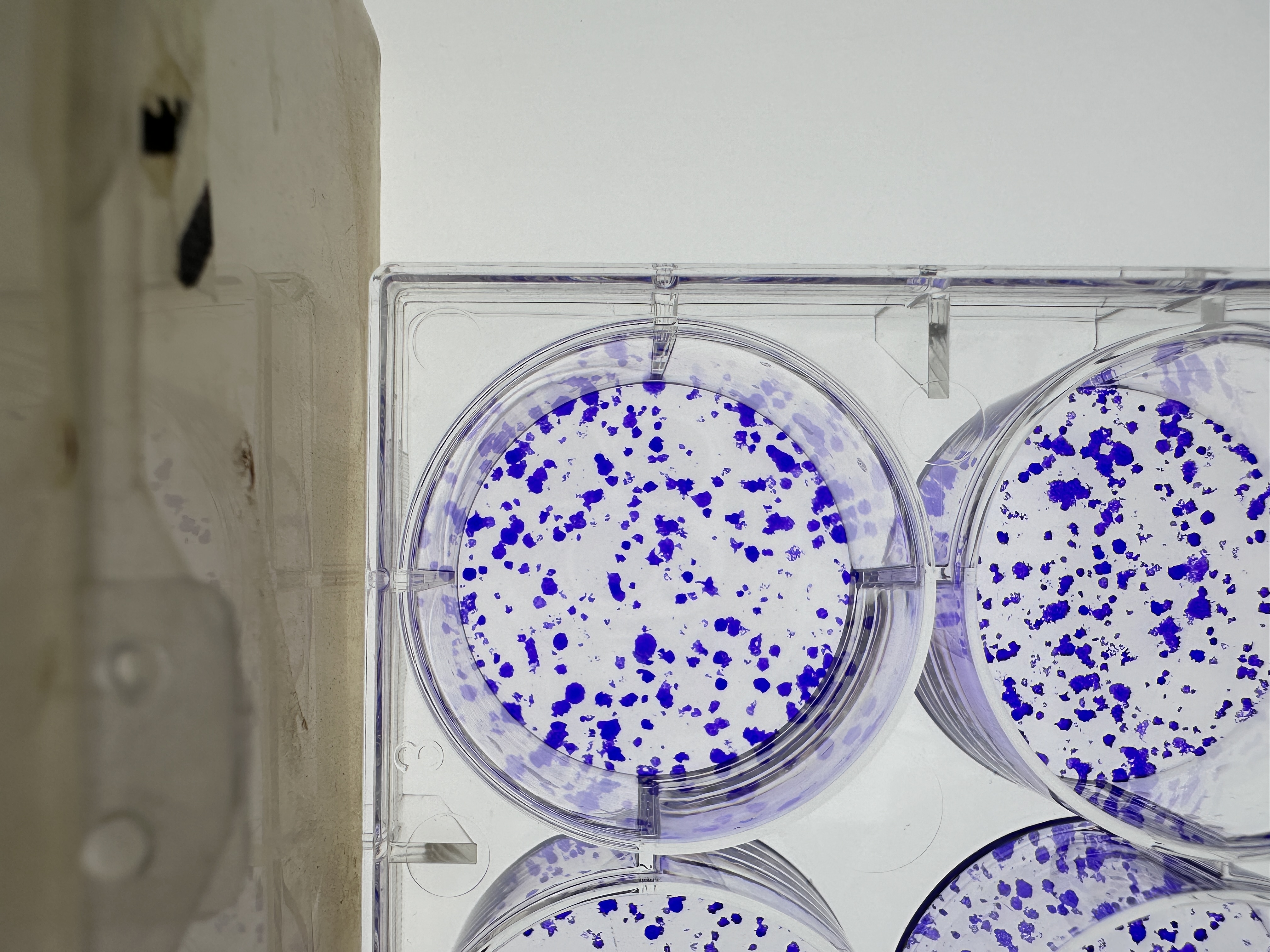

Supplement: Supplementary file 4 — Source data Fig. 2 [file 44319_2024_290_MOESM4_ESM.zip › 2B/Figure 2B-replicate/ACHN/shCtrl-1.jpg]

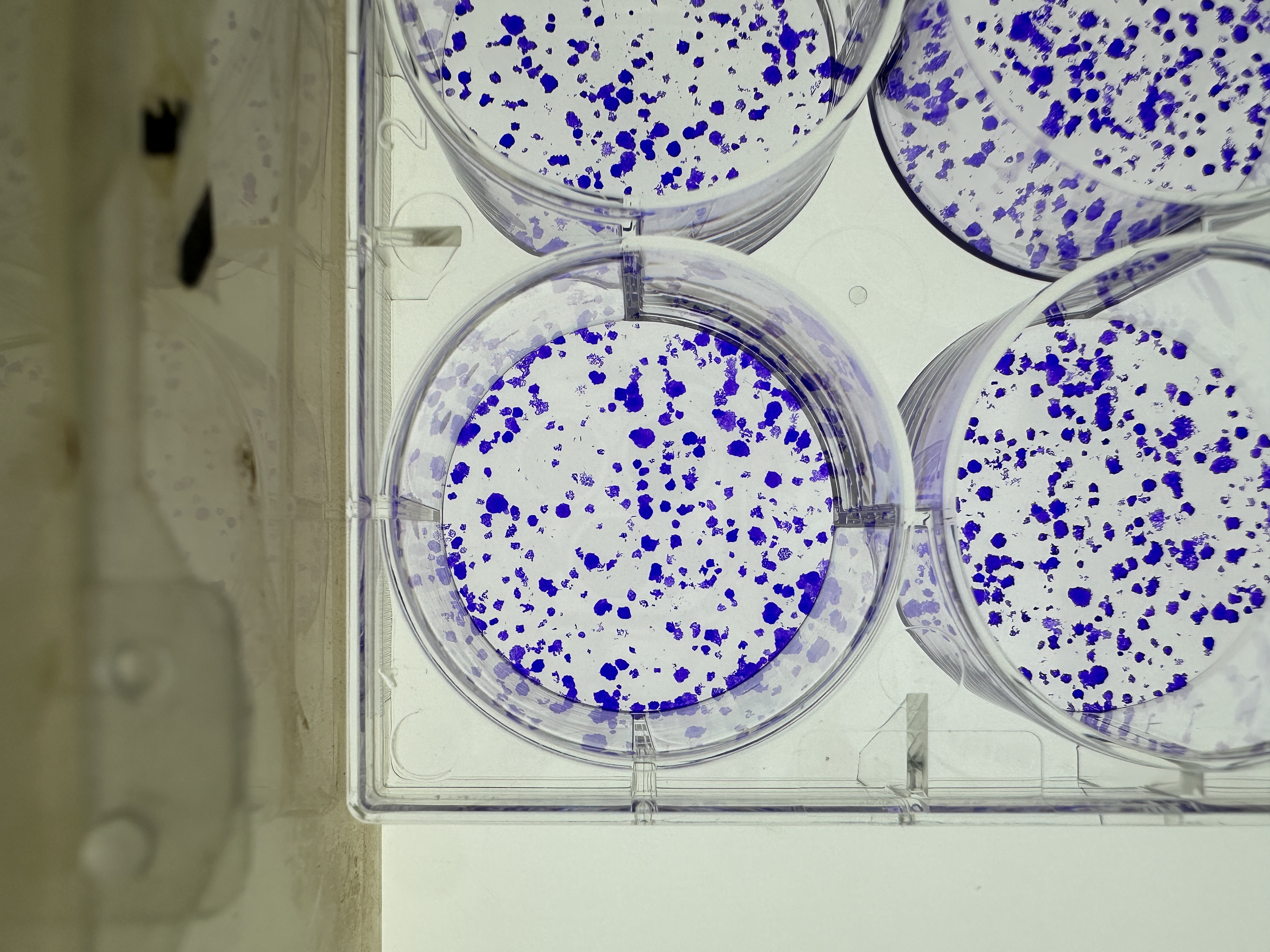

Supplement: Supplementary file 4 — Source data Fig. 2 [file 44319_2024_290_MOESM4_ESM.zip › 2B/Figure 2B-replicate/ACHN/shCtrl-2.jpg]

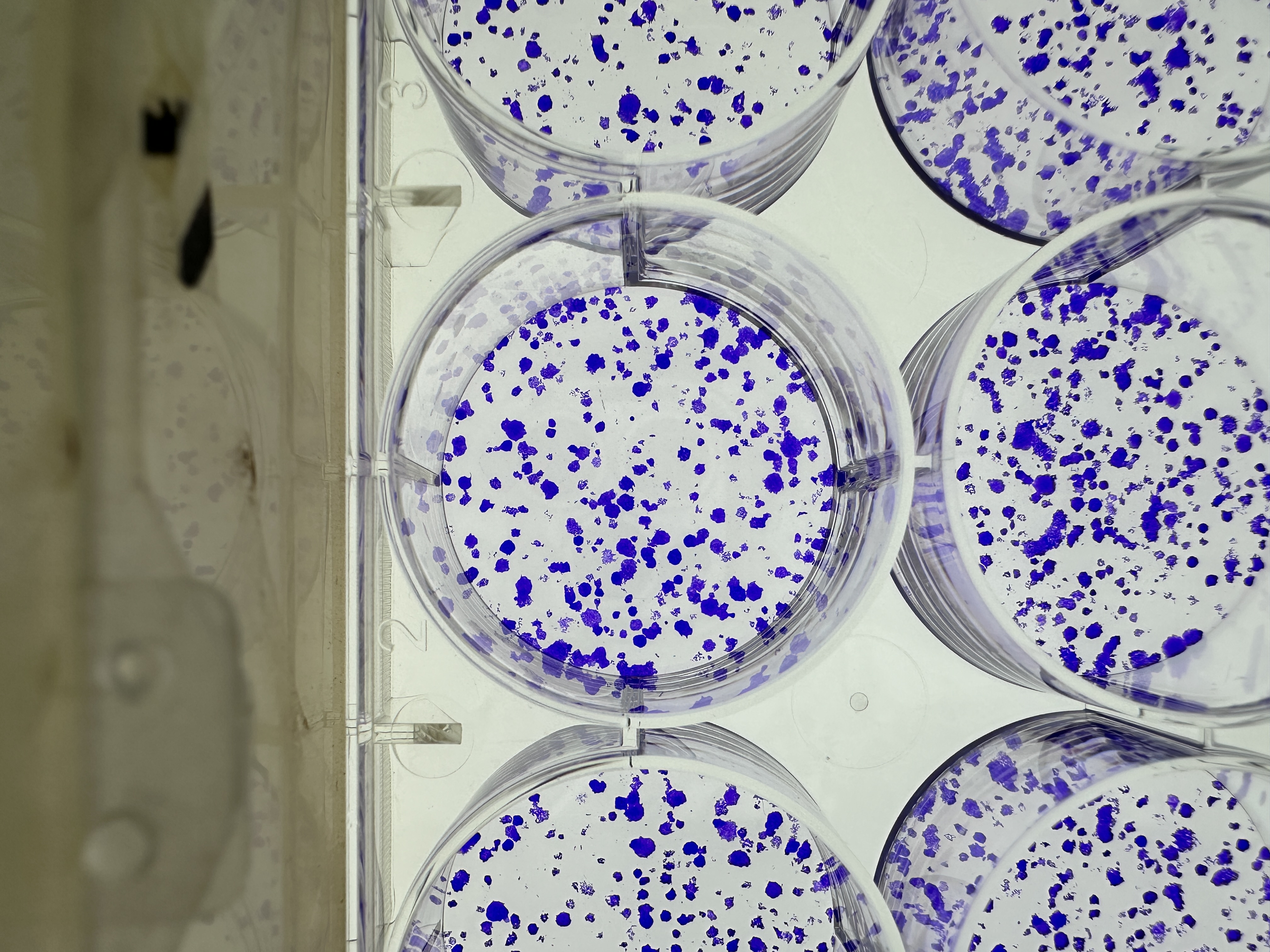

Supplement: Supplementary file 4 — Source data Fig. 2 [file 44319_2024_290_MOESM4_ESM.zip › 2B/Figure 2B-replicate/ACHN/shCtrl-3.jpg]

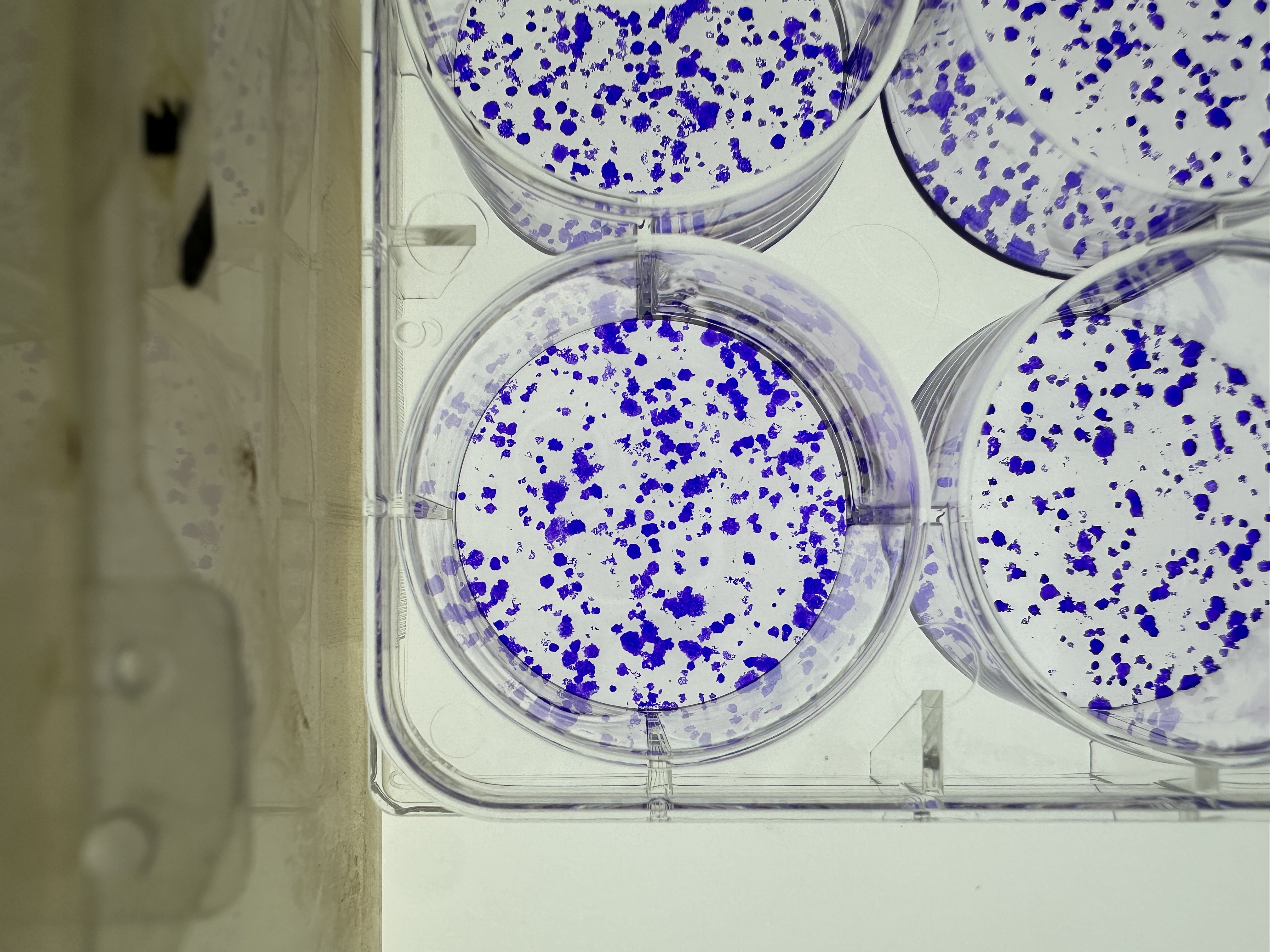

Supplement: Supplementary file 4 — Source data Fig. 2 [file 44319_2024_290_MOESM4_ESM.zip › 2B/Figure 2B-replicate/ACHN/shKDM5C-1.jpg]

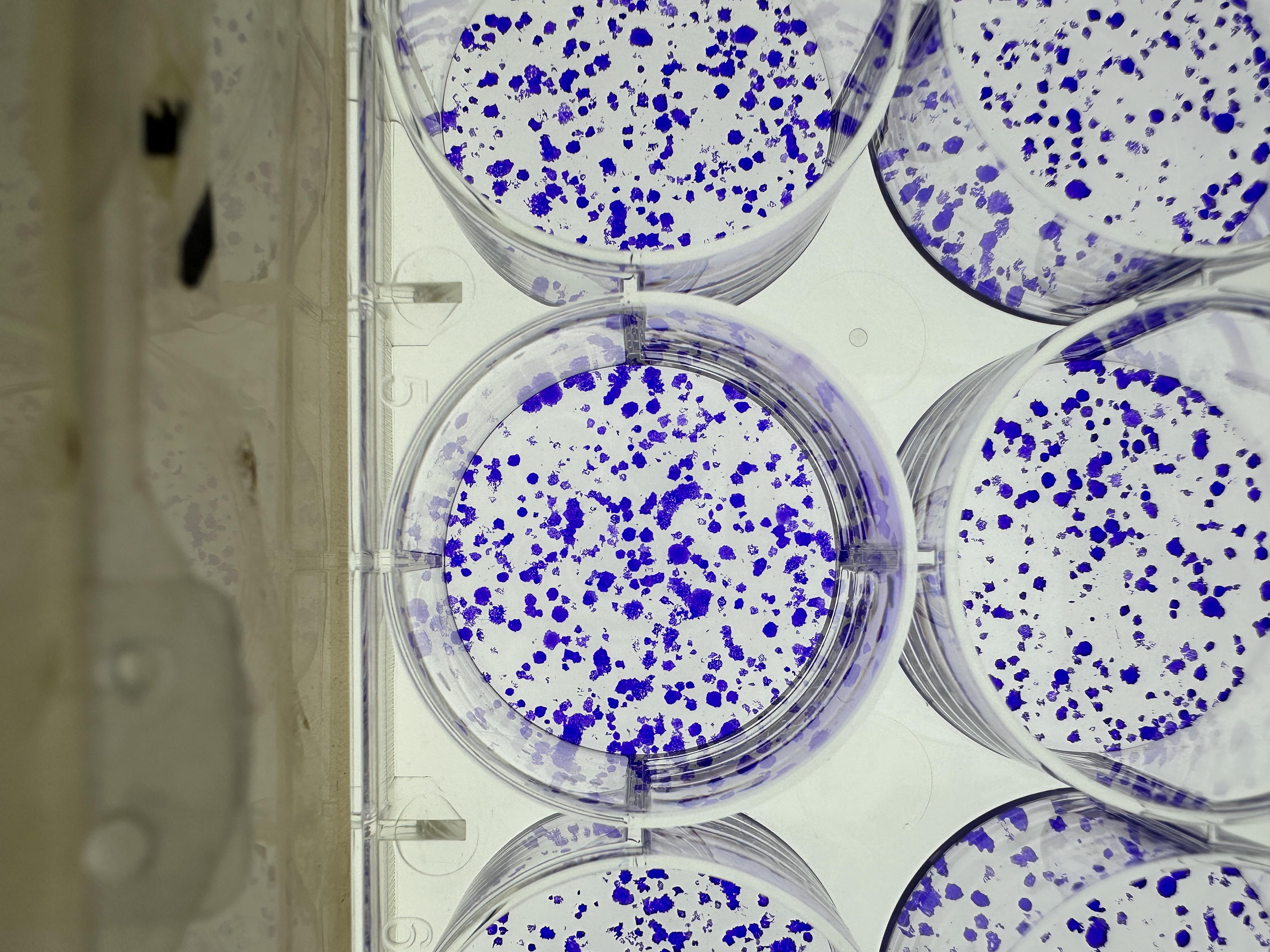

Supplement: Supplementary file 4 — Source data Fig. 2 [file 44319_2024_290_MOESM4_ESM.zip › 2B/Figure 2B-replicate/ACHN/shKDM5C-2.jpg]

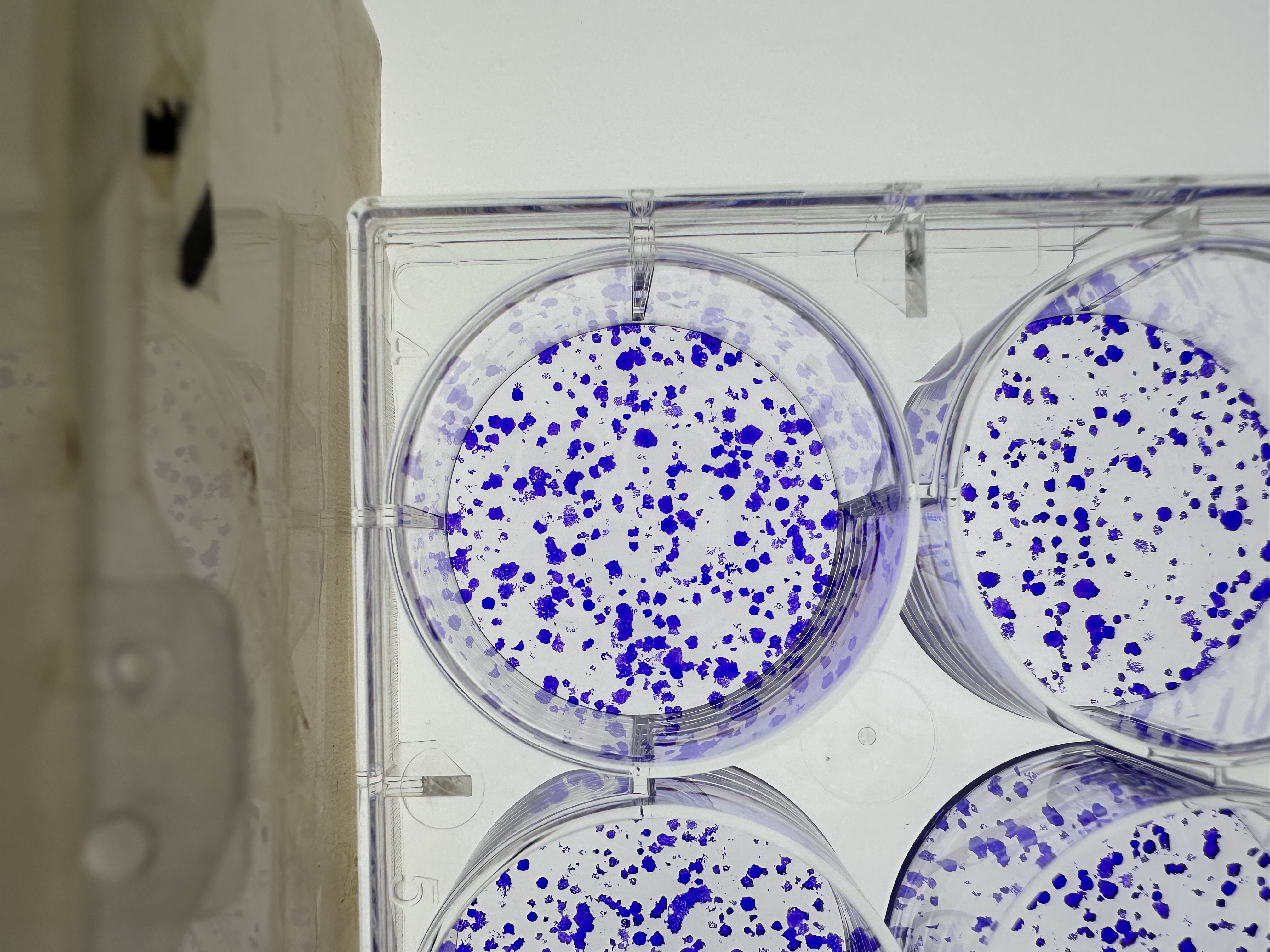

Supplement: Supplementary file 4 — Source data Fig. 2 [file 44319_2024_290_MOESM4_ESM.zip › 2B/Figure 2B-replicate/ACHN/shKDM5C-3.jpg]

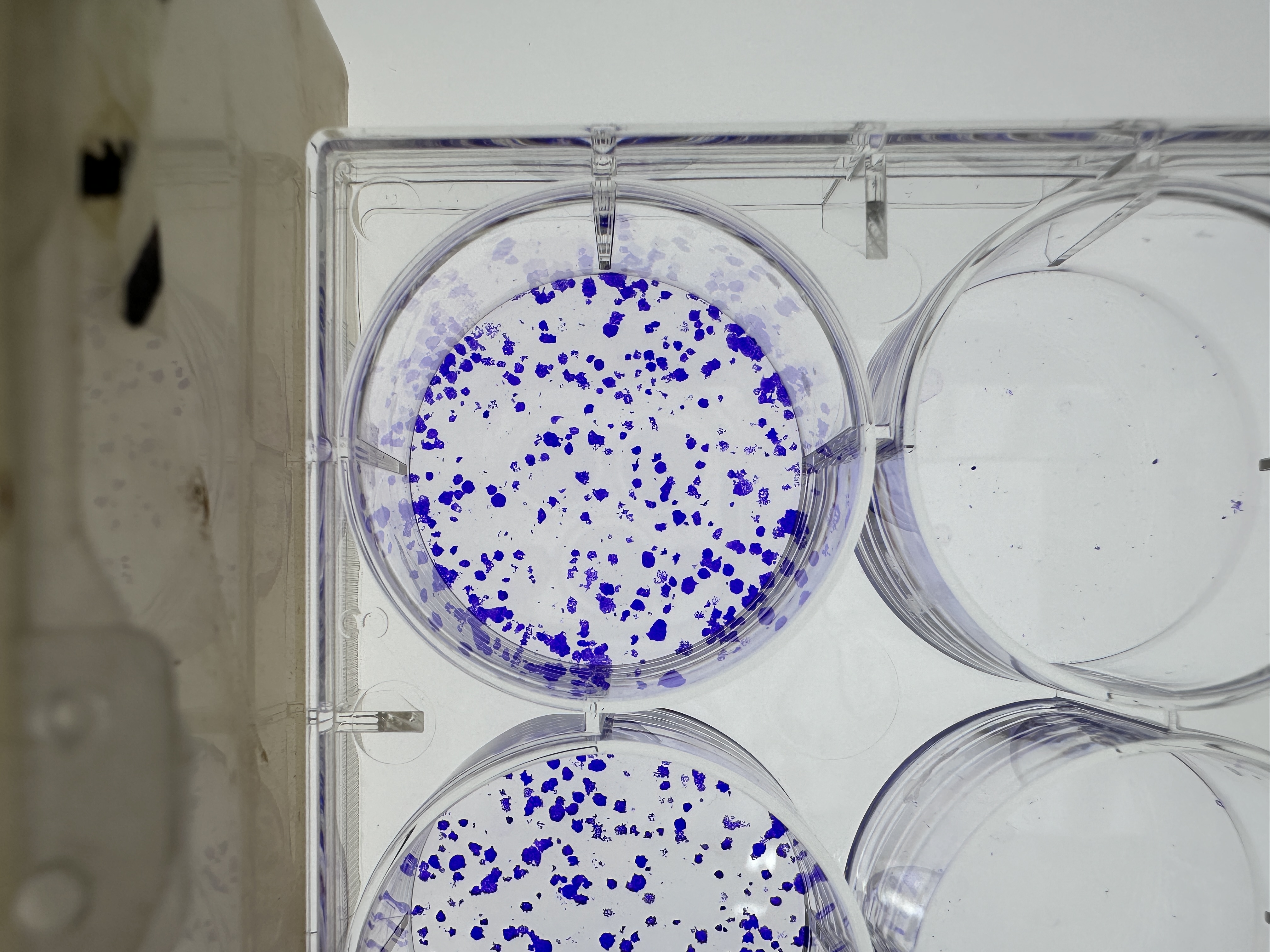

Supplement: Supplementary file 4 — Source data Fig. 2 [file 44319_2024_290_MOESM4_ESM.zip › 2B/Figure 2B-replicate/ACHN/shYY1-1-1.jpg]

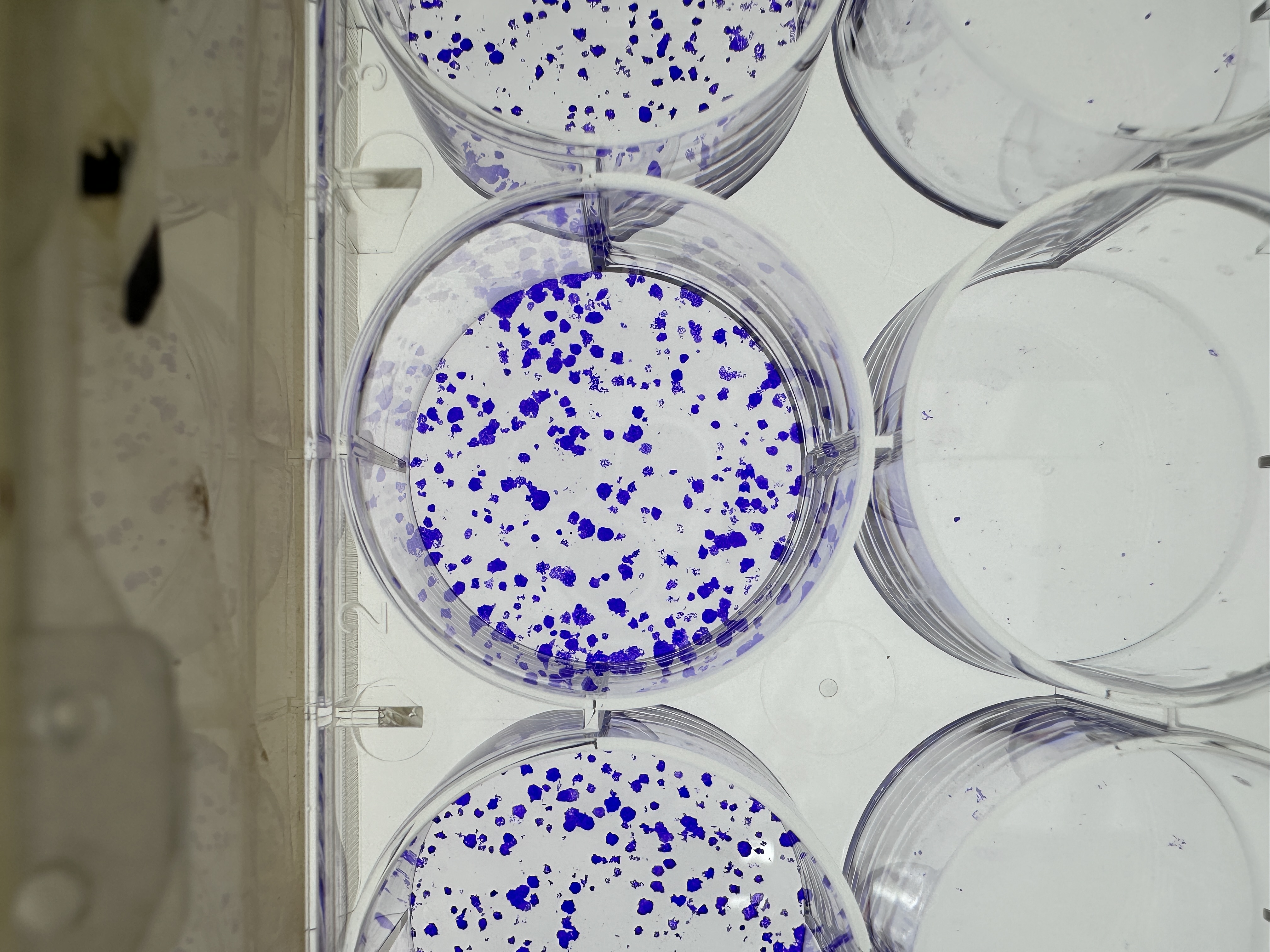

Supplement: Supplementary file 4 — Source data Fig. 2 [file 44319_2024_290_MOESM4_ESM.zip › 2B/Figure 2B-replicate/ACHN/shYY1-1-2.jpg]

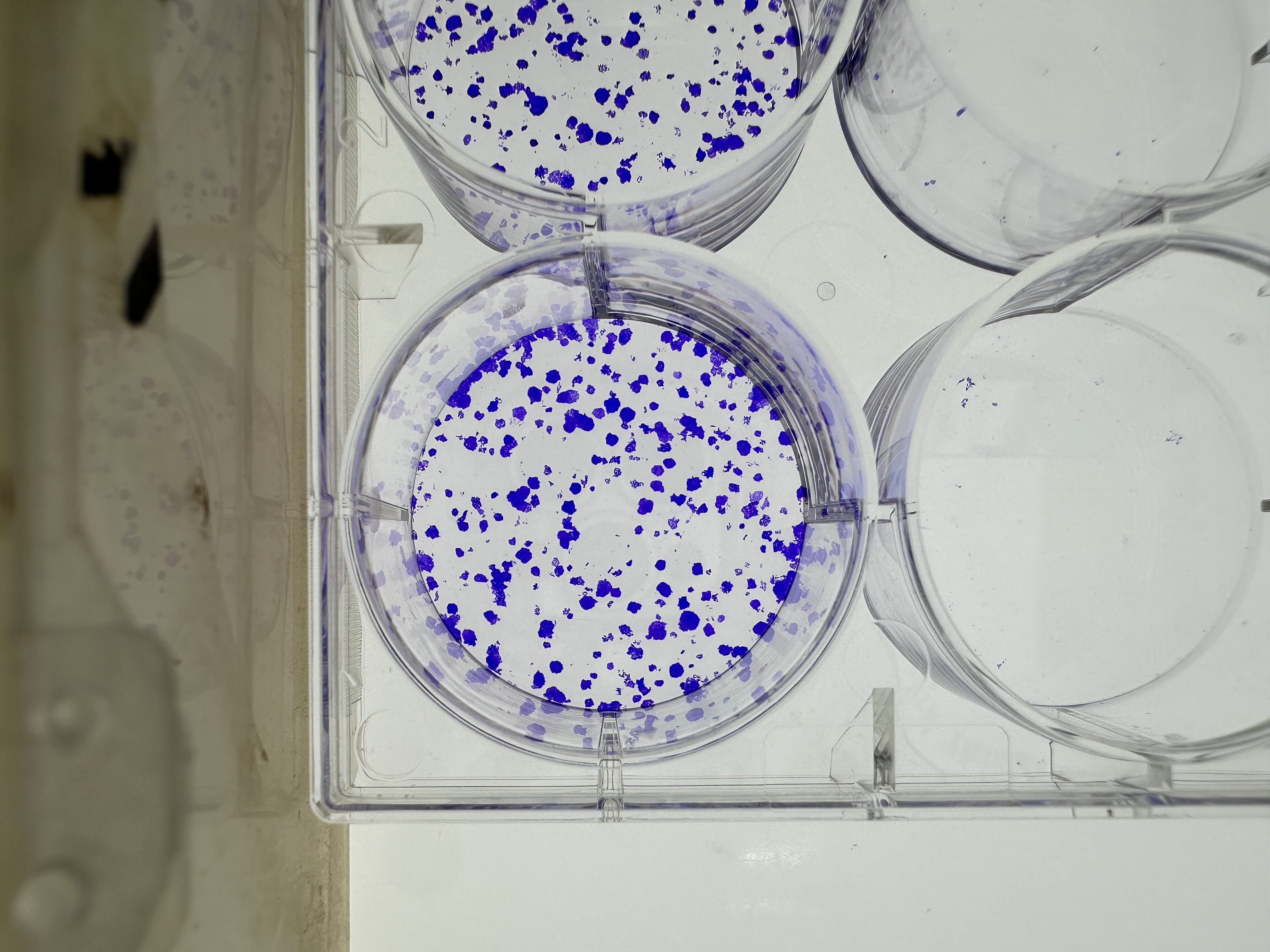

Supplement: Supplementary file 4 — Source data Fig. 2 [file 44319_2024_290_MOESM4_ESM.zip › 2B/Figure 2B-replicate/ACHN/shYY1-1-3.jpg]

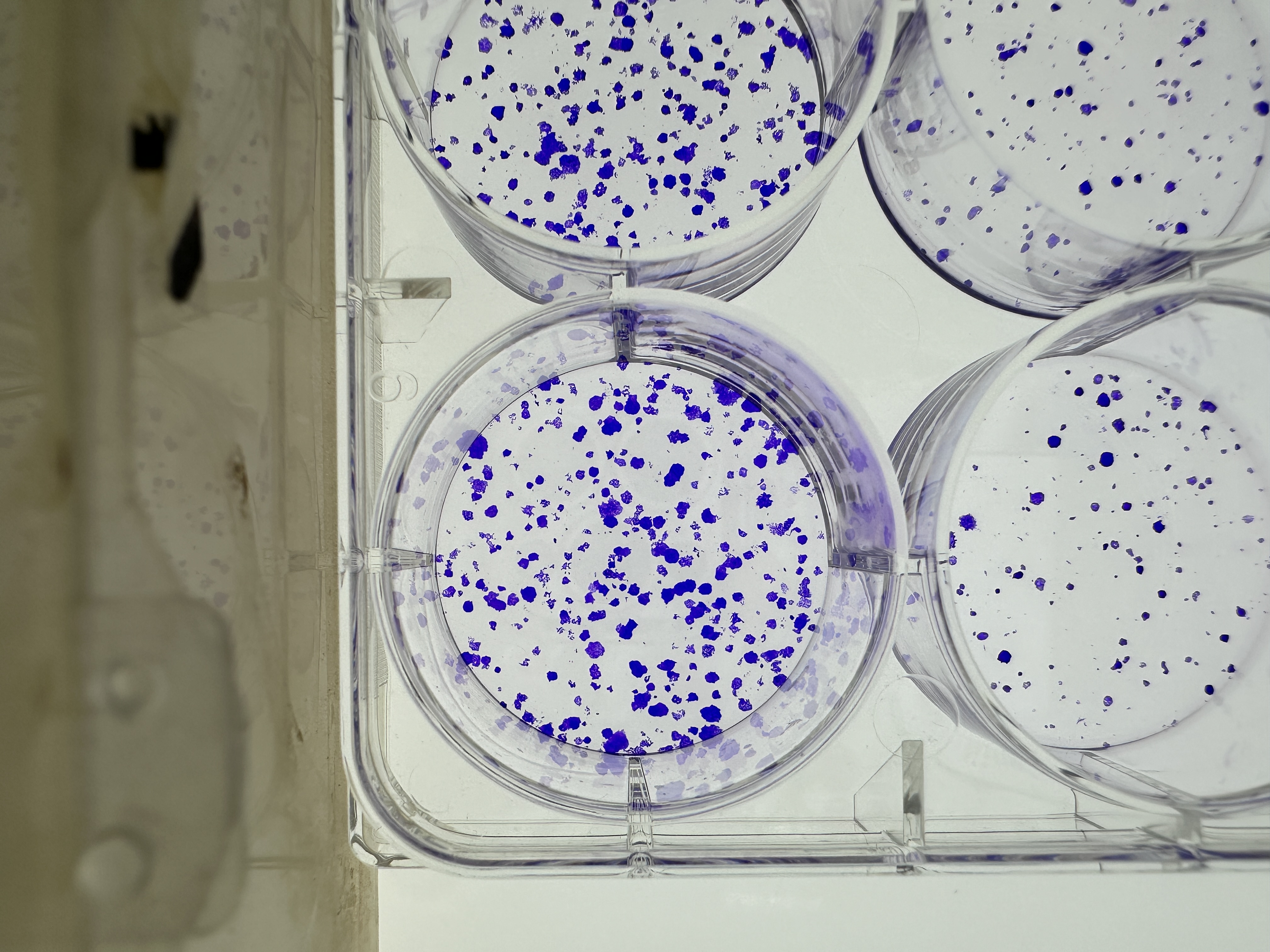

Supplement: Supplementary file 4 — Source data Fig. 2 [file 44319_2024_290_MOESM4_ESM.zip › 2B/Figure 2B-replicate/ACHN/shYY1-2-1.jpg]

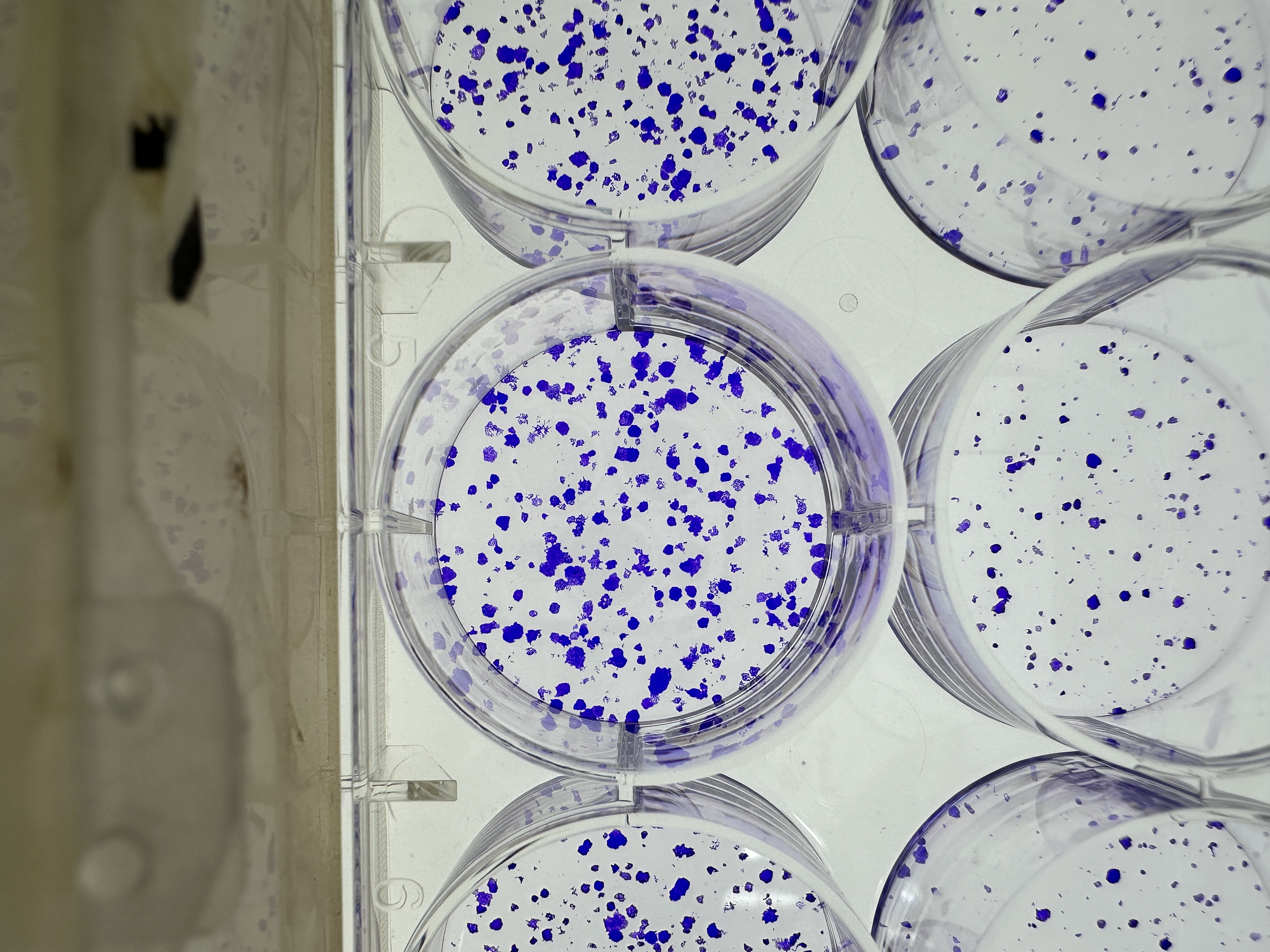

Supplement: Supplementary file 4 — Source data Fig. 2 [file 44319_2024_290_MOESM4_ESM.zip › 2B/Figure 2B-replicate/ACHN/shYY1-2-2.jpg]

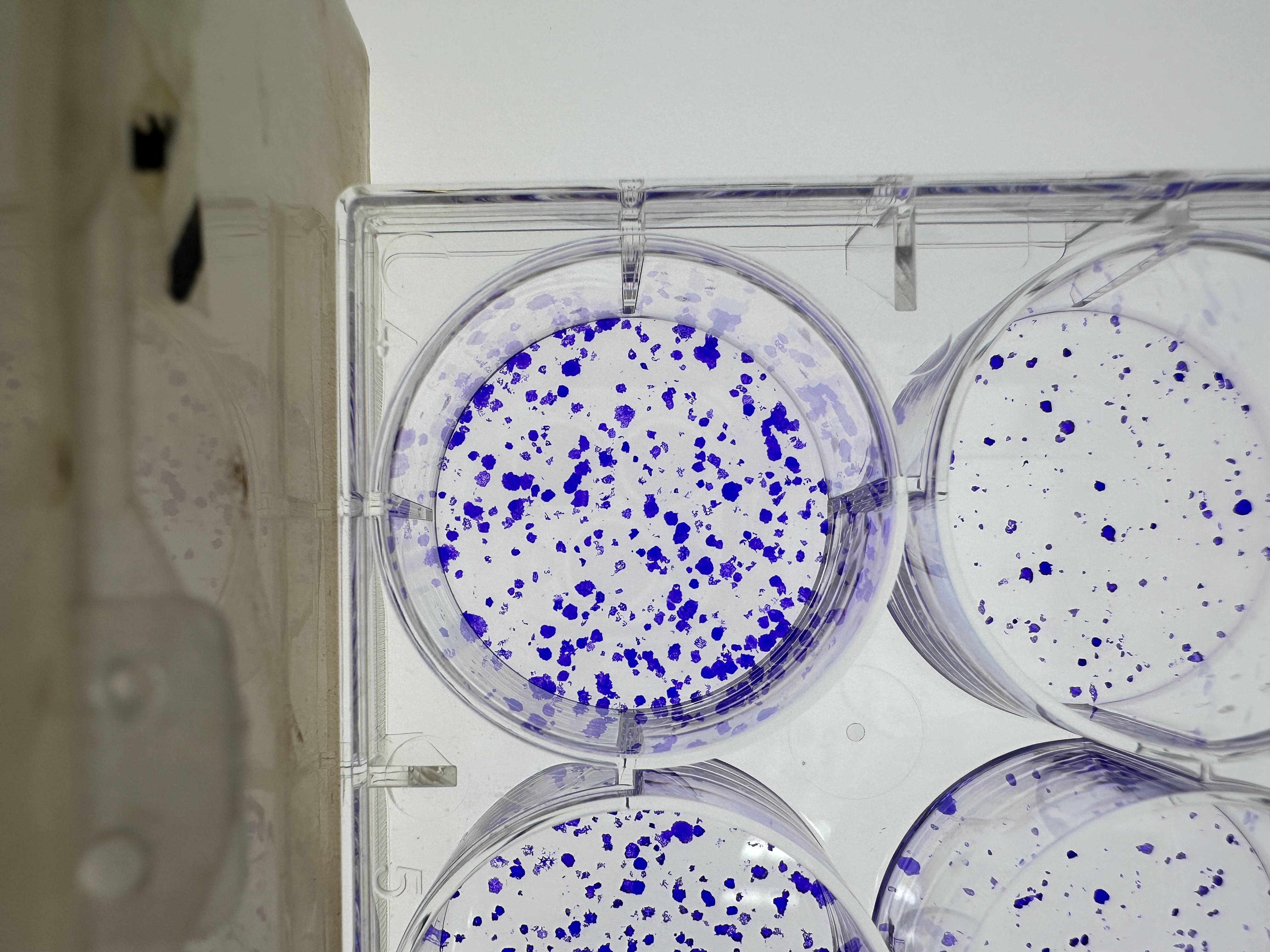

Supplement: Supplementary file 4 — Source data Fig. 2 [file 44319_2024_290_MOESM4_ESM.zip › 2B/Figure 2B-replicate/ACHN/shYY1-2-3.jpg]

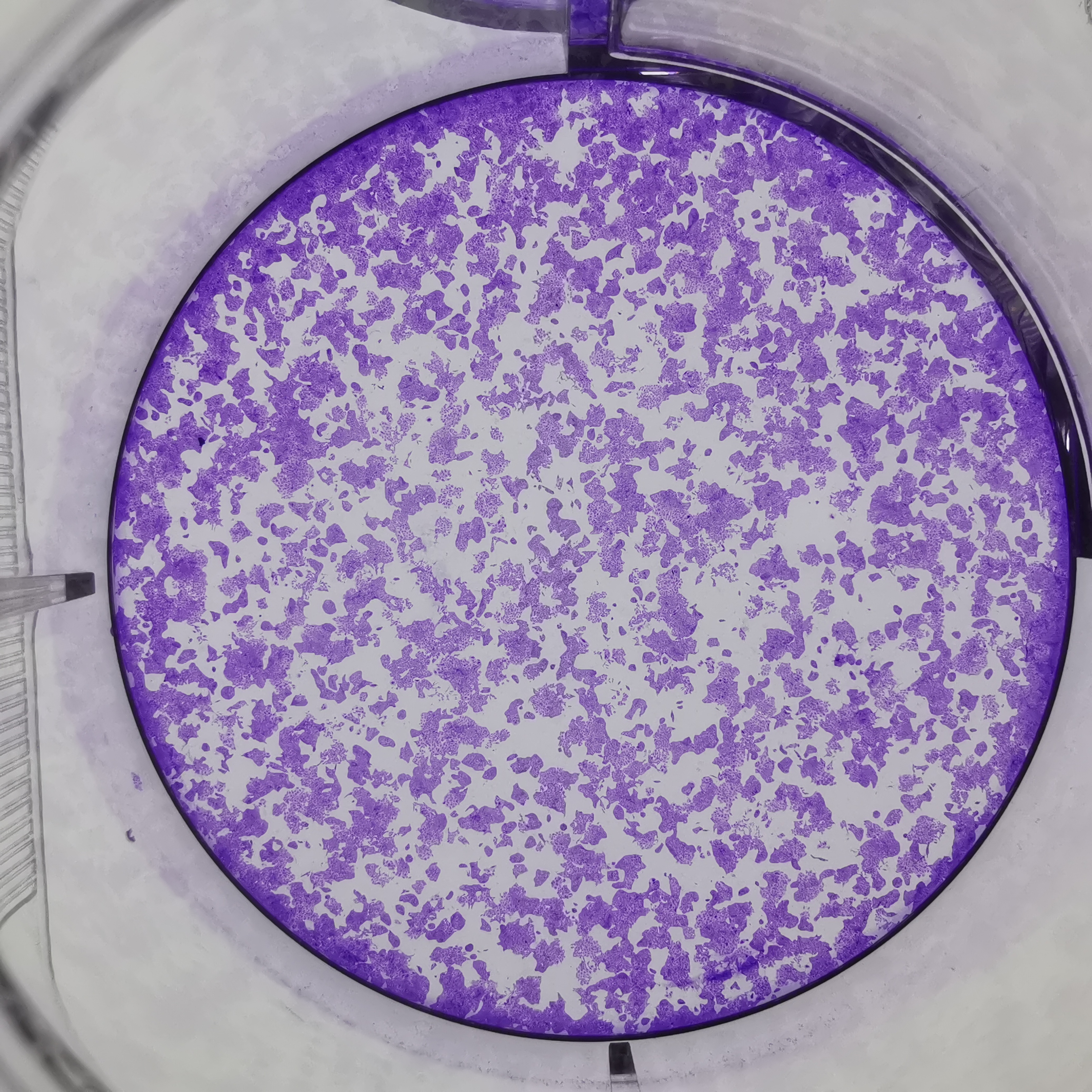

Supplement: Supplementary file 4 — Source data Fig. 2 [file 44319_2024_290_MOESM4_ESM.zip › 2B/Figure 2B-replicate/RCC4/RCC4 5000 PLKO.1 (1).jpg]

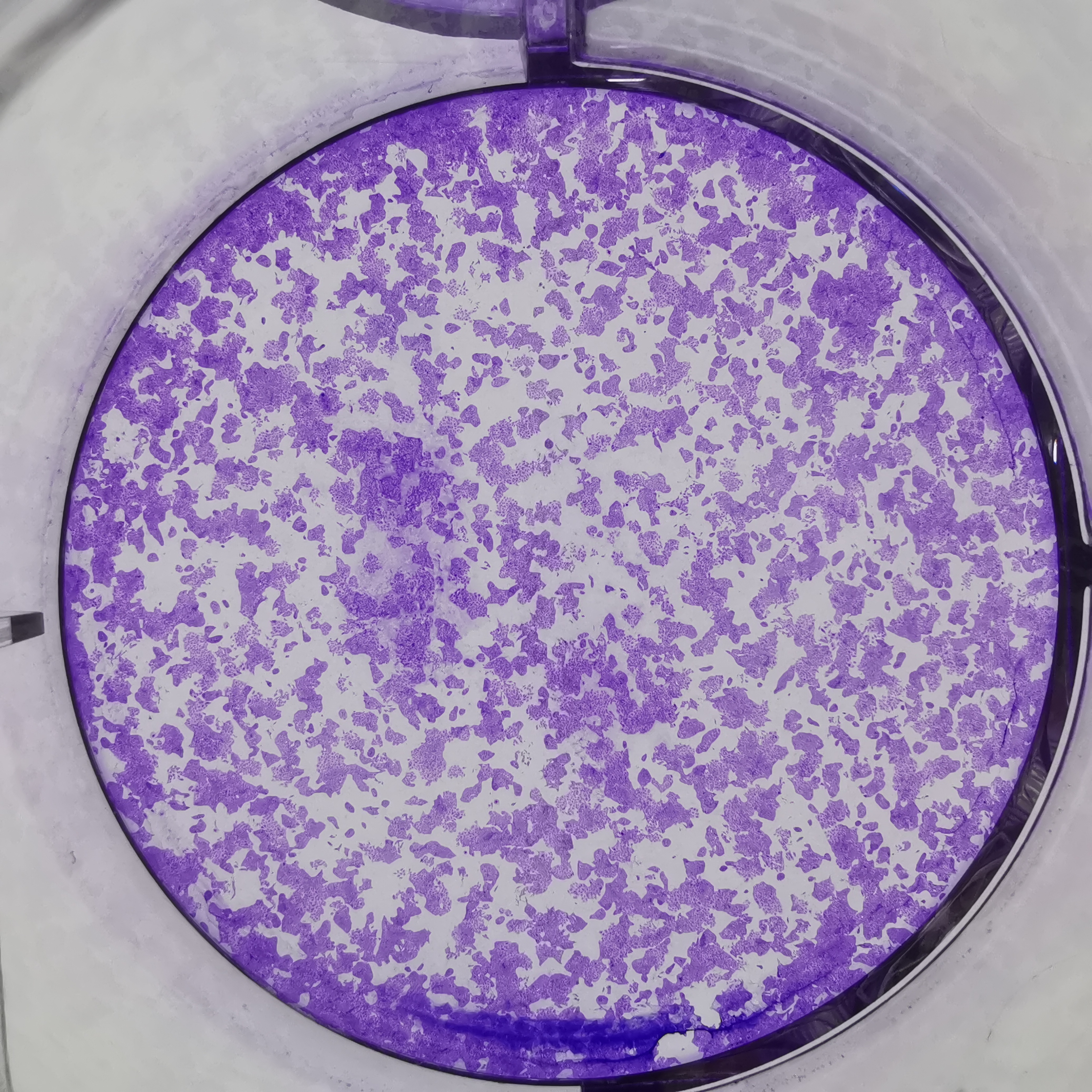

Supplement: Supplementary file 4 — Source data Fig. 2 [file 44319_2024_290_MOESM4_ESM.zip › 2B/Figure 2B-replicate/RCC4/RCC4 5000 PLKO.1 (2).jpg]

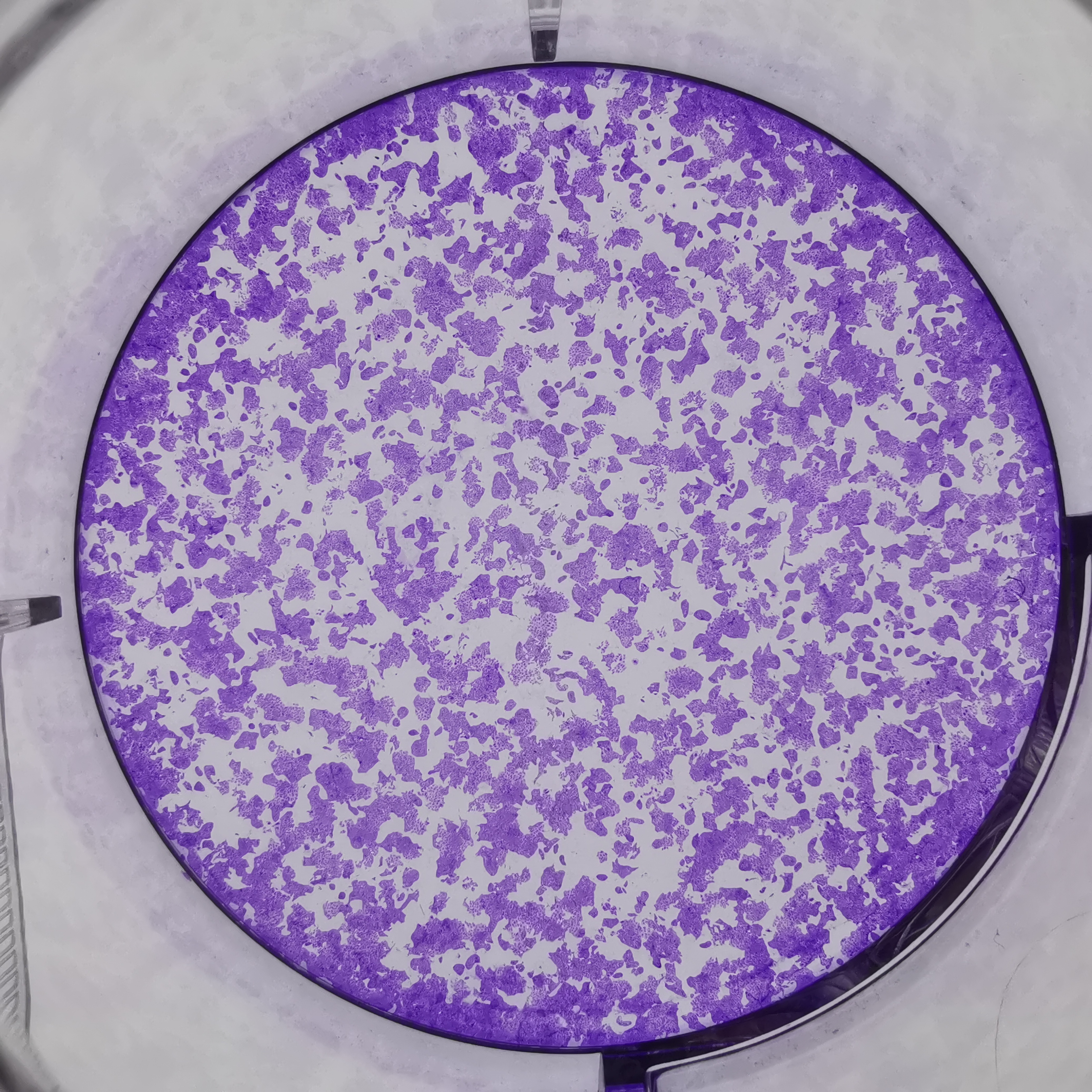

Supplement: Supplementary file 4 — Source data Fig. 2 [file 44319_2024_290_MOESM4_ESM.zip › 2B/Figure 2B-replicate/RCC4/RCC4 5000 PLKO.1 (3).jpg]

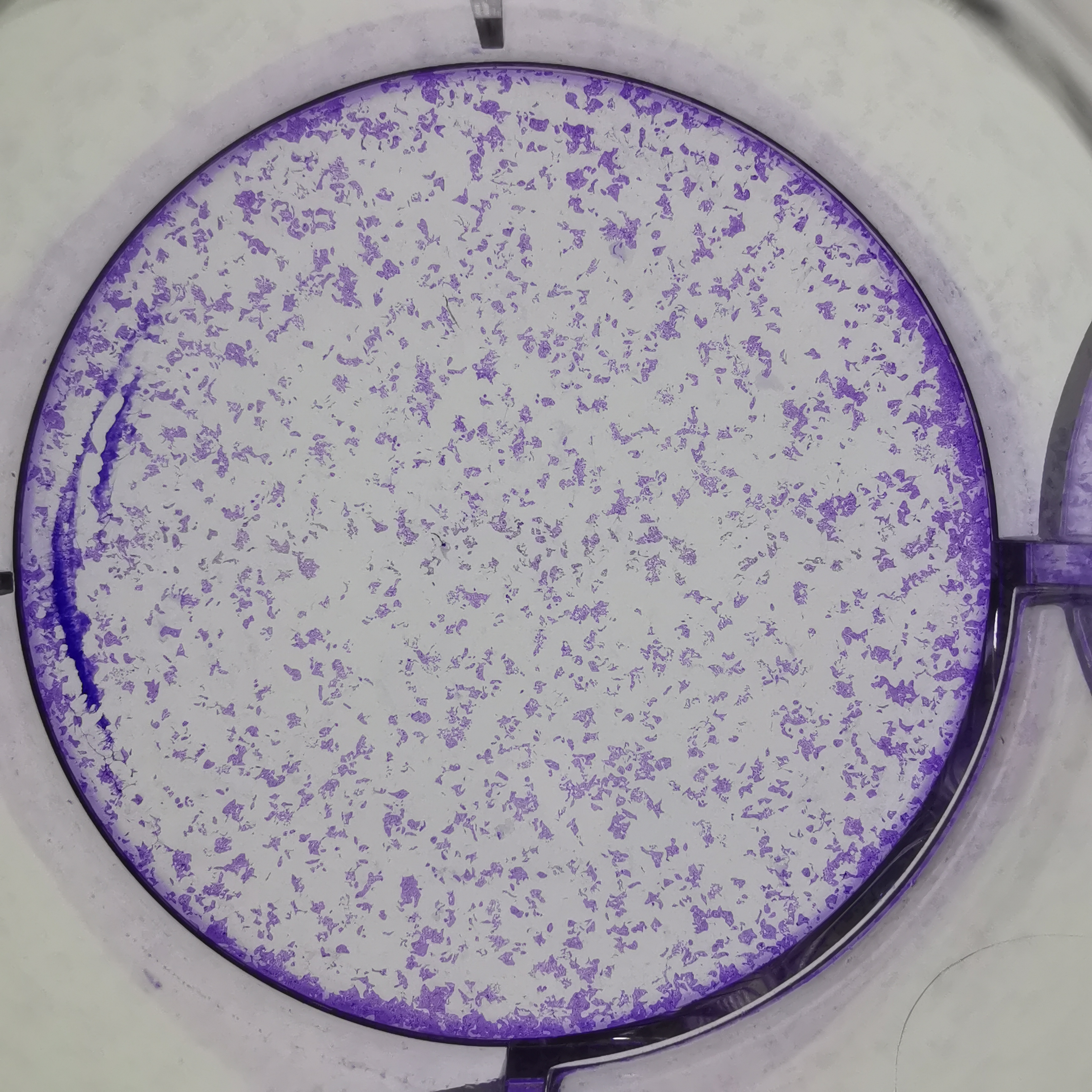

Supplement: Supplementary file 4 — Source data Fig. 2 [file 44319_2024_290_MOESM4_ESM.zip › 2B/Figure 2B-replicate/RCC4/RCC4 5000 SHYY1-4 (1).jpg]

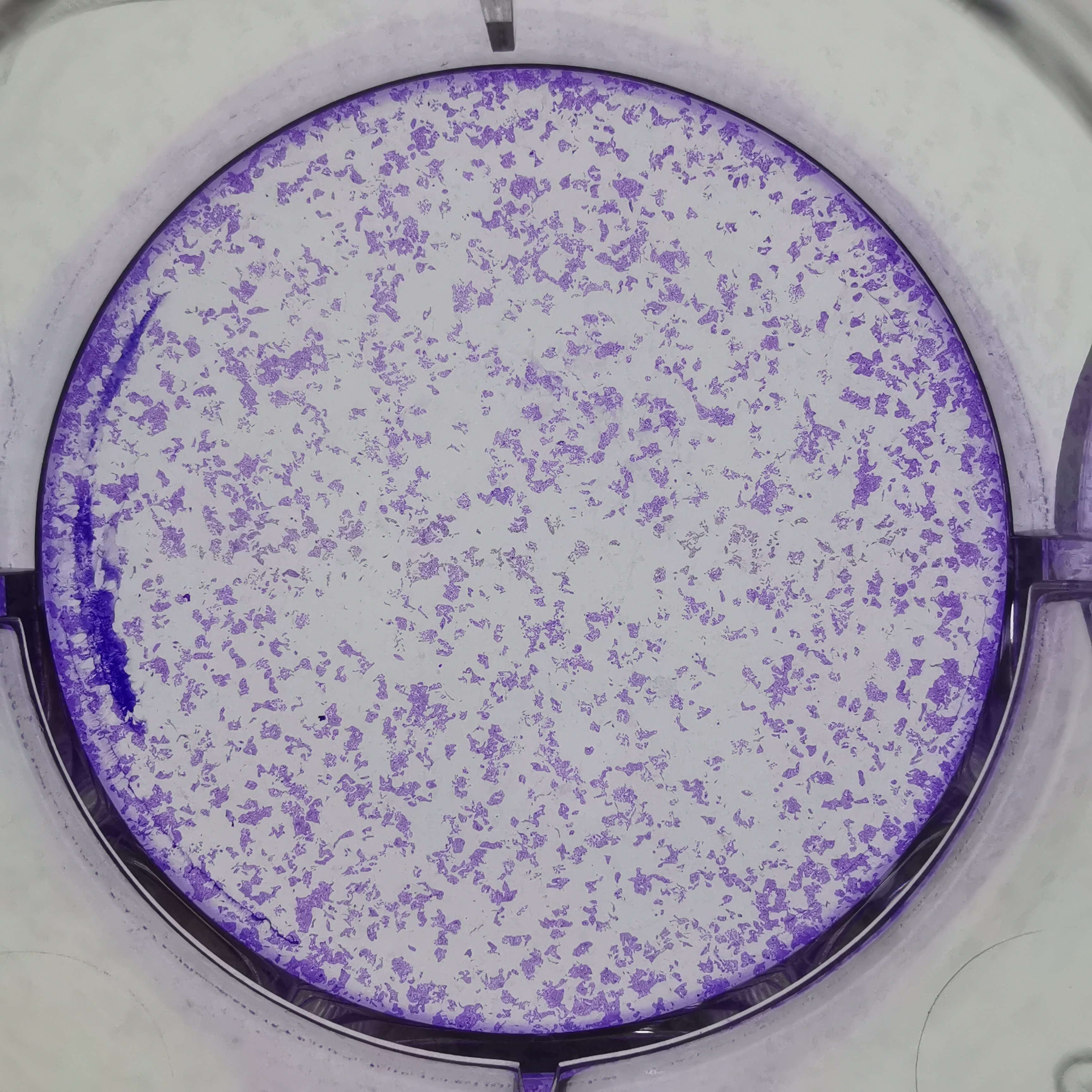

Supplement: Supplementary file 4 — Source data Fig. 2 [file 44319_2024_290_MOESM4_ESM.zip › 2B/Figure 2B-replicate/RCC4/RCC4 5000 SHYY1-4 (2).jpg]

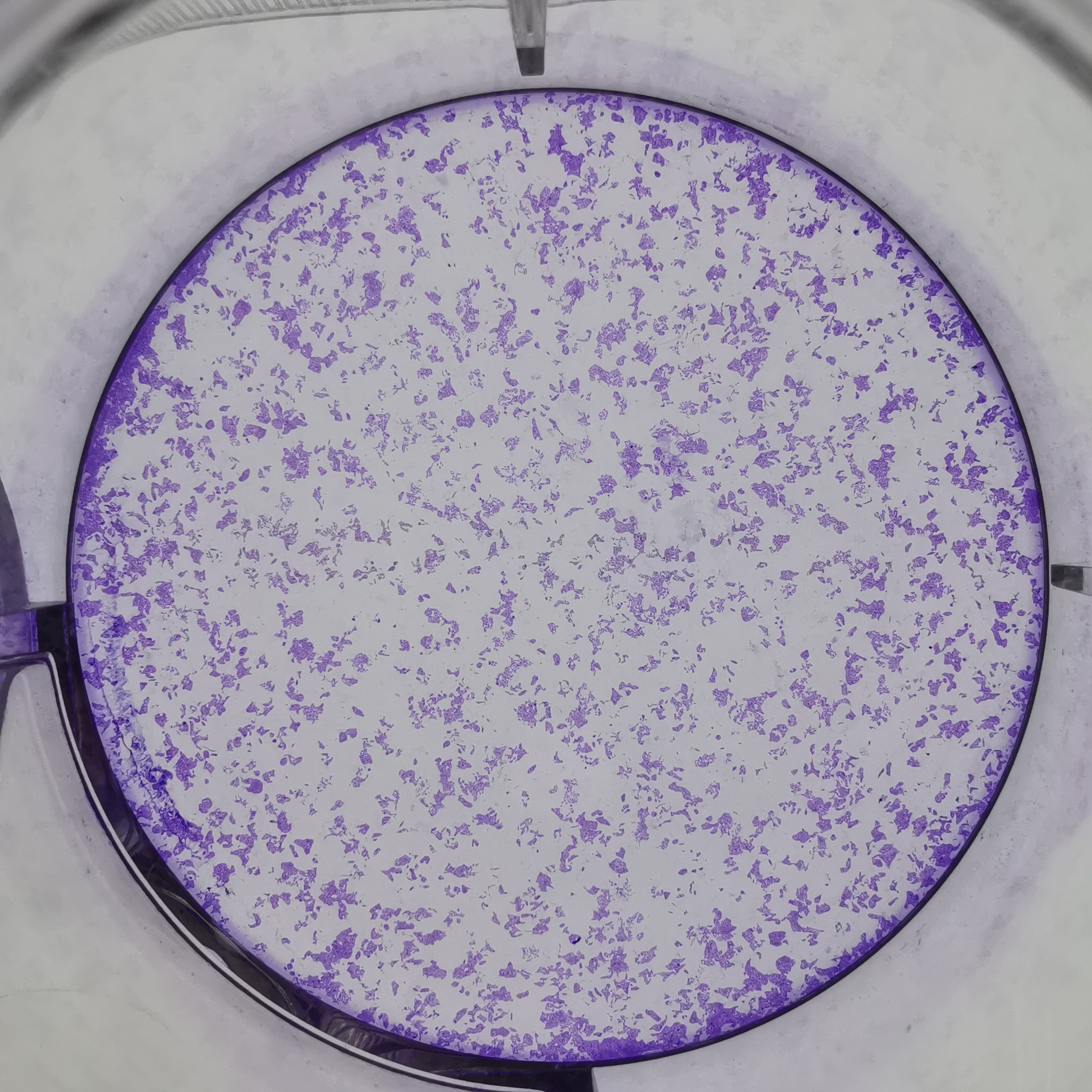

Supplement: Supplementary file 4 — Source data Fig. 2 [file 44319_2024_290_MOESM4_ESM.zip › 2B/Figure 2B-replicate/RCC4/RCC4 5000 SHYY1-4 (3).jpg]

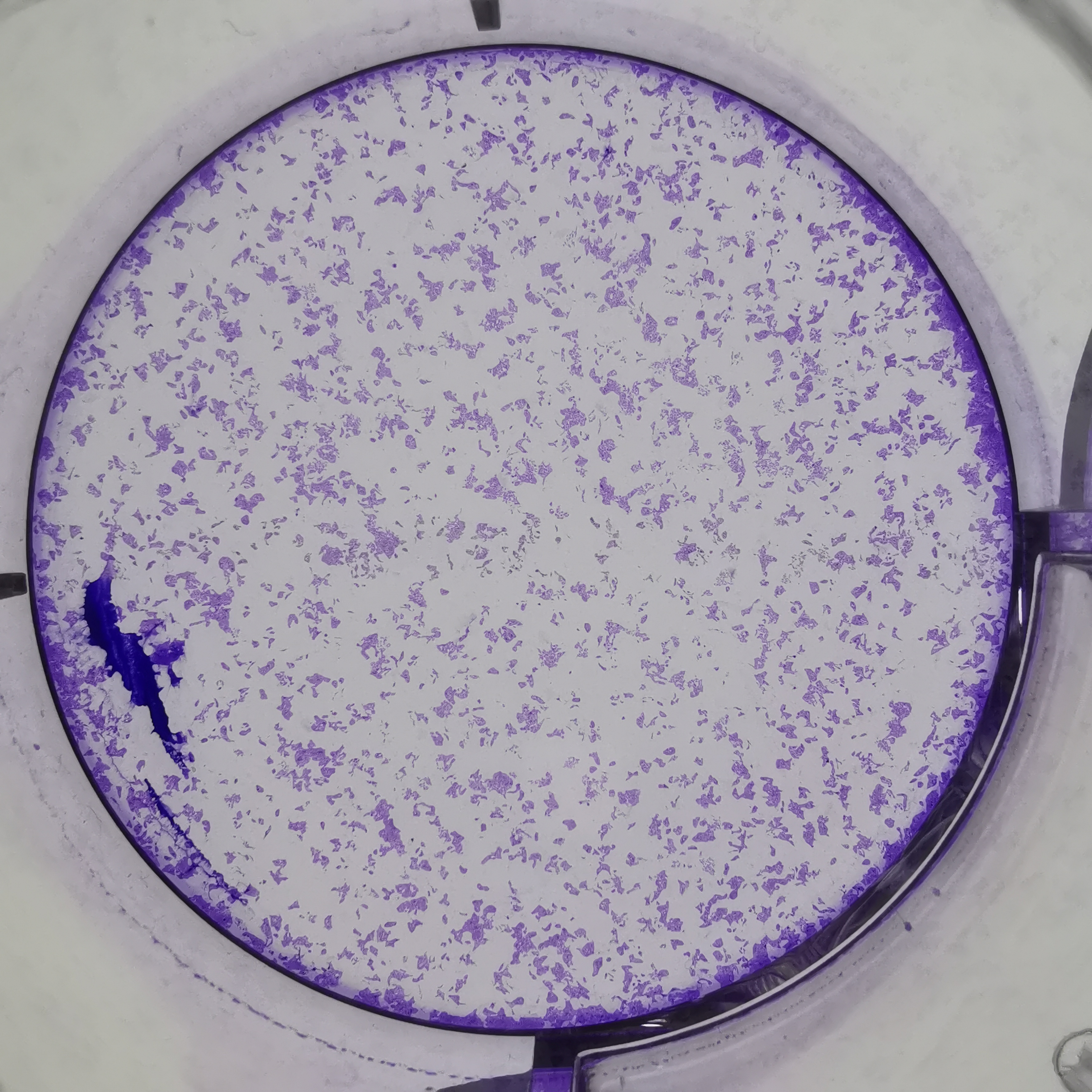

Supplement: Supplementary file 4 — Source data Fig. 2 [file 44319_2024_290_MOESM4_ESM.zip › 2B/Figure 2B-replicate/RCC4/RCC4 5000 SHYY1-5 (1).jpg]

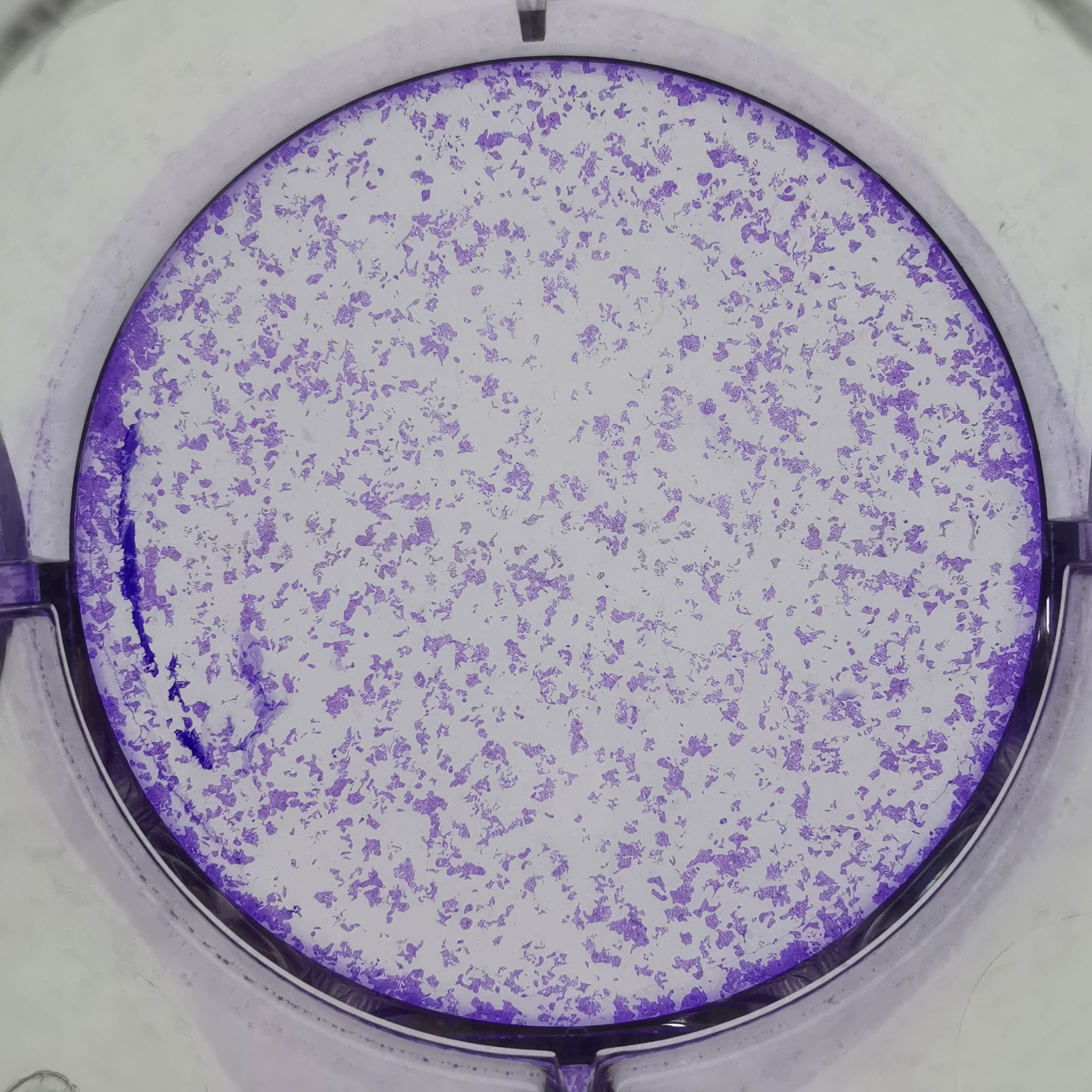

Supplement: Supplementary file 4 — Source data Fig. 2 [file 44319_2024_290_MOESM4_ESM.zip › 2B/Figure 2B-replicate/RCC4/RCC4 5000 SHYY1-5 (2).jpg]

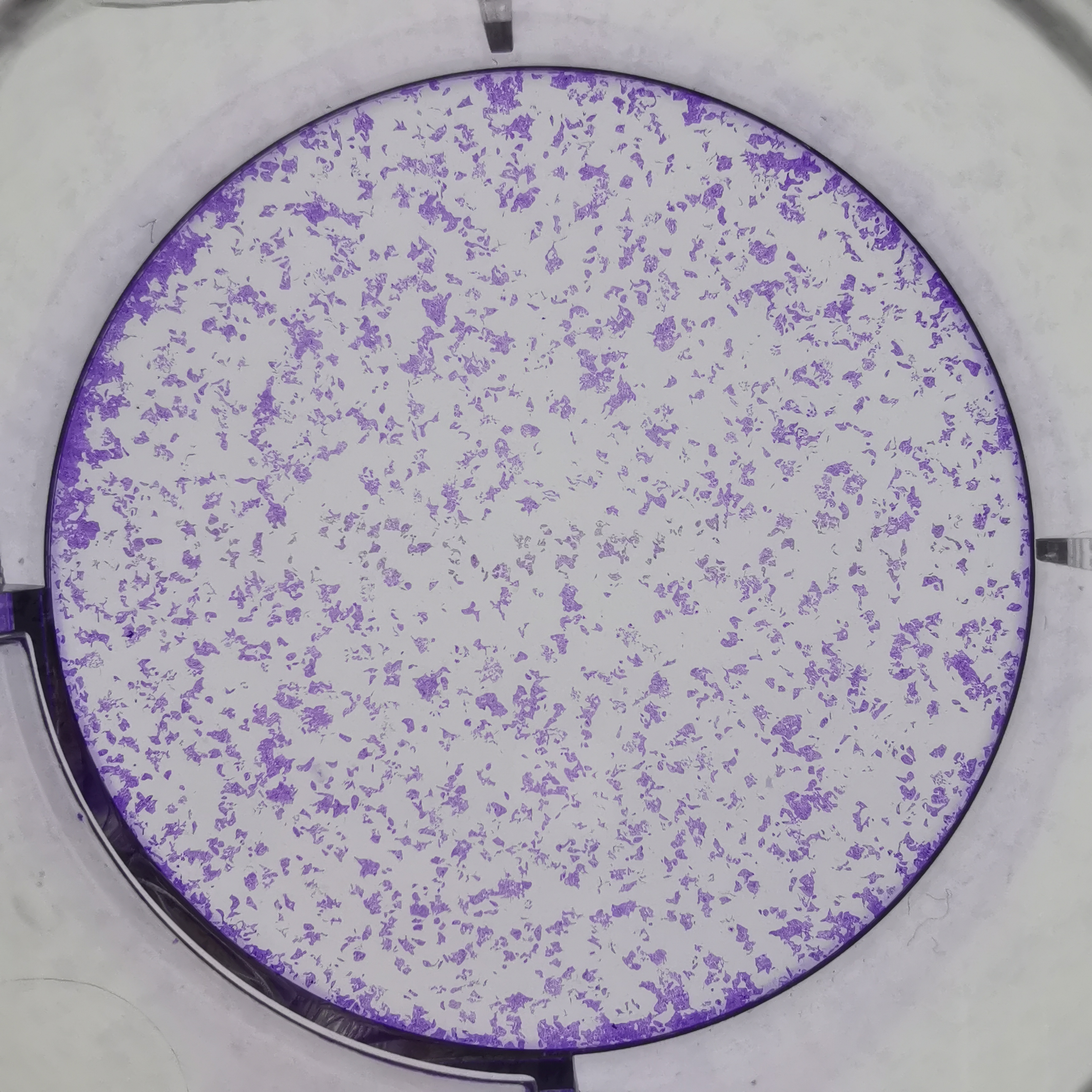

Supplement: Supplementary file 4 — Source data Fig. 2 [file 44319_2024_290_MOESM4_ESM.zip › 2B/Figure 2B-replicate/RCC4/RCC4 5000 SHYY1-5 (3).jpg]

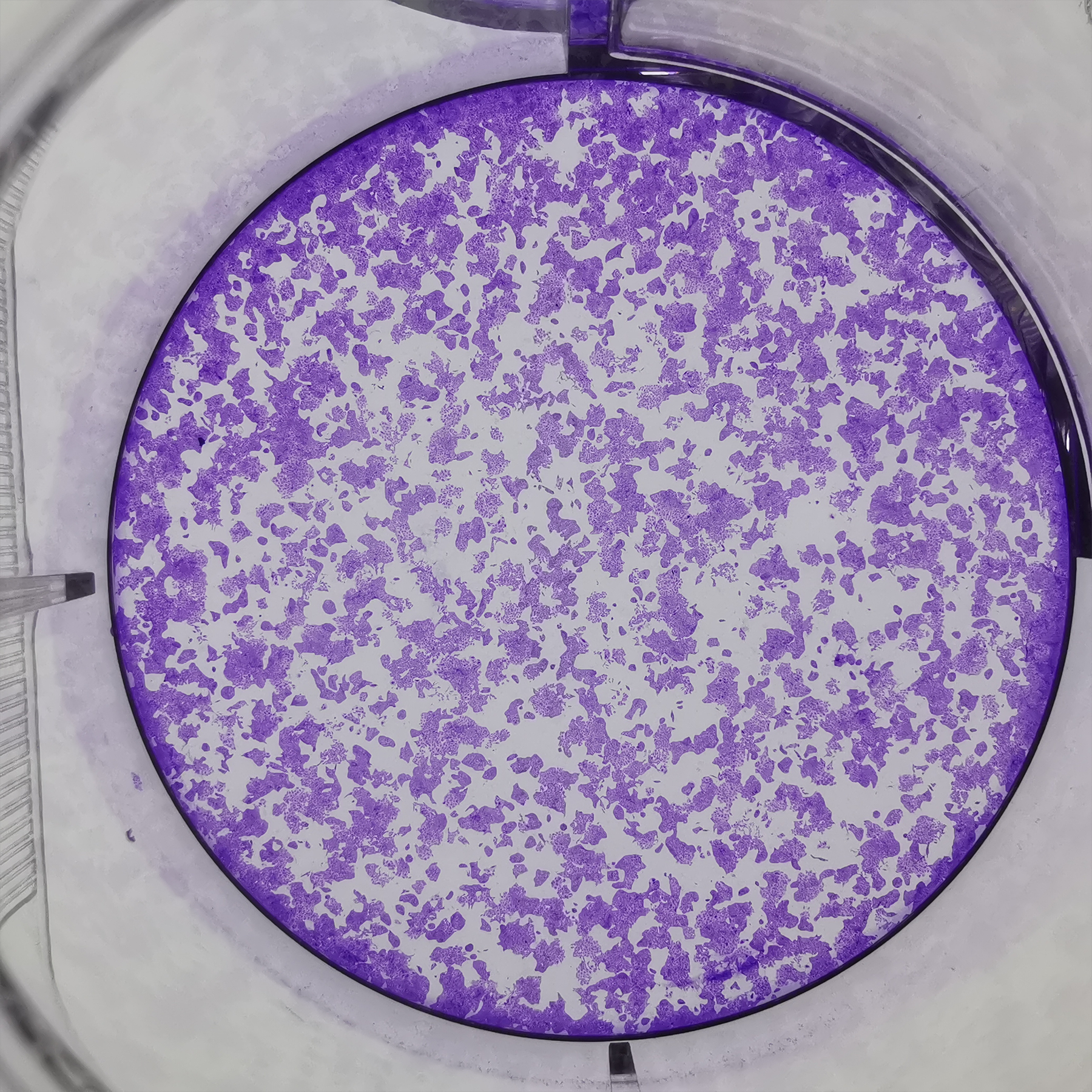

Supplement: Supplementary file 4 — Source data Fig. 2 [file 44319_2024_290_MOESM4_ESM.zip › 2B/RCC4/shCtrl.tif]

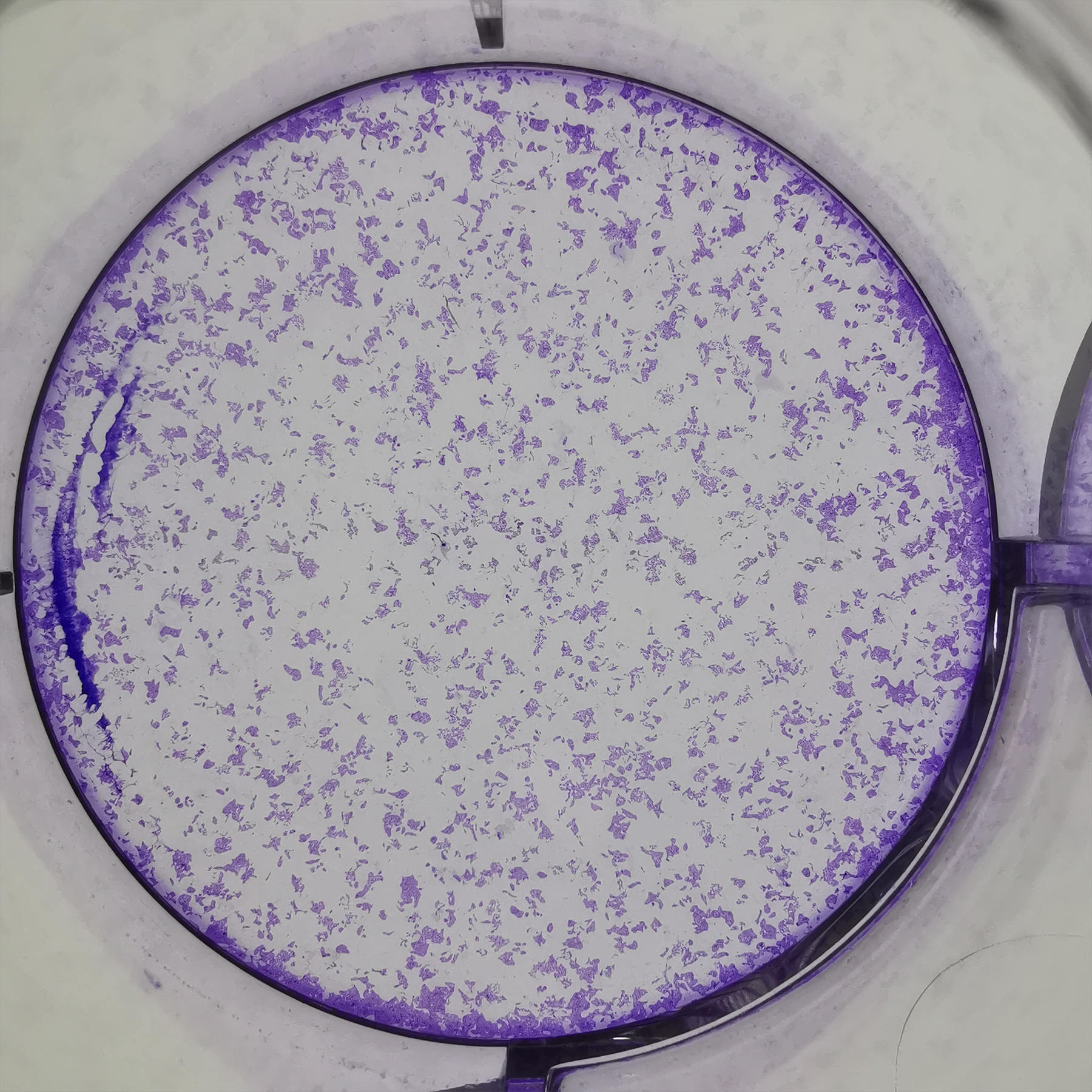

Supplement: Supplementary file 4 — Source data Fig. 2 [file 44319_2024_290_MOESM4_ESM.zip › 2B/RCC4/shYY1-1.tif]

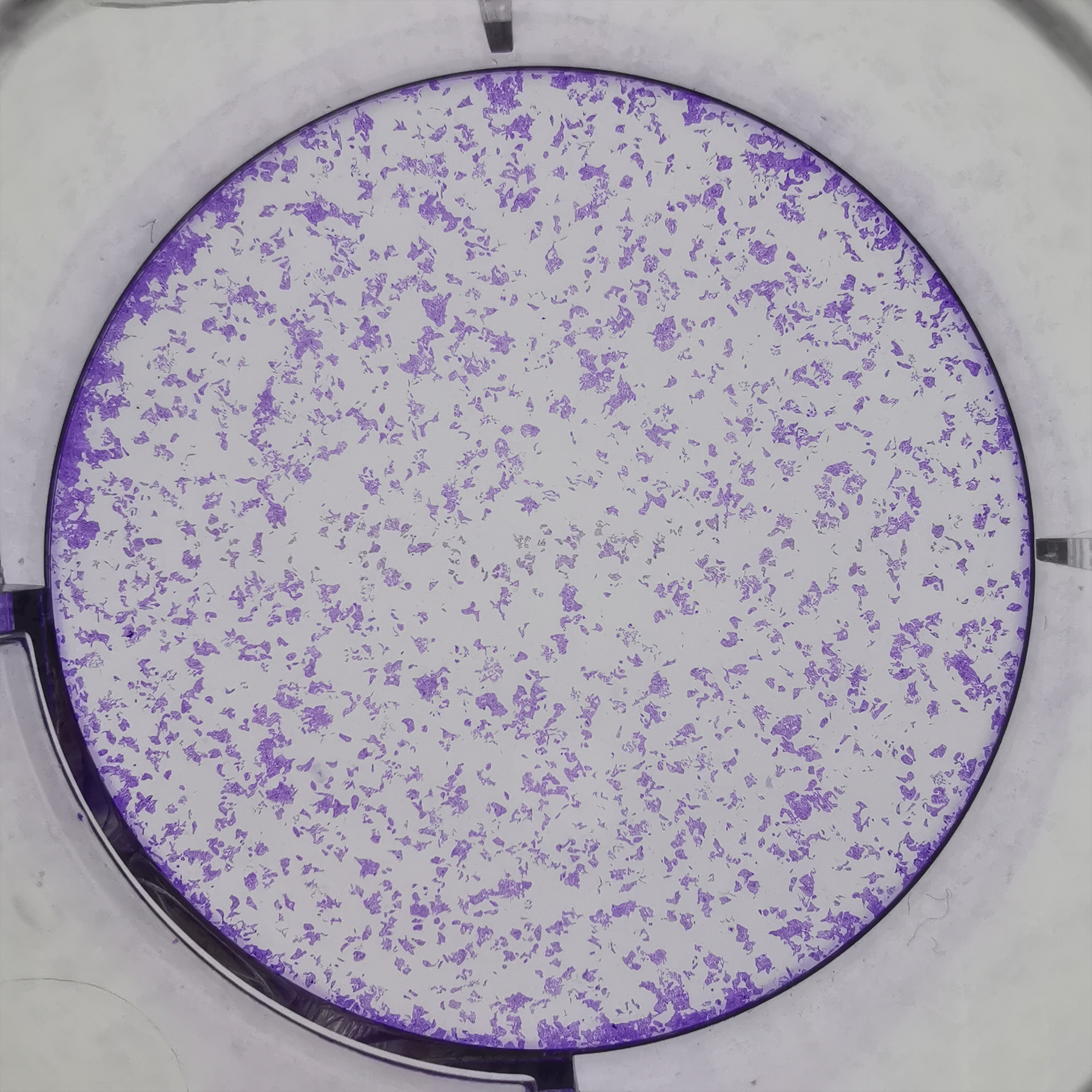

Supplement: Supplementary file 4 — Source data Fig. 2 [file 44319_2024_290_MOESM4_ESM.zip › 2B/RCC4/shYY1-2.tif]

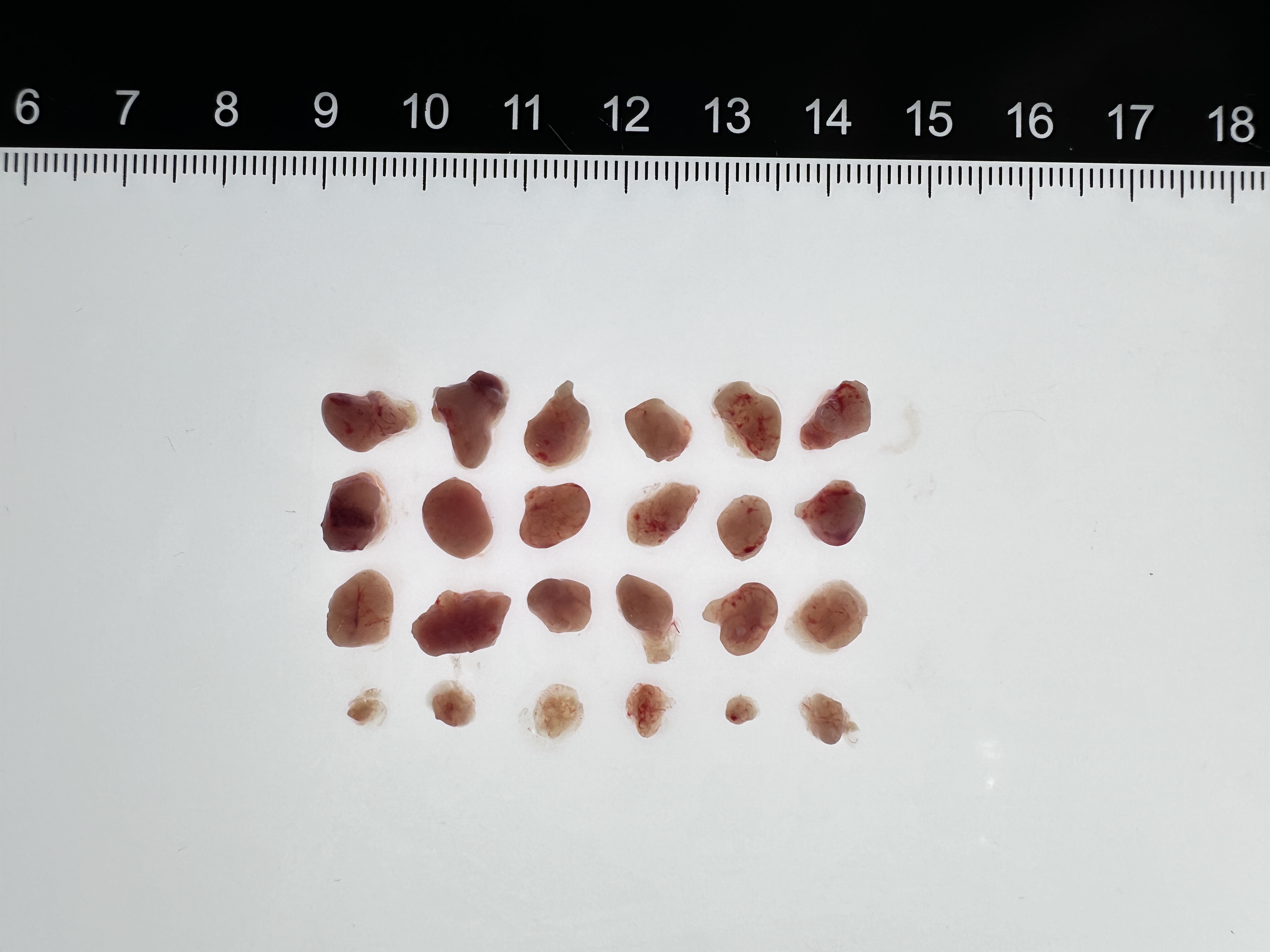

Supplement: Supplementary file 4 — Source data Fig. 2 [file 44319_2024_290_MOESM4_ESM.zip › 2D/2D.tif]

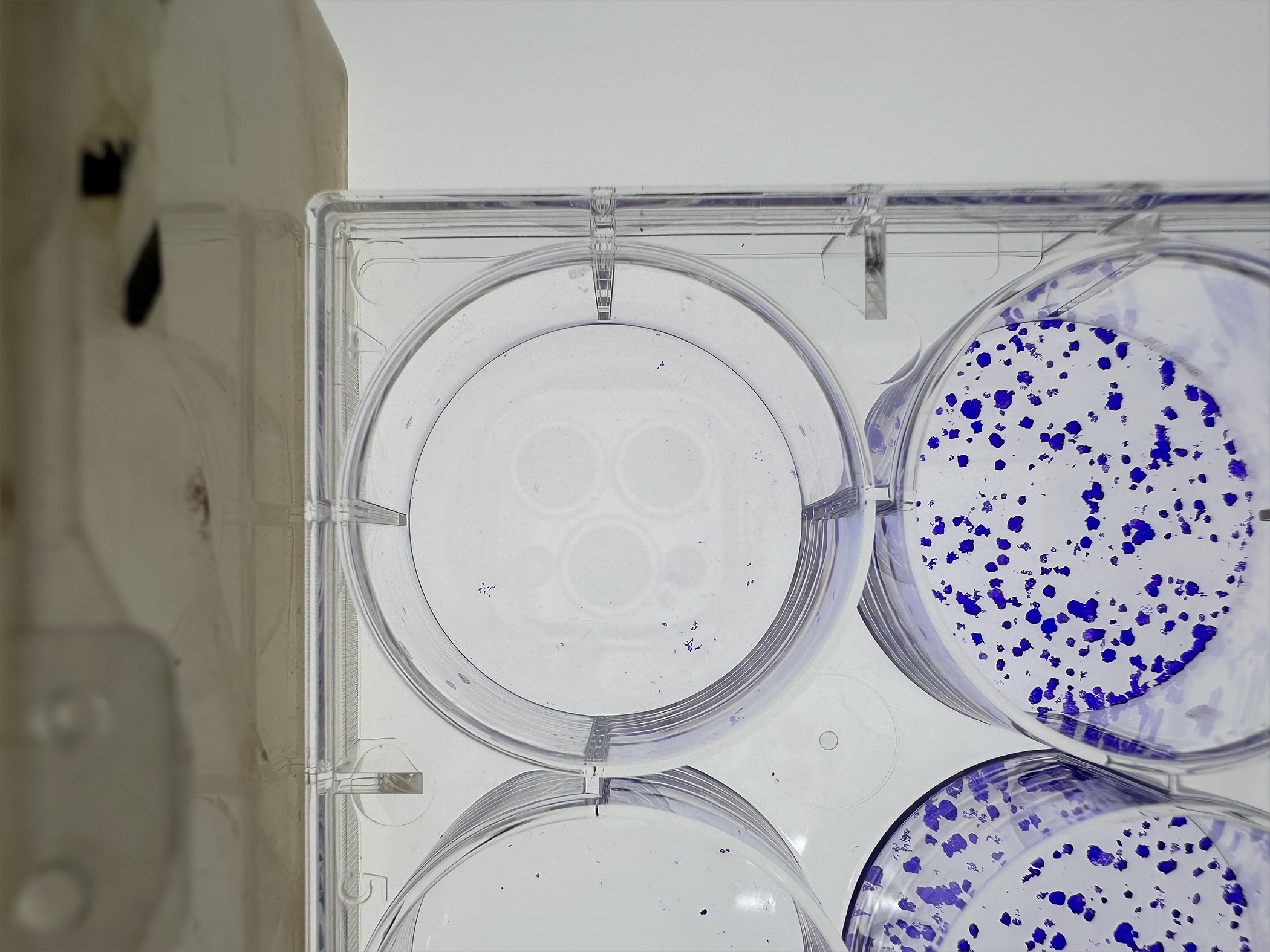

Supplement: Supplementary file 6 — Source data Fig. 4 [file 44319_2024_290_MOESM6_ESM.zip › 4J/dsh .tif]

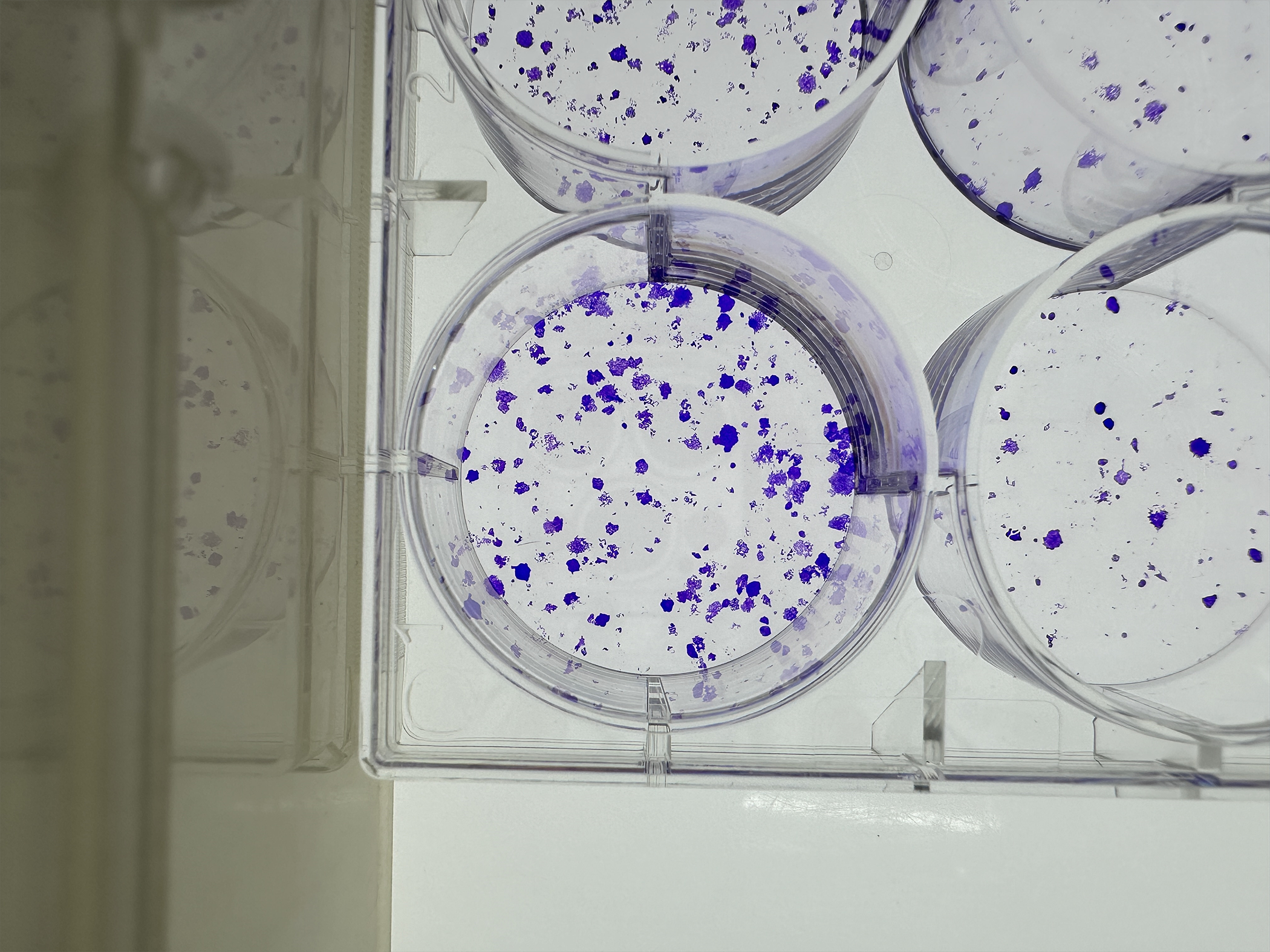

Supplement: Supplementary file 6 — Source data Fig. 4 [file 44319_2024_290_MOESM6_ESM.zip › 4J/dsh+5c.tif]

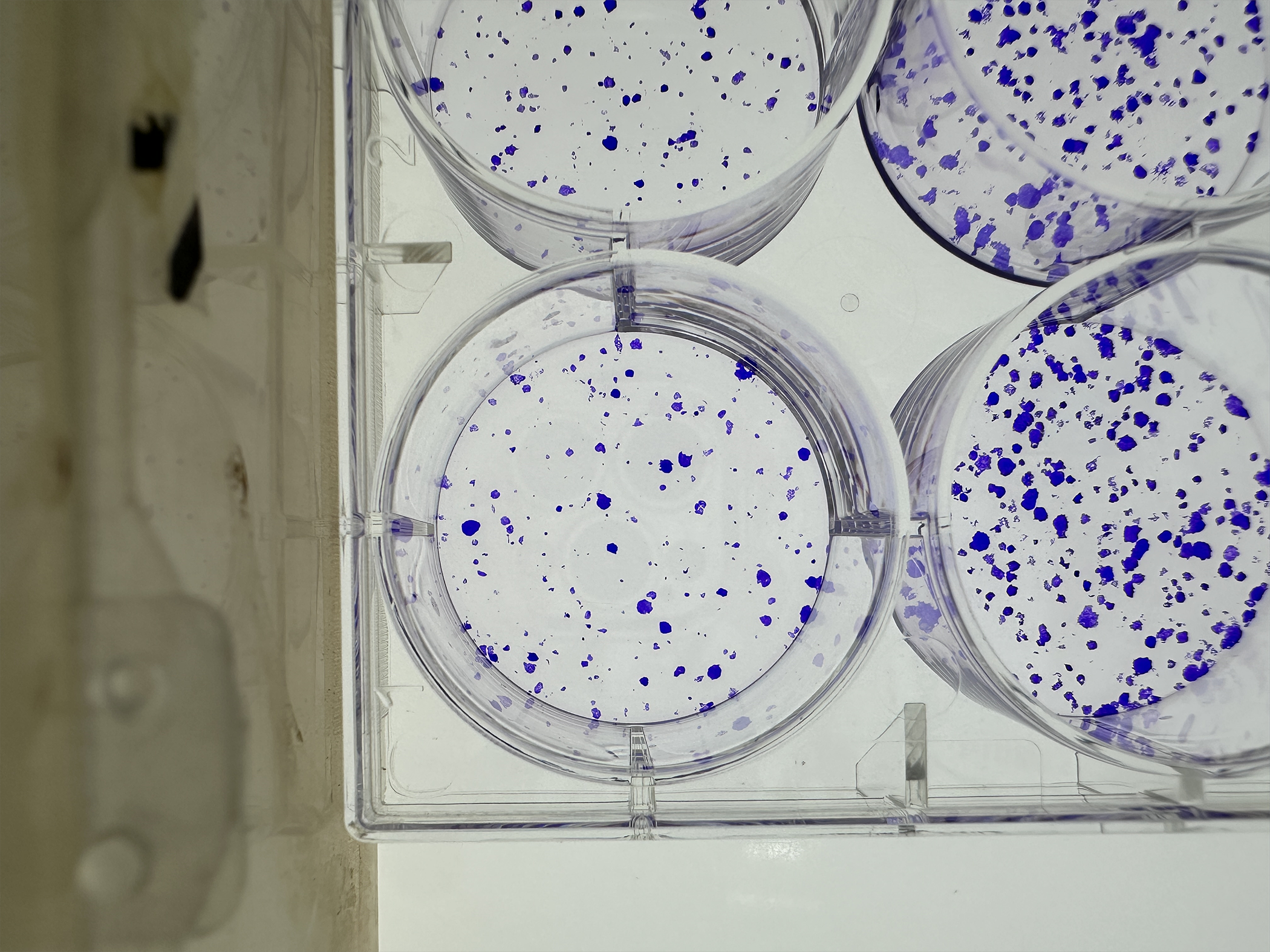

Supplement: Supplementary file 6 — Source data Fig. 4 [file 44319_2024_290_MOESM6_ESM.zip › 4J/dsh+H514A.tif]

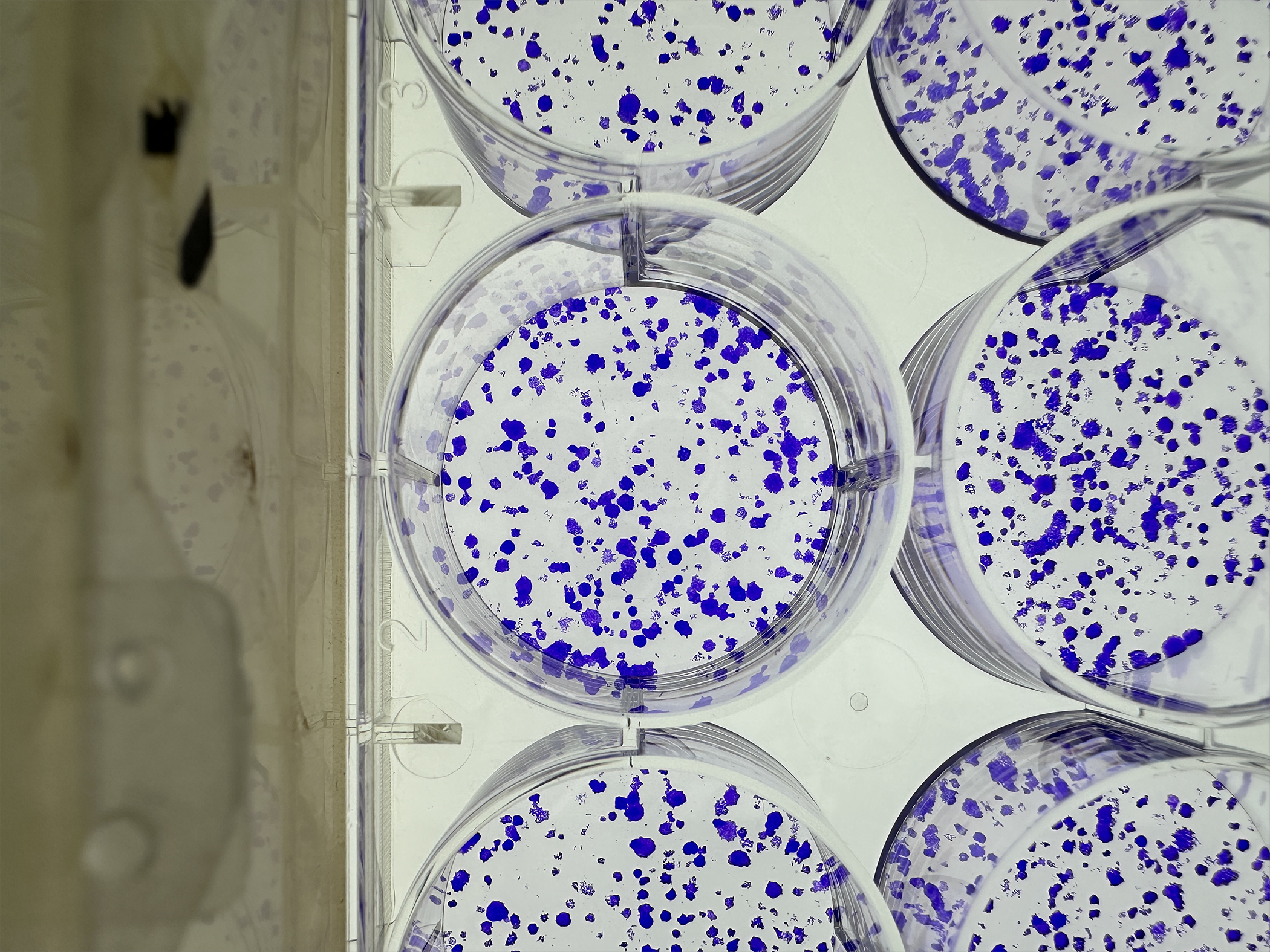

Supplement: Supplementary file 6 — Source data Fig. 4 [file 44319_2024_290_MOESM6_ESM.zip › 4J/shCtrl.tif]

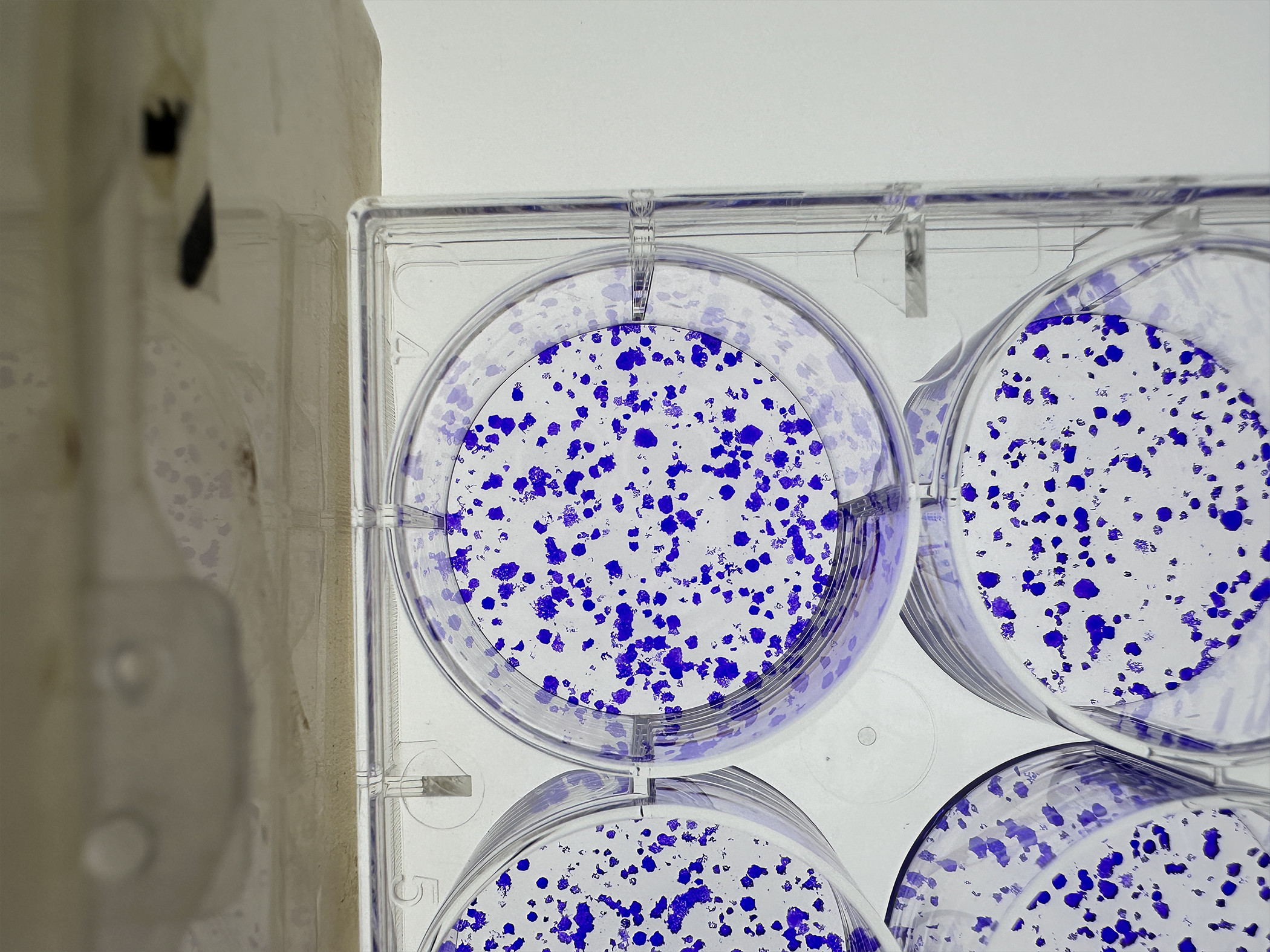

Supplement: Supplementary file 6 — Source data Fig. 4 [file 44319_2024_290_MOESM6_ESM.zip › 4J/shKDM5C.tif]

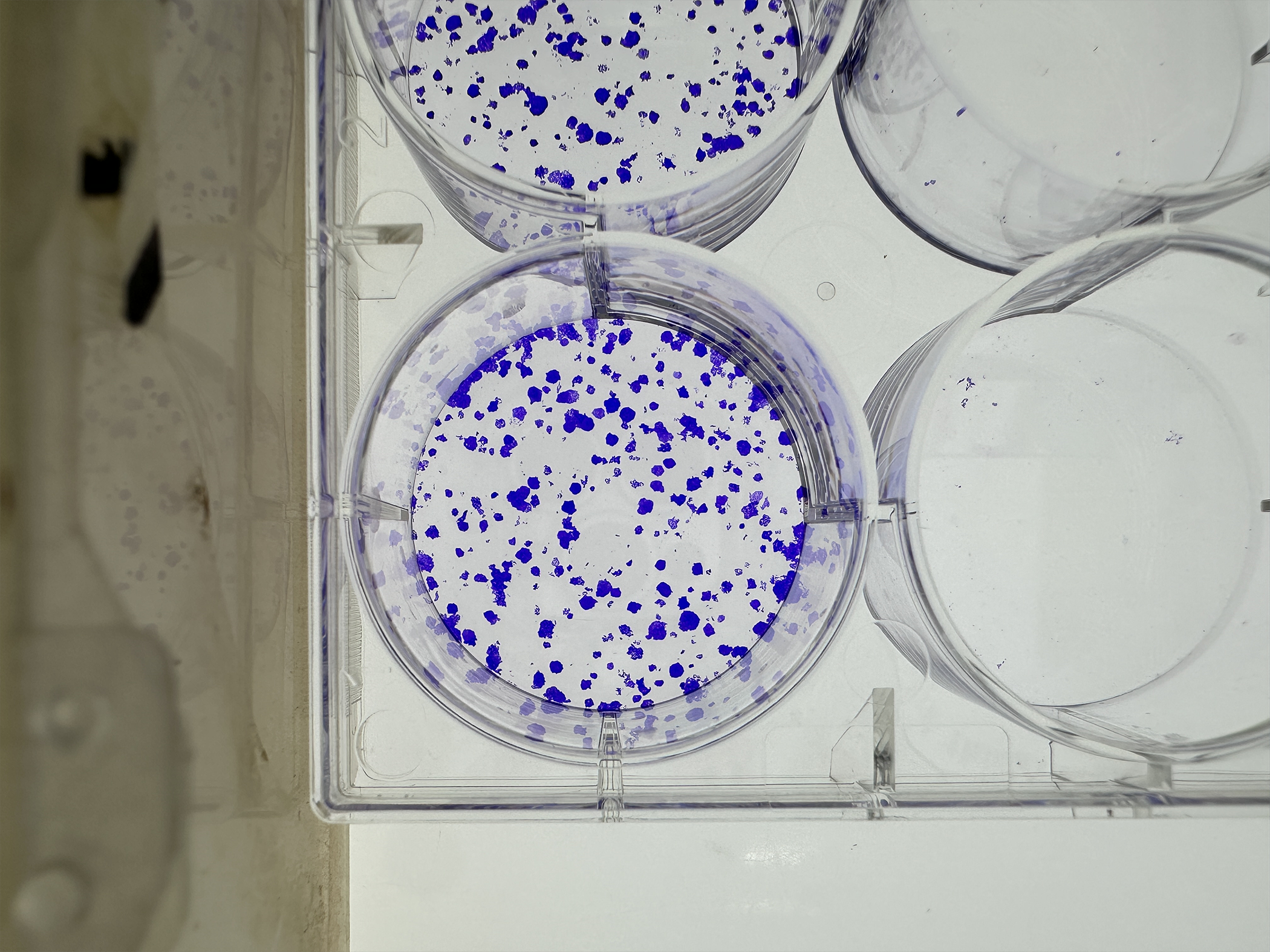

Supplement: Supplementary file 6 — Source data Fig. 4 [file 44319_2024_290_MOESM6_ESM.zip › 4J/shYY1.tif]

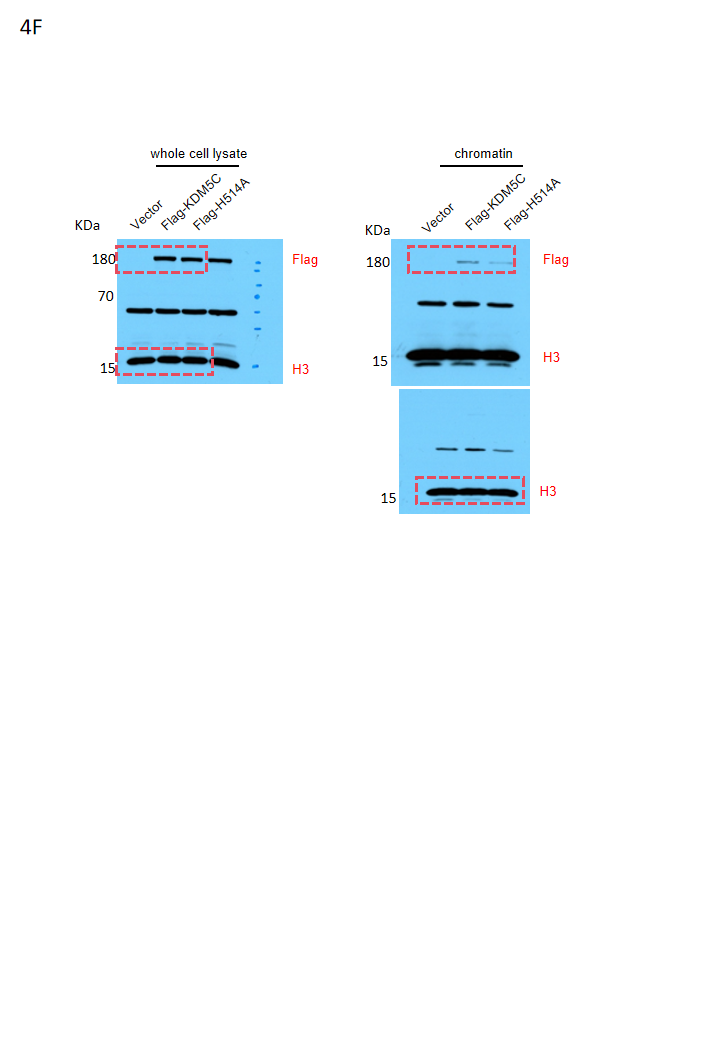

Supplement: Supplementary file 6 — Source data Fig. 4 [file 44319_2024_290_MOESM6_ESM.zip › 4F/4F.tif]
