# Supplementary material for: Targeting the transcription factor YY1 is synthetic lethal with loss of the histone demethylase KDM5C
Source: EMBO Rep. 2024 Oct 21;25(12):5408–28. doi: 10.1038/s44319-024-00290-8 (PMC11624269; doi:10.1038/s44319-024-00290-8)
Supplement: Supplementary file 5 — Source data Fig. 3 [file 44319_2024_290_MOESM5_ESM.zip › 3B/README.docx]

The sequencing data are available in GEO Data Sets with the accession number GSE275838 (https://www.ncbi.nlm.nih.gov/search/all/?term=GSE275838).
