# Supplementary material for: Targeting the transcription factor YY1 is synthetic lethal with loss of the histone demethylase KDM5C
Source: EMBO Rep. 2024 Oct 21;25(12):5408–28. doi: 10.1038/s44319-024-00290-8 (PMC11624269; doi:10.1038/s44319-024-00290-8)
Supplement: Supplementary file 9 — Expanded View Figures [file 44319_2024_290_MOESM9_ESM.pdf]

Expanded View Figures

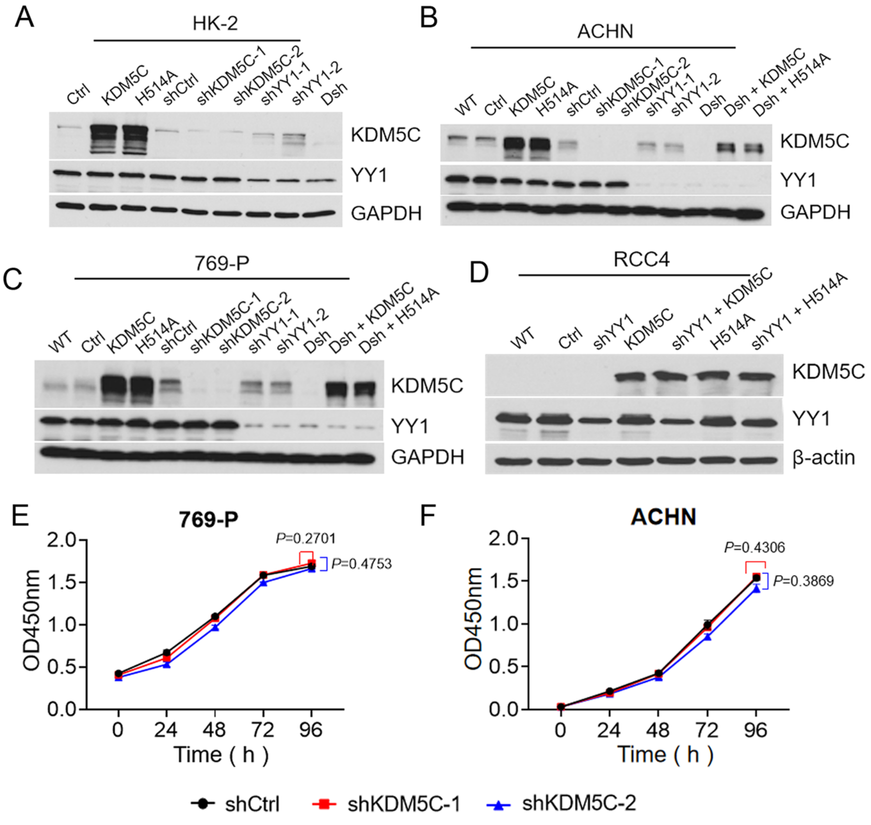

Figure EV1. Validation of the constructed cell lines used in this study.

(**A–D**) KDM5C and YY1 protein levels in the indicated cells were measured via western blotting. "Dsh" indicates cells in which KDM5C and YY1 were both knocked down. (**E**, **F**) Proliferation rate of the indicated cell lines upon KDM5C depletion, as determined via a CCK8 assay. The data are shown as the mean ± SD of three independent experiments after analysis via one-way ANOVA. In 769-P cells, $P = 0.2701$ (shKDM5C -1) and $P = 0.4753$ (shKDM5C-2); in ACHN cells, $P = 0.4306$ (shKDM5C-1) and $P = 0.3869$ (shYY1-2). Source data are available online for this figure.

© The Author(s)

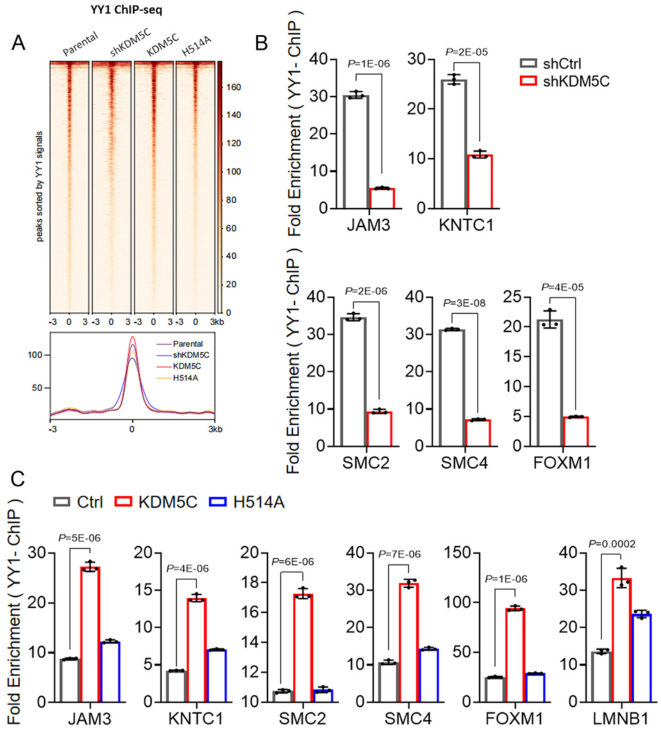

Figure EV2. ChIP-seq experiments targeting YY1 in HK-2 cells.

(**A**) Heatmap and metaplot showing the chromatin enrichment of YY1 in HK-2 cells in the presence or absence of KDM5C. (**B**, **C**) ChIP-PCR assay showing the enrichment of YY1 at the promoters of the indicated genes in HK-2 cells after the depletion of YY1, KDM5C or both (**B**), or in cells expressing KDM5C-WT or KDM5C-H514A mutant (**C**). Each experiment was repeated three times with similar results. Statistical significance was determined by one-way ANOVA. (**B**) $P = 1.24E-06$ (JAM3), $P = 2.27E-06$ (KNTC1), $P = 2.82E-08$ (SMC2), $P = 2.41E-05$ (SMC4) and $P = 3.96E-05$ (FOXM1), respectively. (**C**) $P = 4.53E-06$ (JAM3), $P = 4.46E-06$ (KNTC1), $P = 5.53E-06$(SMC2), $P = 6.82E-06$ (SMC4), $P = 1.05E-06$ (FOXM1) and $P = 0.0002$ (LMNB1), respectively. Source data are available online for this figure.

 © The Author(s)

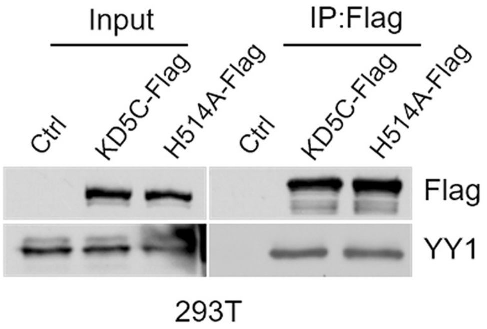

Figure EV3. Evaluation of the interaction between YY1 and KDM5C-WT or KDM5C-H514A mutant.

Co-IP followed by western blotting showing the interaction between YY1 and KDM5C-WT or KDM5C-H514A mutant in HEK293T cells. Source data are available online for this figure.

© The Author(s)

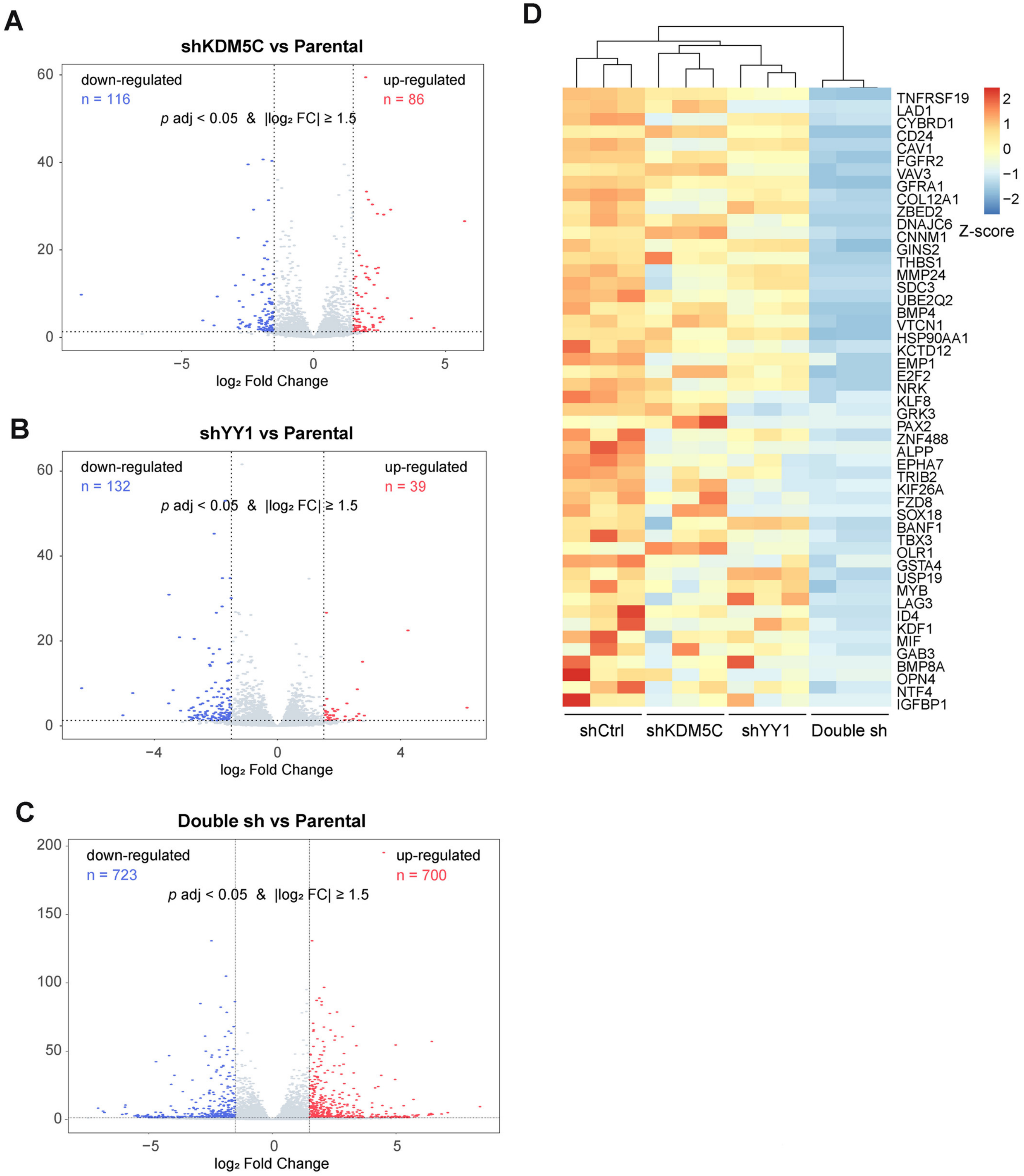

Figure EV4. RNA-seq in HK-2 derived cells.

(**A–C**) Volcano plots showing the change in the number of transcripts in shKDM5C, shYY1 or double-knockdown HK-2 cells, as determined by RNA-seq analysis (| $\log_2$ FC | > 1.5, *P* value < 0.05). (**D**) Heatmap showing the normalized gene expression profile in shKDM5C, shYY1 or double sh ACHN cells. "Double sh" indicates cells in which KDM5C and YY1 were both knocked down. Source data are available online for this figure.

© The Author(s)

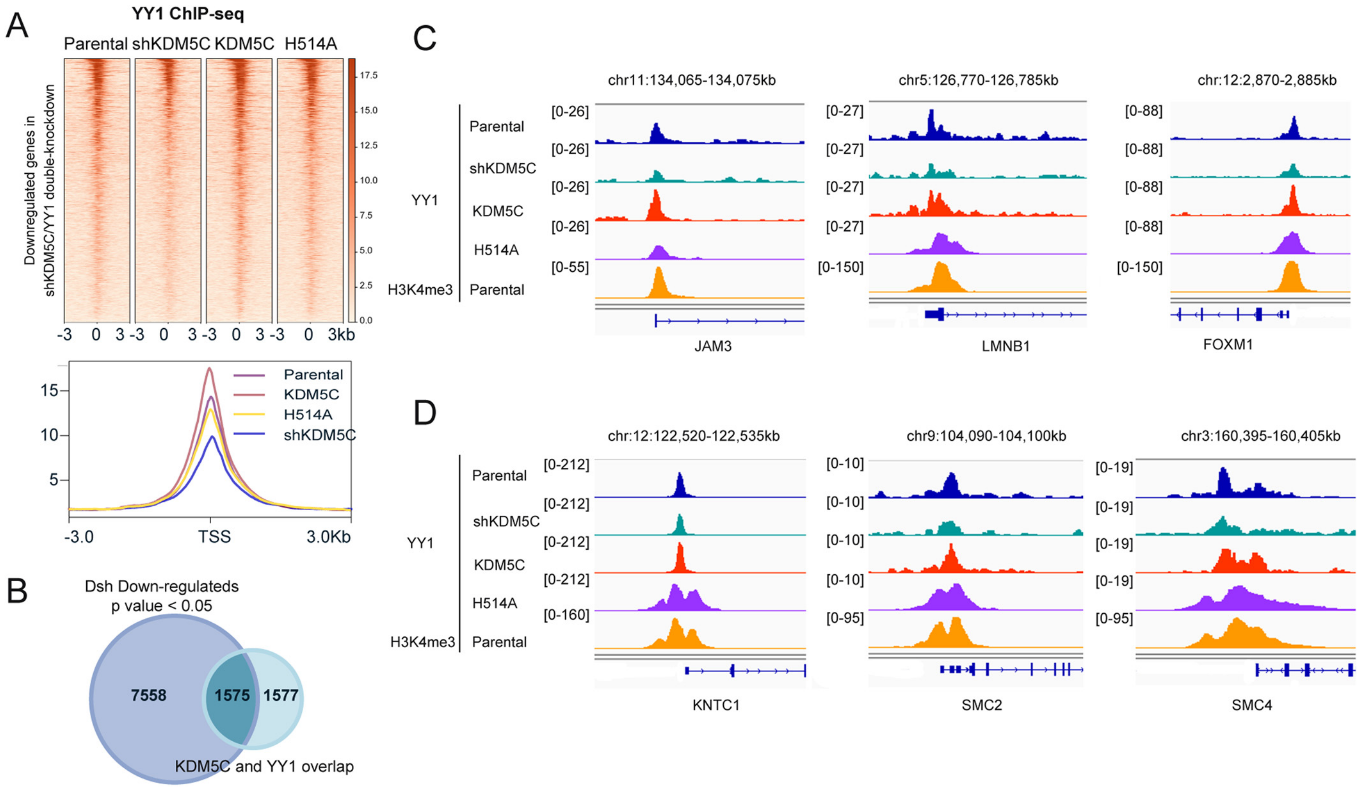

Figure EV5. Effects of KDM5C on YY1 enrichment at promoter regions of specific genes.

(**A**, **B**) The connections between the ChIP-seq and RNA-seq data were analyzed, and the genomic regions of the downregulated genes in the KDM5C/YY1 double-knockdown group were annotated. (**C**, **D**) ChIP-seq snapshots reflecting changes in YY1 enrichment at the promoter regions of the JAM3, LNMB1, FOXM1, KNTC1, SMC2 and SMC4 genes in the indicated cell lines. The promoters are indicated by H3K4me3 peaks. Source data are available online for this figure.
